# Reversible chromism of spiropyran in the cavity of a flexible coordination cage

**DOI:** 10.1038/s41467-017-02715-6

**Published:** 2018-02-13

**Authors:** Dipak Samanta, Daria Galaktionova, Julius Gemen, Linda J. W. Shimon, Yael Diskin-Posner, Liat Avram, Petr Král, Rafal Klajn

**Affiliations:** 1 Department of Organic Chemistry, Weizmann Institute of Science, Rehovot, 76100 Israel; 2 Department of Chemistry, University of Illinois at Chicago, Chicago, IL 60607 USA; 3 Department of Chemical Research Support, Weizmann Institute of Science, Rehovot, 76100 Israel; 4 Department of Physics, University of Illinois at Chicago, Chicago, IL 60607 USA; 5 Department of Biopharmaceutical Sciences, University of Illinois at Chicago, Chicago, IL 60607 USA

## Abstract

Confining molecules to volumes only slightly larger than the molecules themselves can profoundly alter their properties. Molecular switches—entities that can be toggled between two or more forms upon exposure to an external stimulus—often require conformational freedom to isomerize. Therefore, placing these switches in confined spaces can render them non-operational. To preserve the switchability of these species under confinement, we work with a water-soluble coordination cage that is flexible enough to adapt its shape to the conformation of the encapsulated guest. We show that owing to its flexibility, the cage is not only capable of accommodating—and solubilizing in water—several light-responsive spiropyran-based molecular switches, but, more importantly, it also provides an environment suitable for the efficient, reversible photoisomerization of the bound guests. Our findings pave the way towards studying various molecular switching processes in confined environments.

## Abstract (web summary)

Under confinement, molecular switches lose the conformational freedom often needed to isomerize. Here, the authors show that a flexible coordination cage can adapt its shape to guide the photoisomerization of encapsulated spiropyrans, rendering them reversibly photochromic even within the confines of the cavity.

## Introduction

Placing various molecules in confined environments can markedly alter their chemical properties. Examples in nature are abundant and range from stabilizing ephemeral species (e.g., sulfenic acids^1^) to accelerating chemical reactions to great extents (such as the >10^7^ rate acceleration of CO_2_ hydrolysis by carbonic anhydrase^2^) to synthesizing highly complex molecules^3^ and unique inorganic nanostructures^4^. Inspired by these and numerous other examples, chemists have developed diverse families of synthetic confined spaces—such as nanopores of metal–organic frameworks^5^, microemulsion droplets^6^, and nanopores between densely packed nanoparticles^7^—and investigated the behavior of chemical species within them. The cavities of self-assembled coordination cages constitute yet another family of synthetic confined environments. Within these cavities, many unique chemistries have been observed, including tunable fluorescent properties of the trapped species^8, 9^, stabilization of otherwise reactive species^10^ (such as white phosphorus^11^, the cyclic trimers of siloxanes^12^, and free-radical initiators^13^), and efficient catalysis of Diels–Alder cycloadditions^14, 15^, and terpene cyclizations^16^, among other reactions^17–19^. In contrast, the properties of photoswitchable molecules in the cavities of self-assembled cages have remained largely unexplored.

Photoisomerization of molecular switches in confined spaces can be a challenging task. As most of these switches (including azobenzenes, stilbenes, and spiropyrans) isomerize, they undergo large conformational changes^20–23^ and hence require some conformational freedom, which is typically not available under confinement. For example, it has long been recognized that placing azobenzene within densely packed self-assembled monolayers^24^ or inside coordination cages^25^ renders it non-switchable. In another system, spiropyran residing in the cavity of a dimeric capsule held together by hydrogen bonds could be successfully isomerized, but at the expense of the disintegration of the supramolecular host^26^. Similarly, photoisomerizing azobenzenes bound inside different self-assembled capsules^27, 28^ led to the expulsion of the guests from the respective hosts.

In nature, the requirement of conformational freedom is solved by encapsulating photoswitchable molecules within hosts that are flexible. For example, opsin proteins can adapt their structure to the conformation of retinal, enabling a highly efficient *E*/*Z* photoisomerization of the encapsulated guest^29^. In contrast, with a few exceptions^30–32^, man-made cages are structurally robust, and therefore not well suited to guide photoisomerization reactions.

Here we show that a flexible, water-soluble coordination cage is capable of encapsulating—and solubilizing in water—several light-responsive spiropyran molecular switches. The encapsulation is accompanied by isomerization of the guest to its otherwise unstable form. X-ray crystal structure of the inclusion complex reveals that upon binding of the guest, the cage undergoes a significant structural deformation. Owing to its flexibility, the cage provides an environment suitable for the efficient, reversible photoisomerization of the bound spiropyrans. Taking advantage of these findings, we develop two time-sensitive information storage media: a paper, on which writing can be performed using water as the ink, and a gel, which can be reversibly patterned using light.

## Results

### Computer simulations

**Fig. 1:**
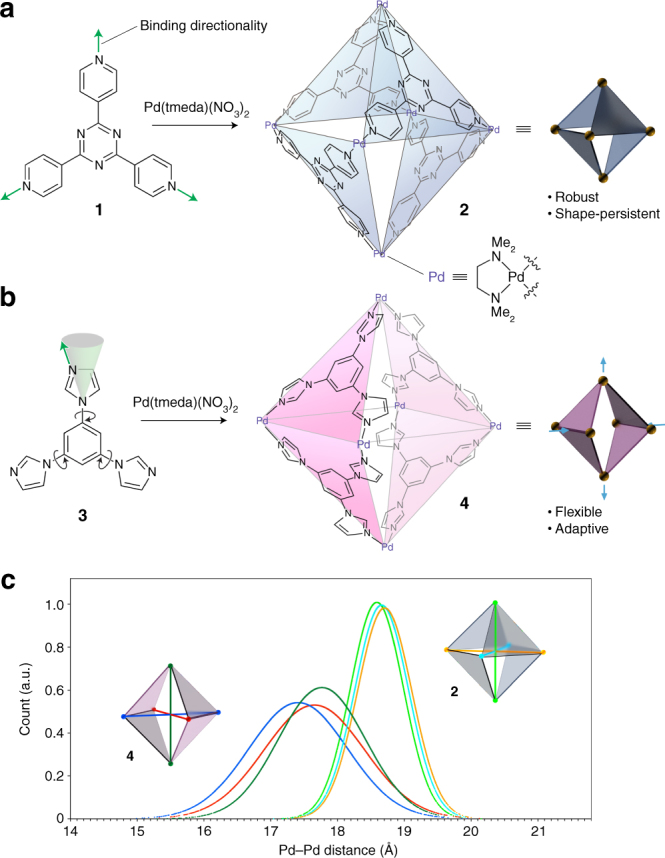
Tripyridine- and triimidazole-based cages and their mechanical properties. **a** Synthesis of an octahedral cage **2** from tripyridine ligand **1** and a *cis*-blocked Pd acceptor^33^. **b** Synthesis of octahedral cage **4** from the same Pd precursor and triimidazole ligand^34^ **3**. **c** Quantifying the flexibility of cages **2** and **4**: calculated distributions of Pd–Pd distances in **2** and **4** at room temperature

We focused our studies on Fujita’s cage **2** (Fig. 1a)^33^ and Mukherjee’s cage **4** (Fig. 1b)^34^. Cages **2** and **4** can be obtained in quantitative yields by reacting *cis*-blocked Pd acceptor (Pd(tmeda)(NO_3_)_2_, where tmeda = tetramethylethylenediamine) with ligands **1** and **3**, respectively. A subtle change in the structure of the ligand (replacing three pyridine groups with imidazoles) results in a major difference in the topology of the resulting cages: whereas in **2**, the ligands occupy alternating faces of the octahedron, cage **4** features a horizontal symmetry plane (Fig. 1). We hypothesized that this change in cage architecture would give rise to pronounced differences in the flexibility of the two cages.

In order to test this hypothesis and to better understand the mechanical properties of cages **2** and **4**, we performed atomistic molecular dynamics (MD) simulations of both cages in explicit water (see Methods for details). Supplementary Movies 1 and 2 show the dynamics of **2** and **4**, respectively, at room temperature. We analyzed the dynamics in terms of the distances between the diagonal Pd centers (i.e., Pd^2+^ ions residing at the opposite sides of the cages; i.e., light-green, cyan, and orange in cage **2**, and dark-green, red, and blue in **4**; Fig. 1c). The resulting normal distributions of Pd–Pd distances can act as indicators of the conformational flexibility of the cages. We found that under otherwise identical conditions, the Gaussian thermal distributions for the Pd–Pd distances in cage **2** were much narrower than in **4**, with standard deviations of *σ* = 0.43 Å and 0.86 Å for **2** and **4**, respectively (Fig. 1c). The same trend was observed at elevated temperatures (Supplementary Fig. 7), where all peaks became broader. Overall, these studies confirmed that **4** is significantly more flexible than **2**, suggesting that it can undergo relatively large conformational changes that allow for encapsulating differently shaped guest molecules, such as two structurally different isomers of the same molecular switch.

### Encapsulation of spiropyrans

**Fig. 2:**
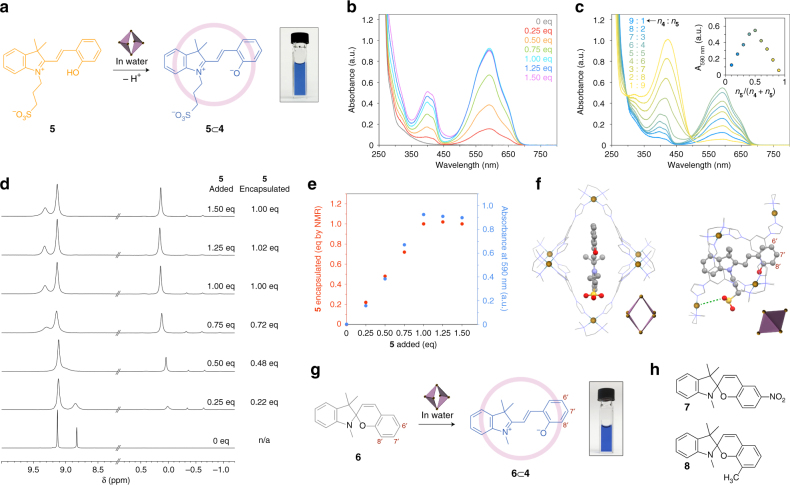
Encapsulation of a spiropyrans within a self-assembled cage. **a** Encapsulation-induced deprotonation of **5**. **b** Changes in the UV/Vis absorption spectra of **4** upon titrating with **5** (all spectra were recorded once equilibrium was reached). **c** UV/Vis absorption spectra of an aqueous solution containing different molar ratios of **4**/**5** at the same total concentration of the two species. The resulting Job’s plot reveals that the **4**–**5** complex has a 1:1 stoichiometry. **d** Partial ^1^H NMR spectra (400 MHz, D_2_O) of **4** in the presence of increasing amounts of **5**. The downfield-shifted signals correspond to **4**’s imidazole protons. The upfield-shifted signals are due to the methyl groups of encapsulated **5** (eq=equivalent). **e** Following the binding of guest **5** by cage **4** using UV/Vis absorption spectroscopy and NMR (replotted from **b** and **d**). **f** Two views of the X-ray crystal structure **5**⊂**4** (the cartoons show the orientation of the cage). The hydrogen atoms were removed for clarity. **g** The** 4**-induced ring-opening isomerization of spiropyran **6**. **h** Structural formulas of spiropyrans **7** and **8** investigated as potential guests

Encouraged by these results, we hypothesized that the flexible cavity of **4** might provide an environment suitable for the reversible isomerization of molecular switches whose isomerization is associated with large structural changes. We first considered compound **5** (Fig. 2a)—a protonated-merocyanine (MCH^+^) form of a spiropyran appended with a sulfonic acid group—which can undergo a reversible ring-closing reaction upon exposure to blue light^35^. Interestingly, when an aqueous solution of cage **4** was treated with solid **5**, an intense blue (*λ*_max_ ≈ 592 nm) color appeared instantly (Fig. 2a). This band characteristic of the merocyanine (MC) isomer of the switch residing in a nonpolar environment. No color changes were observed in control experiments, in which we mixed **5** with free components of the cage (i.e., ligand **3** and Pd(tmeda)(NO_3_)_2_).

The formation of the MC form (i.e., **5**_MC_) was surprising given the absence of an electron-withdrawing substituent at the *para* position^21, 36^ with respect to O^–^. To better understand the interactions between **5** and **4**, we recorded a series of ultraviolet/visible (UV/Vis) absorption spectra while titrating the cage with **5**. The absorption intensity at 592 nm increased with increasing concentrations of **5** in the system, but only until 1.0 molar equivalent of **5** was added (Fig. 2b, c), suggesting that each molecule of **4** is only capable of transforming one molecule of **5**_MCH+_ into **5**_MC_. Next, we recorded a series of proton nuclear magnetic resonance (^1^H NMR) spectra of the cage in the presence of increasing amounts of **5** (Fig. 2d). The upfield-shifted signals at ~ 0 ppm can be assigned to the CH_3_ protons of **5** bound in the hydrophobic cavity of **4**. Integrating these signals with respect to those due to the imidazole protons of **4** (~ 9.3–8.8 ppm) showed that each cage could encapsulate one molecule of **5**, giving rise to inclusion complex **5**⊂**4**, in agreement with the UV/Vis results (Fig. 2e). The association constant of the complex could be roughly estimated by UV/Vis titration as *K*_assoc_ > 10^7^ M^−1^ (see Supplementary Note 9).

Single crystals of **5**⊂**4** were obtained by slow evaporation of water from an aqueous solution of the complex. The X-ray crystal structure revealed the presence of the open-ring isomer of switch **5** bound tightly in the cavity of host **4**, with which it interacts by π–π stacking interactions (Fig. 2f). In addition, the binding is likely facilitated by the release of high-energy water molecules from the cavity of **4**, as reported previously for other host–guest complexes^37, 38^ (see also ref. ^39^ and the discussion in Supplementary Note 10). Notably, upon encapsulating **5**, cage **4** underwent a significant distortion, whereby the distance between the two apical Pd^2+^ ions increased from 16.9 Å (for guest-free **4**) to 18.3 Å; (see also Supplementary Note 11). This deformation was enabled by varying the C-C-N-C dihedral angle (see Fig. 1b) rather than distorting the square planar geometry of the PdN_4_ moiety. This result emphasizes the importance of the structural flexibility of **4** for binding guest molecules. Indeed, adding **5** to the aqueous solution of the more shape-persistent cage **2** did not result in any color changes.

A more striking manifestation of the high affinity of cage **4** towards the MC form of spiropyrans was found for the simplest spiropyran **6** (Fig. 2g). Although compound **6** exhibits extremely low solubility in pure water, it could readily be solubilized in an aqueous solution of **4** and the resulting solution had a deep-blue color akin to that of **5**⊂**4** (Fig. 2g). The identity of the **6**⊂**4** complex was confirmed using a combination of NMR methods (Supplementary Figs. 25-32), which showed that cage **4** reverses the relative stability of the closed-ring and the open-ring isomer of **6**, stabilizing the otherwise unstable form **6**_MC_. Unlike **5**_MC_⊂**4**, however, complex **6**_MC_⊂**4** did not form in a quantitative yield—comparing the intensity of **4**s protons with those of **6** revealed that only ~50% of the cages become filled during stirring with an excess of solid **6**. This can be explained by the very low concentration of the saturated solution of **6** in water, which implies that the equilibrium concentration of the unoccupied cage, [**4**], remains relatively high even for a high value of *K*_assoc_ (*K*_assoc_ = [**6**⊂**4**]/[**4**]·[**6**]). Overall, these results indicate that the energy penalty associated with distorting the cage can be overcome by the favorable host–guest interaction energy, and that by preferentially binding an otherwise unstable isomer of the guest, the cage can render this isomer the stable one (as has also been observed by Fujita and co-workers^40^ for free vs. encapsulated phthalein dyes).

Interestingly, cage **4** showed no affinity to the *para*-nitro derivative of **6**, i.e., the most commonly used^41–48^ spiropyran (**7** in Fig. 2h). This was surprising given the increased propensity of **7** to exist in the open-ring form. However, inspection of the X-ray structure of **5**⊂**4** revealed that substituents at the 6′ position would collide with the cage’s backbone (Fig. 2f), which likely explains why complex **7**⊂**4** does not form. At the same time, this reasoning suggests that substitutions at the 7′ and the 8′ positions would be tolerated. To verify this hypothesis, we synthesized compound **8** (Fig. 2h), which has an extra methyl group at the 8′ position. We were pleased to find that similar to **5** and **6**, spiropyran **8** could be solubilized in water in the presence of cage **4**. The solubility was proportional to the amount of the dissolved cage (which has a very high, > 0.3 g/mL, solubility in water) and we consistently saw that stirring with an excess of **8** resulted in filling of ~ 70% of **4** (Supplementary Fig. 34).

Despite the tendency of **4** to stabilize the open form of all of the encapsulated spiropyrans (**5**, **6**, and **8**), we found some notable differences between **5**⊂**4** on one hand, and complexes **6**⊂**4** and **8**⊂**4** on the other. First, UV/Vis absorption spectra of **5**⊂**4** showed an unusually fine structure of MC absorption bands (Supplementary Fig. 41a). In contrast, both **6**⊂**4** and **8**⊂**4** exhibited spectra typical of merocyanine, with a broad band at ~ 580 nm and an intense absorption in the blue part of the spectrum. Well-defined features in the absorption spectra are a manifestation of increased rigidity of the molecule (as a result of, e.g., cooling^49^ or intramolecular bridging^50^). In our case, the decreased conformational freedom of **5** might be due to a tight binding in the cavity of **4**. Second, **5** bound within **4** showed very broad peaks in the ^1^H NMR spectrum (Supplementary Fig. 14), which, again, is a manifestation of restricted molecular motion (similar line broadening is well known for molecules confined to the surfaces of metallic nanoparticles^51–54^). Contrary to **5**, encapsulated **6** and **8** both showed a set of sharp, well-defined signals (Supplementary Figs. 25 and 34, respectively). Third, we found that upon excitation with blue light, **5**⊂**4** exhibited green fluorescence (Supplementary Note 14), which is absent unless the closed-ring isomer is constrained^55^—indeed, no green emission was observed for **6**⊂**4** or **8**⊂**4**. Fourth, despite extensive efforts, we did not succeed in obtaining single crystals of either **6**⊂**4** or **8**⊂**4** of a quality suitable for X-ray diffraction studies, which can be correlated to the relatively large conformational freedom of **6** and **8** within **4**. In sharp contrast, **5**⊂**4** afforded high-quality single crystals without difficulties. We hypothesize that this difference in stabilization of **5** vs. **6** and **8** can be attributed to the electrostatic interactions between **5**’s SO_3_^–^ group and one of **4**s Pd^2+^ centers (Fig. 2f, dashed green line).

### Concentration dependence of the optical properties of encapsulated spiropyrans

**Fig. 3:**
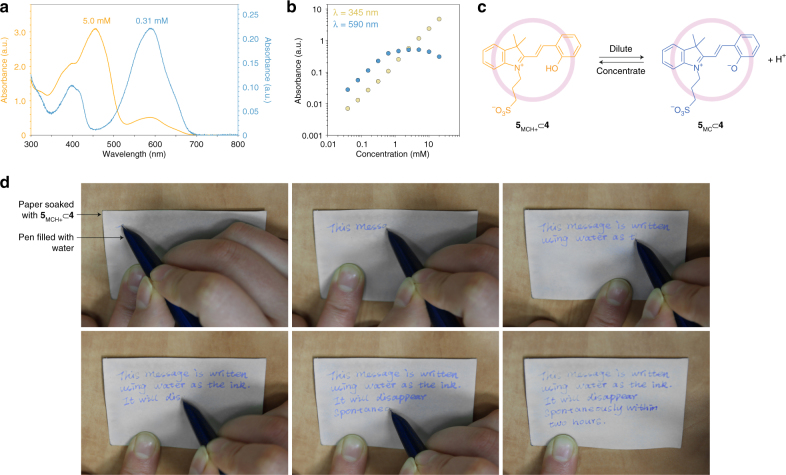
Concentration-dependent optical properties of encapsulated spiropyran. **a** UV/Vis absorption spectra of a concentrated (yellow; 5.0 mM) and diluted (blue; 0.31 mM) solution of **5**⊂**4** in water. **b** Concentration dependence of the absorbance of **5**⊂**4** at 345 nm (where all isomers of **5** absorb equally) and at 592 nm (where only the MC isomer absorbs). **c** Dilution-induced deprotonation of the MCH^+^ isomer of **5** encapsulated within **4**. **d** Writing in a **5**⊂**4**-soaked paper with a pen filled with pure water. Local delivery of water induces the deprotonation of **4**-encapsulated MCH^+^ and consequently the appearance of a deep-blue color. The created message gradually disappears as water evaporates

Aqueous solutions used to grow crystals of **5**⊂**4** exhibited unexpected color changes during water evaporation: instead of gradually increasing, the color intensity decreased, and the solution turned from blue to yellow (the crystals of **5**⊂**4** were pale yellow). To quantify these changes, we recorded a series of UV/Vis spectra of **5**⊂**4** at concentrations spanning three orders of magnitude (Supplementary Fig. 44). Representative spectra of the same solution at two different concentrations are shown in Fig. 3a. Upon concentrating the solution from ~0.3 mM to ~5 mM, the absorption band at ~592 nm (due to the MC form) decreased at the expense of an intense peak at ~460 nm. This change in color was reversible and by diluting the solution, the original spectrum could be restored. Figure 3b shows the dependence of absorbance at two different wavelengths as a function of the solution concentration. Absorbance at 345 nm is proportional to the total amount of **5**⊂**4** (i.e., all isomers of **5**) and it shows a linear dependence on concentration, following the Lambert–Beer law. However, the absorbance at 592 nm—a region where only the MC isomer absorbs—decreased at high concentrations. This decrease is at the expense of MCH^+^, which is the species that absorbs strongly at ~460 nm. These color changes are a manifestation of the well-known property^56^ of weak acids (here, MCH^+^) to dissociate to a greater extent in dilute solutions (Fig. 3c). Interestingly, no color changes were observed upon diluting a solution of **5** in the absence of **4**, emphasizing the unique role of the cage in stabilizing the MC isomer of **5**.

Based on these findings, we anticipated that by diluting the **5**_MCH+_ species (and by generating the deep-colored **5**_MC_) in a spatially controlled fashion, we might be able to create high-contrast patterns using water as the stimulus.^57^ To test this hypothesis, we soaked a 7 × 5 cm piece of cellulose paper in a dilute (i.e., blue) aqueous solution of **5**_MC_⊂**4** and then allowed water to evaporate, resulting in an off-white paper (Fig. 3d, top left). We then filled the ink reservoir of a fountain pen with pure water, and used it to write on the **5**_MCH+_⊂**4**-doped paper, as shown in Fig. 3d. The resulting text disappeared spontaneously within ca. 2 h owing to water evaporation, and the process could be repeated. This system has two attractive features: first, the “ink” is permanently stored in the paper and it does not need to be delivered from the pen; second, the paper can be used for many write–erase cycles.

### Photoisomerization of encapsulated spiropyrans

**Fig. 4:**
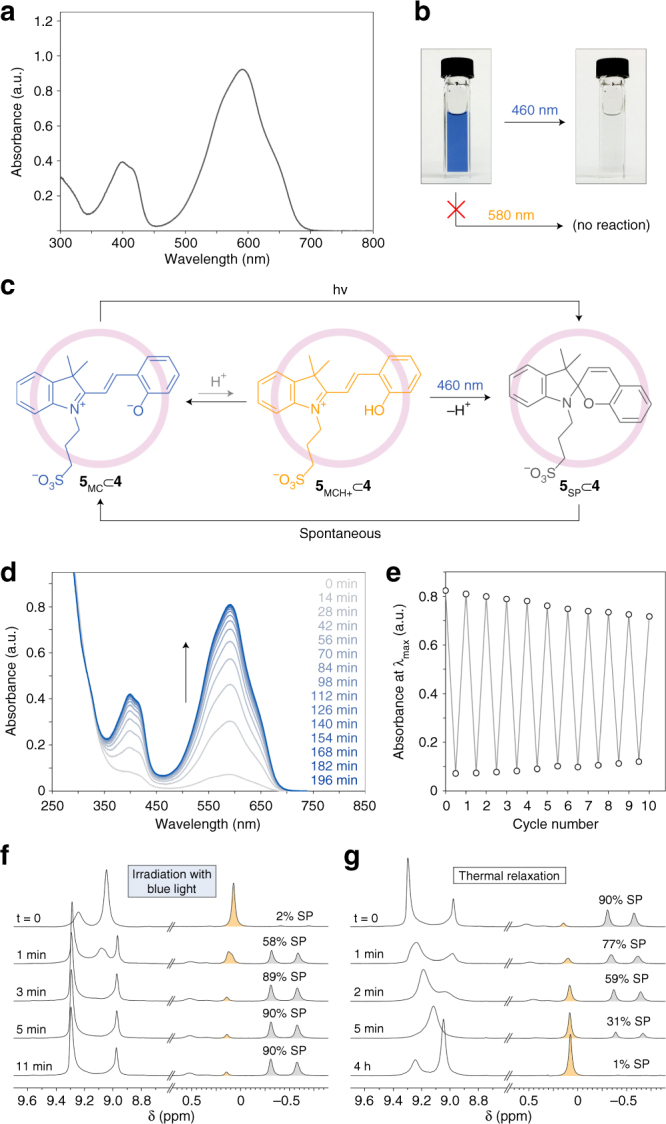
Photochromism of spiropyran in the cavity of a self-assembled cage. **a** UV/Vis absorption spectrum of a dilute (0.5 mM) aqueous solution of **5**⊂**4** after subtracting the background due to cage **4**. **b** Efficient decoloration of the blue solution of **5**⊂**4** using blue light. **c** Proposed mechanism of the light-induced decoloration of **5**⊂**4**. **d** A series of UV/Vis spectra of **5**⊂**4** accompanying thermal equilibration in the dark (here, *t* = 0 min corresponds to a solution pre-exposed to blue light). **e** Reversibility of the light-induced decoloration of **5**⊂**4**. **f** Partial ^1^H NMR spectra (400 MHz, D_2_O) of **5**⊂**4** during in situ irradiation with blue light for increasing periods of time. The downfield-shifted signals are due to **4**’s imidazole protons (note the pronounced changes accompanying the isomerization of the bound guest). The upfield-shifted signals are due to the methyl groups of encapsulated **5**. The signals highlighted in yellow are due to the MCH^+^ isomer and those in gray are due to SP. **g** Changes in the ^1^H NMR spectra of **5**⊂**4** pre-irradiated with blue light upon relaxation in the dark

Next, we considered photoisomerizing the cavity-bound merocyanine **5**_MC_. Figure 4a shows the absorption spectrum of a dilute solution of encapsulated **5**_MC_. Interestingly, irradiating this solution near the absorption maximum (we used a yellow (580 nm) light-emitting diode (LED)) did not result in any spectral changes. This is in sharp contrast to the behavior of free **5**_MC_ in an aqueous solution (*λ*_max_ = 530 nm; obtained by treating **5**_MCH+_ with 1 eq of NaOH), which upon exposure to a green (520 nm) LED underwent a ring-closing isomerization. These results can be explained by the efficient stabilization of **5**_MC_ by the cage. However, after testing a variety of light sources, we found that the solution of **5**_MC_⊂**4** could be turned transparent upon a brief (30 s) exposure to low-intensity blue (460 nm) light (Fig. 4b). This finding was surprising given that 460 nm corresponds to the minimum absorption of **5**_MC_⊂**4** (see Fig. 4a). We note, however, that the MCH^+^ form absorbs strongly in this region (Fig. 3a, orange spectrum). This leads us to propose the following mechanism underlying the photoisomerization of the cavity-bound **5**_MC_. In an aqueous solution, the MC and MCH^+^ forms can coexist, **5**_MC_⊂**4** + H_2_O ⇄** 5**_MCH+_⊂**4 **+ HO^–^. In dilute solutions, the equilibrium is strongly shifted to the left and the MCH^+^ species is undetectable by UV/Vis spectroscopy. However, the minute amounts of MCH^+^ can efficiently absorb blue light and undergo photoisomerization to the SP form^21^. The equilibrium is gradually shifted to the right until all **5**_MC_⊂**4** is converted to the colorless **5**_SP_⊂**4** (Fig. 4c). To confirm this mechanism, we performed the same reaction in several buffer solutions having different pH values, and found that the rate of decoloration was inversely proportional to the solution’s pH (Supplementary Fig. 55).

The reaction was reversible: once irradiation was discontinued, the solution regained its original blue color (Fig. 4d). Kinetic analysis of the coloration reaction performed by monitoring the change in absorbance at 592 nm revealed that the reaction obeyed first-order kinetics, with a half-life, *τ*_1/2_ = 26.3 min. The system was reversible; however, some fatigue was observed (Fig. 4e), which could be attributed to the previously reported^26, 58^ slow hydrolysis of spiropyrans in water. To gain additional insight into the reversible isomerization reaction, we recorded a series of ^1^H NMR spectra of **5**⊂**4** in D_2_O while irradiating the sample with a 460 nm LED in situ through an optical fiber. Notably, even at the high concentrations (~5 mM) typical for NMR experiments, as much as 90% **5**⊂**4** could be converted to the closed-ring isomer (Fig. 4f). More strikingly, the photoisomerization reaction was accompanied by pronounced changes in the NMR signals of the cage (Fig. 4f, g; see also Supplementary Fig. 48), which, again, highlights the importance of cage’s flexibility for efficient photoswitching of the encapsulated molecules.

Interestingly, the **5**⊂**4** complex remained photochromic even in the solid state. Supplementary Fig. 56b shows the absorption spectra of a thin film of solid **5**⊂**4** on a glass slide, where the ~445 nm band resulting from the MCH^+^ form can be quenched within 1 min of irradiation with blue light. The spontaneous back-isomerization took ~6 h to complete, and, unlike the solution reaction, it did not follow the first-order kinetics, suggesting the involvement of protons in the ring-opening reaction taking place in the solid state.

Finally, we were interested in how replacing **5** with **6** or **8** inside the cage affects the kinetics of the spontaneous back-isomerization (i.e., ring-opening reaction). Interestingly, we found that following exposure to blue light, both **6**_SP_⊂**4** and **8**_SP_⊂**4** back-isomerized much faster compared with **5**_SP_⊂**4** (Supplementary Fig. 46, b and c). The kinetics of both reactions were fitted to the first-order equation, with *τ*_1/2_ = 5.0 min and 2.5 min for **6**⊂**4** and **8**⊂**4**, respectively. The unusually slow ring-opening reaction of **5**_SP_⊂**4** could once again be attributed to the restricted molecular motion of the encapsulated **5** (vide supra).

**Fig. 5:**
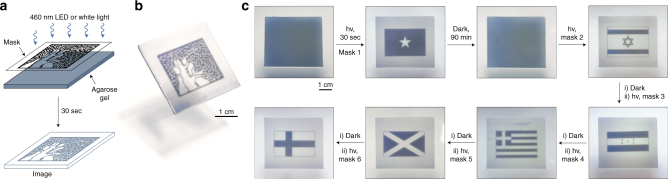
Writing self-erasing images in a photoresponsive gel soaked with an inclusion complex. **a**, Creating an image in a **5**⊂**4**-soaked agarose gel by exposing it to light through a mask (here, featuring the logo of the Weizmann Institute of Science) and **b**, a photograph of the resulting image. **c**, A series of images (flags of Somalia, Israel, Honduras, Greece, Scotland, and Finland) created consecutively in the same piece of agarose gel

Similar to the dilution-controlled color changes described above, the reversible, light-induced decoloration reaction could be used to develop a novel time-sensitive information storage medium. To this end, we prepared thin agarose gels soaked with dilute (i.e., blue) solutions of **5**⊂**4**, and verified that both the light-induced ring-closing reaction and the spontaneous ring opening proceeded with kinetics identical to those in bulk solution. By exposing these gels to low-intensity blue light locally (through a mask), high-contrast images could be created (Fig. 5a, b). These images persisted for ca. 90 min, during which the spontaneous coloration in the pre-irradiated regions was completed. As Fig. 5c shows, multiple images could be created consecutively in the same piece of gel. To control the images’ lifetime, we also fabricated agarose gels soaked with **6**⊂**4** or **8**⊂**4**. We found that images could readily be created in these gels as well, but, as expected, they disappeared considerably faster (within ca. 10 min).

## Discussion

We investigated the binding and photoinduced structural transformations of a model light-responsive molecular switch, spiropyran, within a flexible coordination cage. We showed that the encapsulation of different spiropyrans in a hydrophobic cavity of the cage was accompanied by spontaneous switching to an otherwise unstable, colored isomer, highlighting the cage’s capacity to revert the relative stability of different isomers of the same molecule.^40^ The encapsulated spiropyrans remained photoswitchable and could be converted to the colorless form upon irradiation with blue light. These findings constitute an unprecedented case of negative photochromism of spiropyran in a hydrophobic environment (here, the cavity of the cage). We believe that our results might open several interesting research directions. First, it will be of interest to expand the cage’s cavity size, which can be achieved by separating the benzene and the imidazole rings in **3** by phenylene or −C≡C− linkers. We envision that the expanded cages will be able to accommodate larger numbers of the same guest molecules—in this context, we are particularly interested in trapping discrete oligomers of photoswitchable molecules with the goal of systematically investigating cooperative switching processes^59–61^. Second, we are currently developing a metal-free version of flexible cages, which will be advantageous for large-scale applications, such as in reversible information storage media. Third, we believe that the entrapment of nonpolar catalytic moieties (both metal-based catalysts and organocatalysts) is an attractive approach towards constructing catalytic systems operating in water. Here, we wish to point out that the high flexibility and adaptability of the polypeptide chains surrounding the enzymatic active sites are often critical for efficient catalysis in biological systems. Finally, our results indicate that light energy can be used (via the noncovalently bound molecular switch) to reversibly change the shape of a non-photoresponsive molecular system (here, a self-assembled cage), thus paving the way towards the development of novel molecular machines^62–65^.

## Methods

### Synthesis of cage 4

The cage was synthesized according to a modified literature procedure^34^. A solution of *cis*-[(tmeda)Pd(NO_3_)_2_] (200 mg, 0.577 mmol) in water (25 mL) was added slowly to 1,3,5-tris(1-imidazolyl)benzene (106 mg, 0.384 mmol) and the resulting reaction mixture was stirred for 24 h at room temperature. Then, the mixture was centrifuged to remove any insoluble materials. The supernatant was collected and it was concentrated under reduced pressure. Cage **4** in the crystalline form was obtained by slow vapor diffusion of acetone. Isolated yield: 95%.

### General procedure for encapsulation of guests inside cage 4

Guest **5** (5.4 mg; 0.014 mmol), **6** (3.9 mg; 0.014 mmol), or **8** (4.1 mg; 0.014 mmol) was suspended in an aqueous (H_2_O or D_2_O) solution of cage **4** (15 mg = 0.005 mmol in 1 mL of water (we found no evidence of the encapsulation of spiropyran **7**)). The mixture was stirred for 24 h at room temperature. Encapsulation entailed solubilization of the guest in water. The resulting milky suspension was filtered through glass wool and centrifuged several times at 5000 rpm to yield a clear solution. The yield of encapsulation was determined by ^1^H NMR spectroscopy. We verified that stirring times longer than 24 h did not increase the encapsulation yield. We also found that the encapsulation yield was not increased by ultrasonication or heating to 60 °C. The same procedure was used in attempting to encapsulate the above molecules **5**–**8** inside cage **3**.

### Computational studies

Molecular force field parameters for the studied systems were obtained as follows. We used the CHARMM general force field^66, 67^ to describe all the bonds, angles, dihedrals, and nonbonding parameters of the cage except those around Pd atoms. Each cage was accompanied by 12 NO_3_^–^ ions for overall electroneutrality. The parameters for nitrate ions were taken from the literature^68^. To retain the planar structure of the nitrate ions, we imposed an improper angle with a force constant of 90 kcal/mol rad^2^ and an angle of 180°. For the Pd-N coordination bonds, average distances from the experimentally obtained X-ray were used with a force constant of 600 kcal/mol Å^2^. For cage **2**, the Pd-N_**1**_ (where N_**1**_ denotes the donating nitrogen atom of ligand **1**) distance was 2.03 Å and the Pd-N_tmeda_ distance (where N_tmeda_ denotes tmeda’s nitrogen atom) was 2.05 Å;. For cage **4**, the Pd-N_**3**_ distance was 2.03 Å (N_**3**_ denotes the donating nitrogen atom of ligand **3**) and the Pd-N_tmeda_ distance was 2.05 Å. Nonbonding interactions were calculated using a cutoff distance of 10 Å and long-range electrostatic interactions were calculated using the PME method^69^ in the presence of periodic boundary condition. In each system, TIP3P molecules were used in a cubical water box with a side length of 60 Å. Atomistic MD simulations were performed with NAMD using an NPT ensemble^70^. We used Langevin dynamics with a damping constant of 1 ps^−1^ and a time step of 2 fs. The systems were simulated at temperatures of 298 K, 400 K, and 450 K. To overcome the limitations of the physical model, the planar symmetry of the PdN_4_ moieties was restrained during the minimization and warming steps. In the equilibration step, each PdN_4_ moiety was restrained to preserve its planar geometry by setting the root-mean-square deviation to be less than 0.1 Å and the force constant of 4,000 kcal/mol, using the collective variables (colvar) module in NAMD.

### Fabrication of rewritable paper

A 7 × 5 cm piece of regular paper was placed in a glass Petri dish containing a solution of 37 mg (0.010 mmol) of **5**⊂**4** in 4 mL of water. After 12 h, the blue-colored paper was removed from the Petri dish and dried overnight in a vacuum desiccator: it became pale yellow (near colorless). The ink reservoir of a fountain pen was emptied and washed with pure water until all the dye was washed away. The pen’s ink reservoir was filled with distilled water and then used to write on the “rewritable paper”.

### Fabrication of the photoresponsive agarose gel

One gram of agarose (CAS # 9012-36-6, biotechnology grade, Amresco product # 0710) was added to an Erlenmeyer flask containing 50 mL of distilled water. The mixture was heated in a microwave oven until water started to boil. After an additional 3 min, heating was discontinued and the flask was removed from the oven. The resulting colorless, homogeneous solution was poured while hot between two glass slides separated by a 1 mm spacer. After having been cooled to room temperature, a 5 × 4 × 0.1 cm piece of solidified agarose gel was placed in a Petri dish containing a solution of 26.9 mg (0.008 mmol) of **5**⊂**4** in 7.0 mL of distilled water for 30 min. Finally, the gel was briefly rinsed with water.

### Data availability

The X-ray crystallographic coordinates for structures reported in this article have been deposited at the Cambridge Crystallographic Data Centre (CCDC), under deposition number CCDC 1551434 and 1551437 for compound **4** and **5**⊂**4** respectively. These data can be obtained free of charge from the Cambridge Crystallographic Data Centre via www.ccdc.cam.ac.uk/data_request/cif. All relevant data supporting the findings of this study are available from the corresponding authors on request.

## Electronic supplementary material

Supplementary Information

Description of Additional Supplementary Information

Supplementary Movie 1

Supplementary Movie 2
