# Publisher Correction: Reversible chromism of spiropyran in the cavity of a flexible coordination cage

**DOI:** 10.1038/s41467-018-03701-2

**Published:** 2018-04-12

**Authors:** Dipak Samanta, Daria Galaktionova, Julius Gemen, Linda J. W. Shimon, Yael Diskin-Posner, Liat Avram, Petr Král, Rafal Klajn

**Affiliations:** 1 Department of Organic Chemistry, Weizmann Institute of Science, 76100 Rehovot, Israel; 2 Department of Chemistry, University of Illinois at Chicago, 60607 Chicago, IL USA; 3 Department of Chemical Research Support, Weizmann Institute of Science, 76100 Rehovot, Israel; 4 Department of Physics, University of Illinois at Chicago, 60607 Chicago, IL USA; 5 Department of Biopharmaceutical Sciences, University of Illinois at Chicago, 60607 Chicago, IL USA

Correction to: *Nature Communications* **9**:641; 10.1038/s41467-017-02715-6; Published online 13 February 2018

The original version of this Article contained an error in Fig. 4c, in which the right-most chemical structure included an ‘N^+^’ rather than an ‘N’. The correct version of Fig. 4c is:

which replaces the previous incorrect version:

This has been corrected in both the PDF and HTML versions of the Article.

