# Supplementary material for: Reversible chromism of spiropyran in the cavity of a flexible coordination cage
Source: Nat Commun. 2018 Feb 13;9:641. doi: 10.1038/s41467-017-02715-6 (PMC5811438; doi:10.1038/s41467-017-02715-6)
Supplement: Supplementary file 1 — Supplementary Information [file 41467_2017_2715_MOESM1_ESM.pdf]

Supplementary Note 1 | General information.

All commercial chemicals were used as received unless stated otherwise. Solvents were dried according to standard procedures (dichloromethane was dried over 4 Å molecular sieves). Cages **2** and **4** and guests **5-7** were prepared following the literature procedures. NMR spectra were recorded on a Bruker Avance III 400 MHz, Bruker Avance III HD 500 MHz, or a Bruker Avance III 800 MHz spectrometer. Chemical shifts (δ) are given in ppm relative to residual protio solvent resonances (2.51 ppm for $(CD_3)_2SO$, 4.79 ppm for D_2O, 7.26 ppm for $CDCl_3$, and 5.32 ppm for CD_2Cl_2). ^{1}H-DOSY NMR measurements were performed on a Bruker Avance III HD 500 MHz spectrometer or a Bruker Avance III 800 MHz spectrometer with temperature and gradient calibration prior to the measurements. The diffusion coefficient of the solvent was used as a calibration standard. A constant temperature of 298 K was maintained during the measurements. Electrospray ionization mass spectrometry (ESI-MS) experiments were carried out in a Bruker Daltonics (Esquire 300 Plus ESI model) using standard spectroscopic-grade methanol. UV/Vis absorption spectra were recorded with a Shimadzu UV-2700 or a UV-3600 spectrophotometer. Emission spectra were recorded with a Shimadzu spectrofluorophotometer RF-5301 PC. For photoirradiation experiments, we used a Prizmatix mic-LED 460 nm light-emitting diode (LED) as a blue light source, a Prizmatix 520 nm Ultra High Power (UHP) Mic-LED LED (collimated LED power of 900 mW) as a green light source, and a Prizmatix Ultra High Power White LED equipped with a bandpass filter of 580±25 nm as a yellow light source. For following the photoisomerization reactions in-situ using NMR, the LEDs were equipped with a high numerical aperture polymer optical fiber (POF) (diameter 1 mm, length 5 m). Thermogravimetric analysis (TGA) was performed on a Q600 SDT instrument (TA Instruments). The heating rate was 10 °C·min^{-1}. The sample was measured under a nitrogen flow of 100 mL·min^{-1}. pH measurements were performed using a SevenCompact S210 pH meter (Mettler Toledo). Elemental analysis was carried out on a FlashEA 1112 CHN analyzer (Thermo Fischer Scientific).

Supplementary Note 2 | Synthesis and characterization of cage **2**.

Cage **2** was synthesized based on a literature procedure[1]. To a solution of *cis*-[(tmeda)Pd(NO_3)$_2$] (tmeda = tetramethylethylenediamine) (200 mg, 0.577 mmol) in water (25 mL) was added 2,4,6-tri(pyridin-4-yl)-1,3,5-triazine (120 mg, 0.384 mmol) and the resulting mixture was stirred for 24 h at 50 °C. Then, the reaction mixture was centrifuged and concentrated under reduced pressure to yield an off-white solid. Isolated yield: 97%.

^{1}H NMR (500 MHz, D_2O): δ = 9.33 (d, ^3J = 6.5 Hz, 24H), 8.81 (d, ^3J = 6.5 Hz, 24H), 3.21 (s, 24H), 2.81 (s, 72H). **^{13}C NMR** (125 MHz, D_2O): δ = 169.13, 151.75, 145.55, 125.99, 62.76, 50.35.

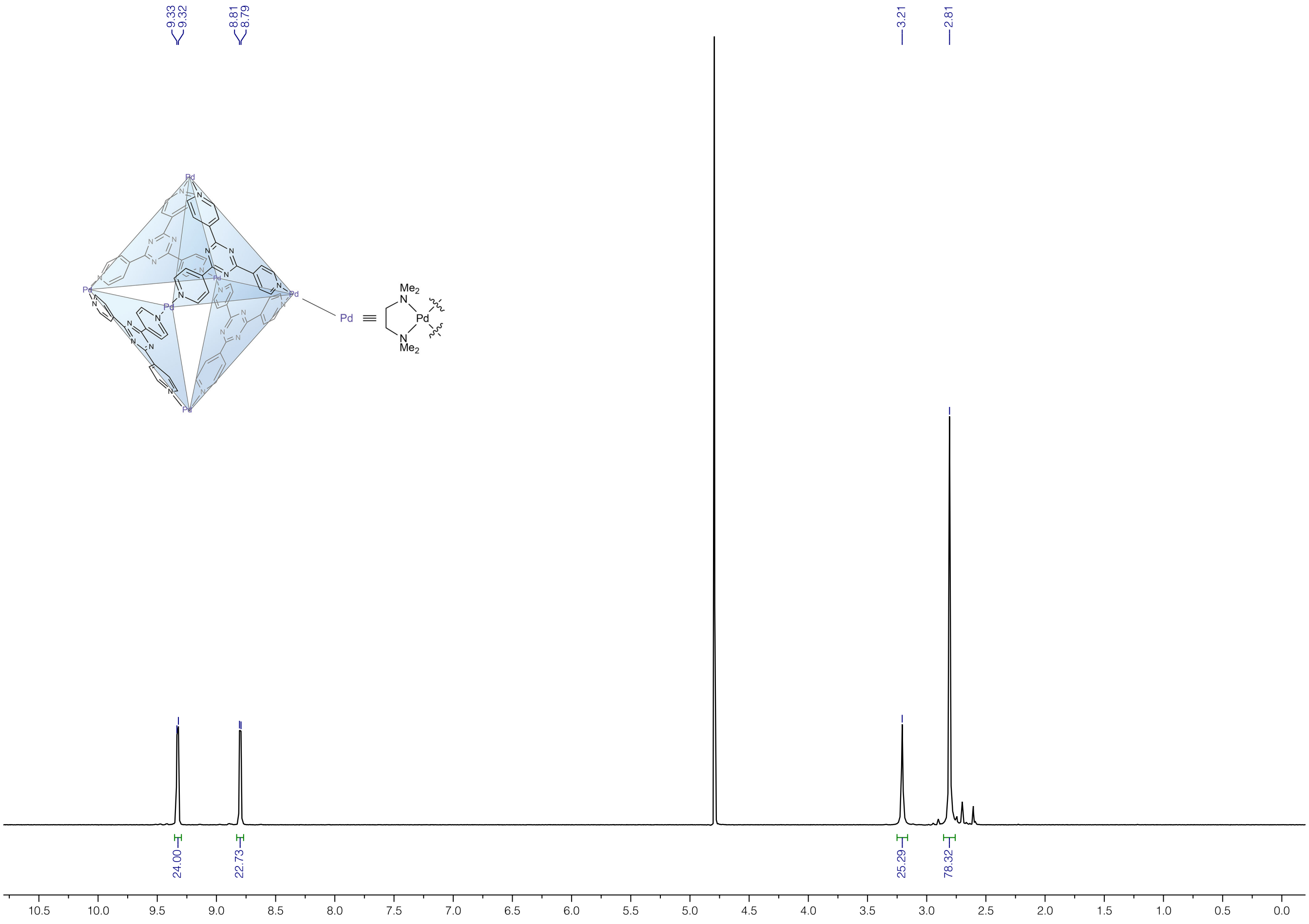

Supplementary Figure 1 | ^{1}H NMR spectrum of **2** (500 MHz, D_2O).

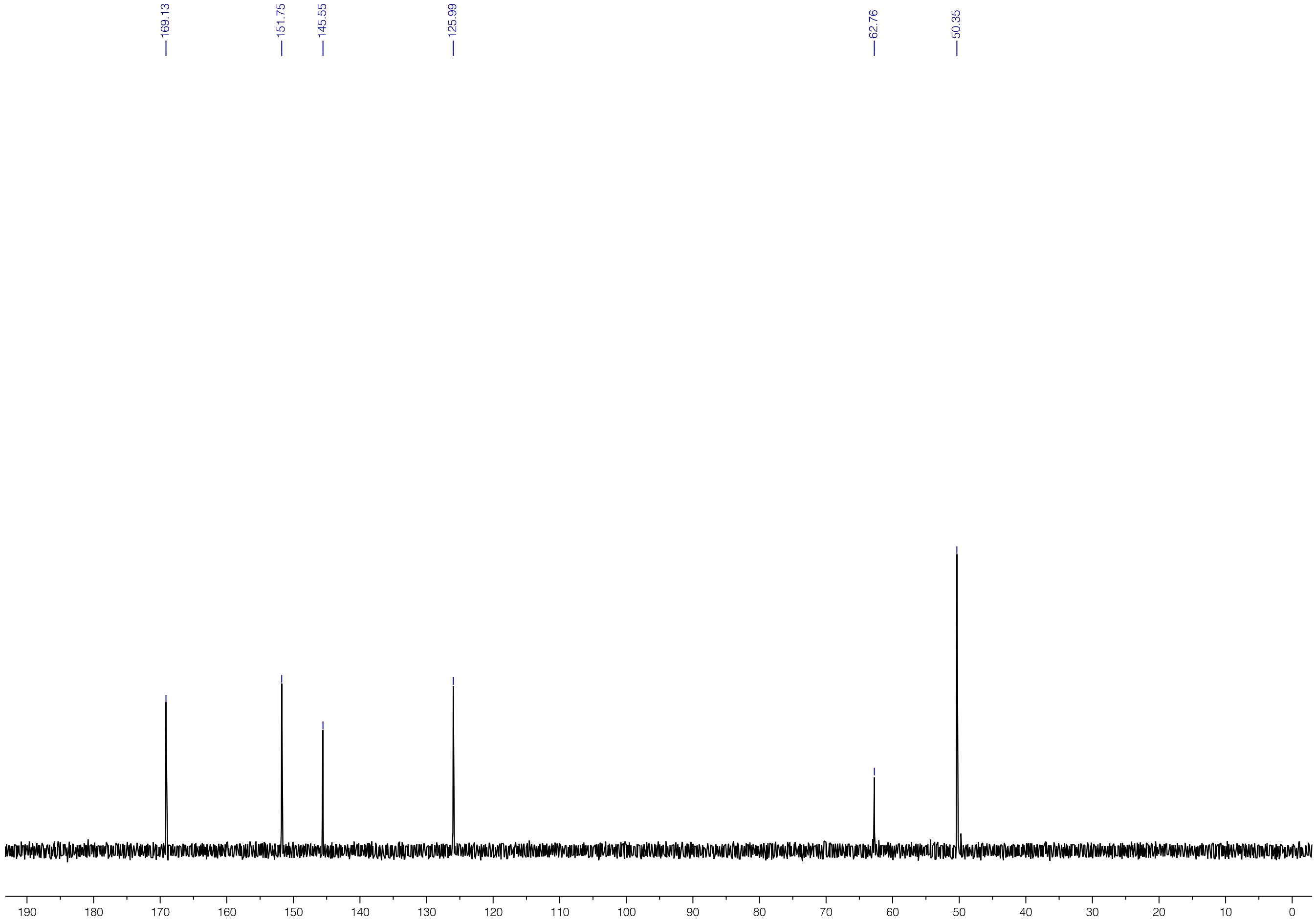

Supplementary Figure 2 | ^{13}C NMR spectrum of **2** (125 MHz, D_2O).

Supplementary Note 3 | Characterization of cage **4**.

^{1}H NMR (500 MHz, D_2O): δ = 9.10 (s, 8H, $\mathbf{4_4}$), 8.82 (s, 4H, $\mathbf{4_1}$), 7.74 (s, 4H, $\mathbf{4_2}$), 7.71 (s, 4H, $\mathbf{4_3}$), 7.69 (s, 8H, $\mathbf{4_5}$), 7.64 (s, 8H, $\mathbf{4_6}$), 7.54 (s, 12H, $\mathbf{4_{7+8}}$), 3.11 (s, 24H, $\mathbf{4}_{CH2}$), 2.77–2.70 (m, 72H, $\mathbf{4}_{CH3}$). **^{13}C NMR** (100 MHz, D_2O): δ = 138.04 ($\mathbf{4_9}$), 137.75 ($\mathbf{4_{10}}$), 137.49 ($\mathbf{4_4}$), 137.25 ($\mathbf{4_1}$), 128.73 ($\mathbf{4_{7+8}}$), 120.74 ($\mathbf{4_6}$), 120.52 ($\mathbf{4_3}$), 114.79 ($\mathbf{4_2}$), 113.09 ($\mathbf{4_5}$), 62.52 ($\mathbf{4}_{CH2}$), 50.29 ($\mathbf{4}_{CH3}$), 50.24 ($\mathbf{4}_{CH3}$), 50.02 ($\mathbf{4}_{CH3}$).

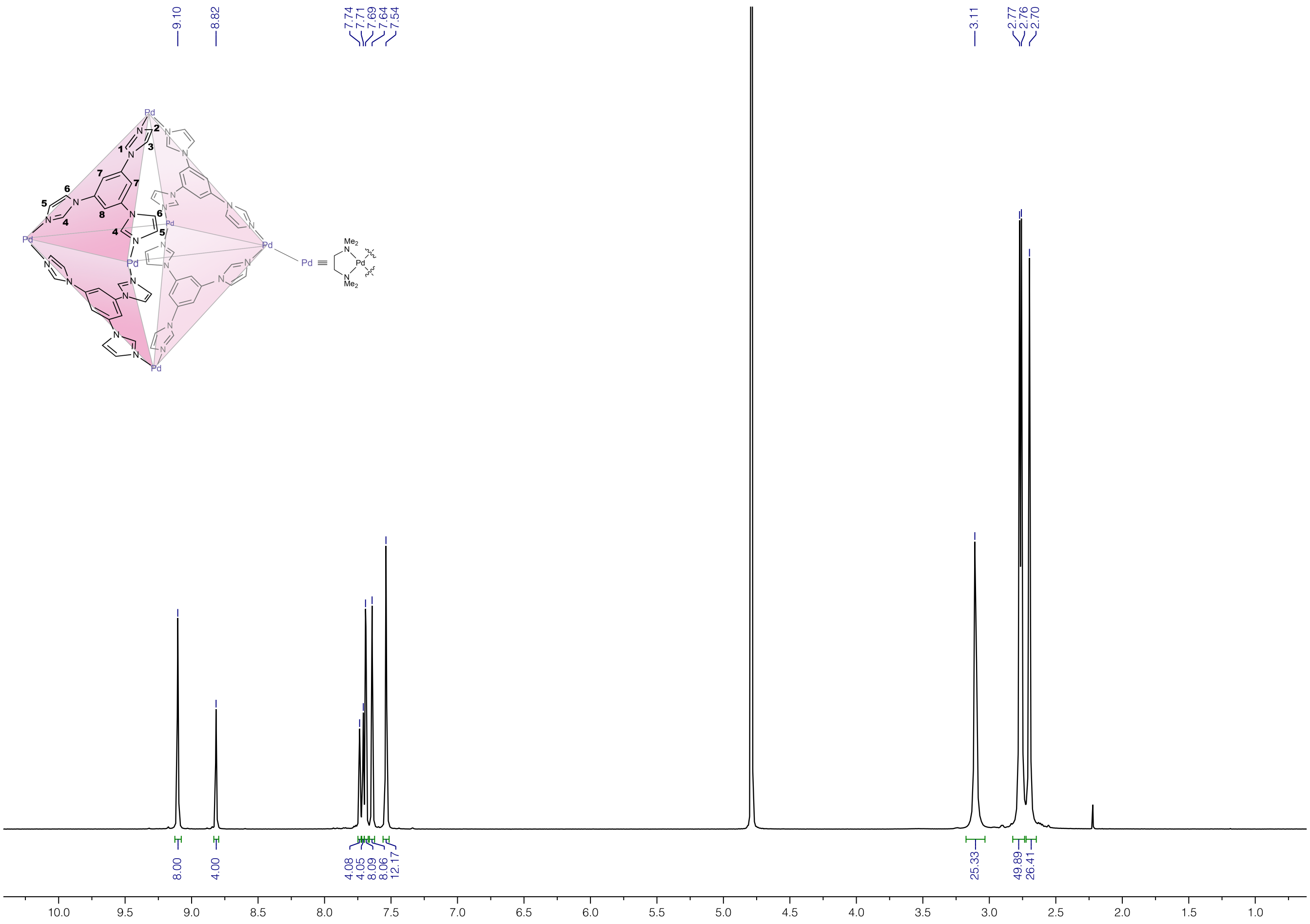

Supplementary Figure 3 | ^{1}H NMR spectrum of **4** (500 MHz, D_2O). For signal assignment, see Supplementary Note 3.

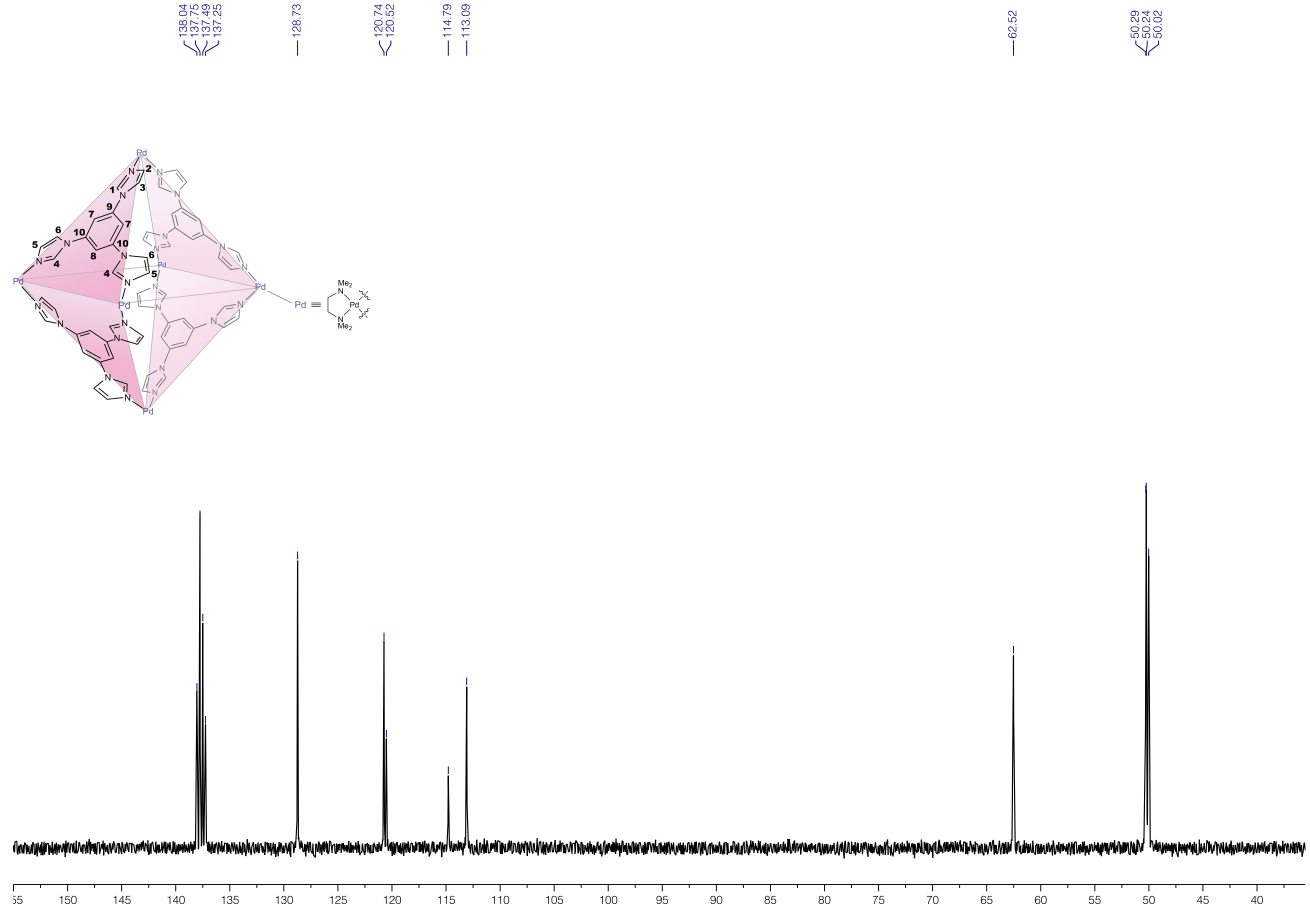

Supplementary Figure 4 | ^{13}C NMR spectrum of **4** (125 MHz, D_2O). For signal assignment, see Supplementary Note 3.

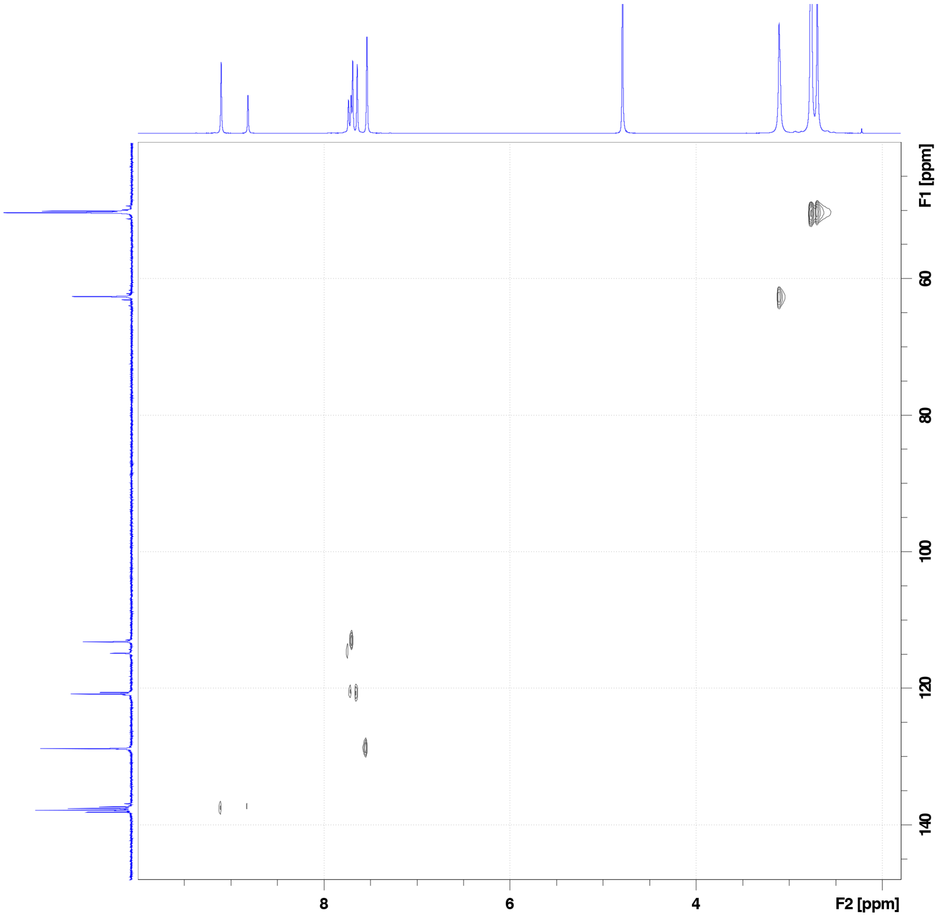

Supplementary Figure 5 | ^{1}H-^{13}C HSQC NMR spectrum of **4** (500 MHz, D_2O).

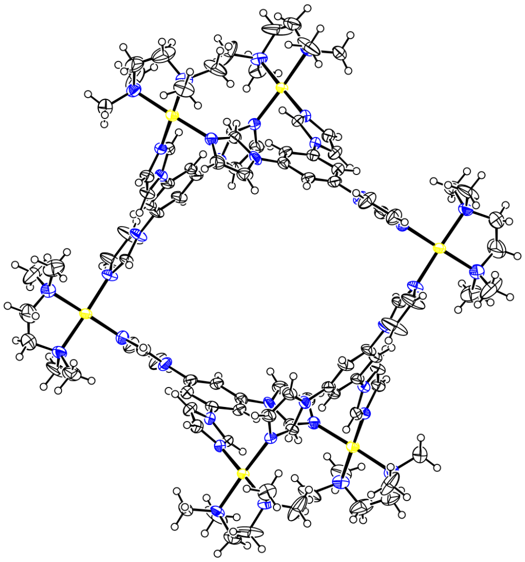

Supplementary Figure 6 | ORTEP representation of the X-ray structure of cage **4** (thermal ellipsoids at a 50% probability level). Anions and solvent molecules were eliminated for clarity. Pd, yellow; C white; N, blue; H, small white balls.

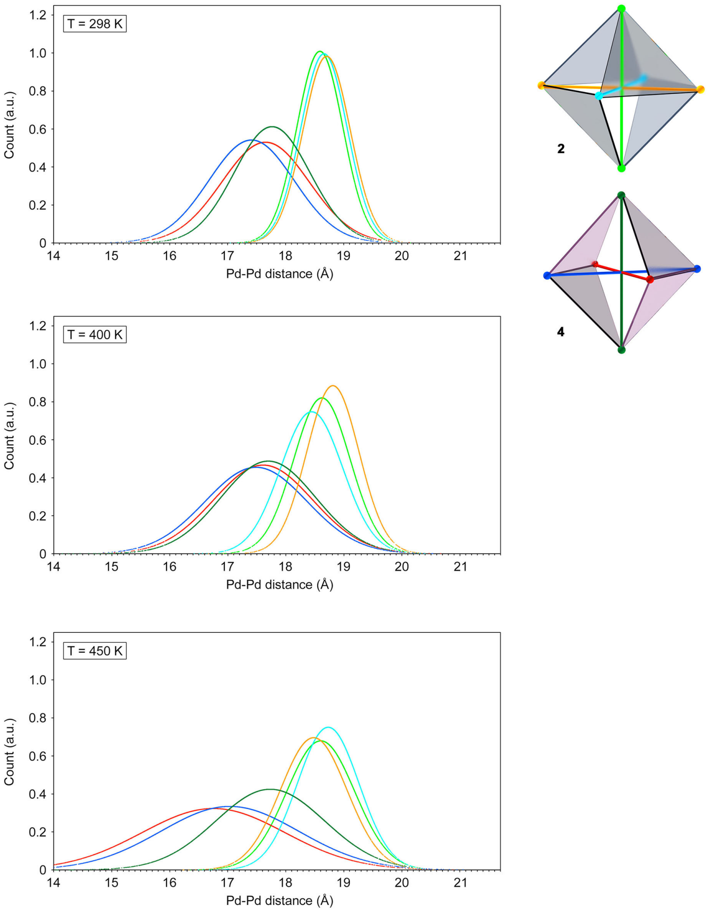

Supplementary Figure 7 | Calculated distributions of Pd–Pd distances in cages **2** and **4** at T = 298 K, 400 K, and 450 K. Movies prepared based on atomistic molecular dynamics simulations of cage **2** and **4** can be downloaded from https://www.dropbox.com/s/ogmnrp27y71buh3/2.mov and https://www.dropbox.com/s/sa41typlu0j6all/4.mov, respectively.

Supplementary Note 4 | Synthesis and characterization of guest **5**.

Guest **5** (3-(2-(2-hydroxystyryl)-3,3-dimethyl-3*H*-indol-1-ium-1-yl)propane-1-sulfonate) was synthesized according to a modified literature procedure[2]. 2,3,3-trimethylindolenine (6.36 g, 0.04 mol) was mixed with 1,3-propanesultone (4.88 g, 0.04 mol) and the mixture was stirred at 90 °C for 6 h under a nitrogen atmosphere. The resulting purple solid was filtered and washed several times with diethyl ether to afford 2,3,3-trimethyl-1-(3-sulfonatepropyl)-3*H*-indolium (isolated yield: 90%). Next, 2,3,3-trimethyl-1-(3-sulfonatepropyl)-3*H*-indolium (600 mg, 2.14 mmol) and 2-hydroxybenzaldehyde (292 mg, 2.40 mmol) were added to anhydrous ethanol (12 mL) and refluxed overnight. The resulting orange solid was isolated by filtration and washed with ethanol. Isolated yield: 82%.

^{1}H NMR (400 MHz, $(CD_3)_2SO$): δ = 11.04 (s, 1H), 8.61 (d, ^3J = 16.4 Hz, 1H), 8.29 (d, ^3J = 7.8 Hz, 1H), 8.03 (d, ^3J = 7.4 Hz, 1H), 7.87 (m, 2H), 7.63 (m, 2H), 7.48 (t, ^3J = 7.7 Hz, 1H), 7.05 (d, ^3J = 8.3 Hz, 1H), 7.00 (t, ^3J = 7.6 Hz, 1H), 4.82 (t, ^3J = 7.6 Hz, 2H), 2.66 (t, ^3J = 6.2 Hz, 2H), 2.19 (m, 2H), 1.78 (s, 6H). **^{13}C NMR** (100 MHz, $(CD_3)_2SO$): δ = 182.31, 159.54, 149.21, 144.00, 141.45, 136.26, 130.30, 129.66 (2C), 123.50, 121.86, 120.58, 117.14, 115.61, 111.98, 52.38, 47.85, 46.06, 26.96 (2C), 25.11. **HRMS** calcd for $C_{21}H_{22}NO_4S$ $[M - H]^-$, m/z = 384.1270, found 384.1267.

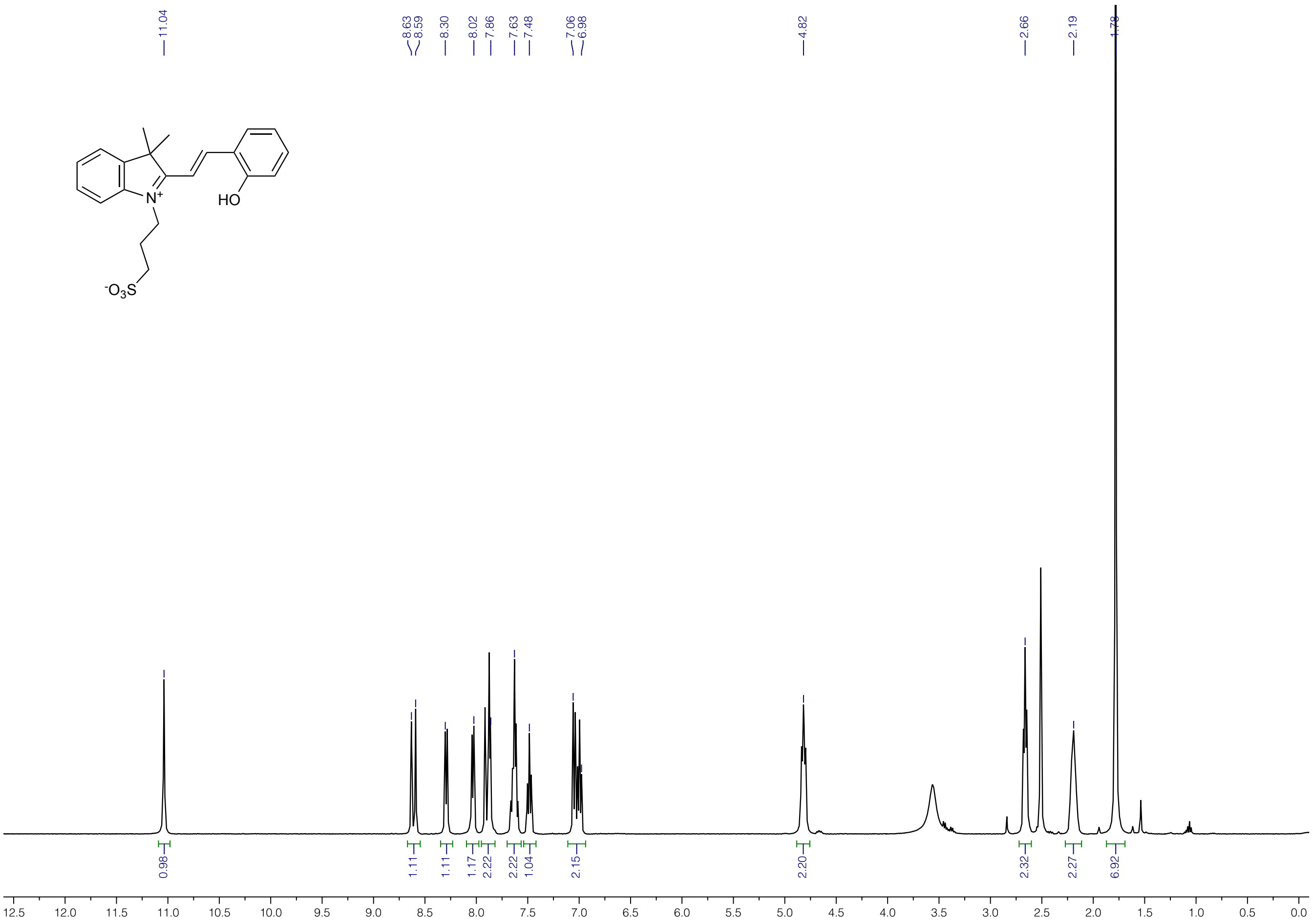

Supplementary Figure 8 | ^{1}H NMR spectrum of **5** (400 MHz, $(CD_3)_2SO$).

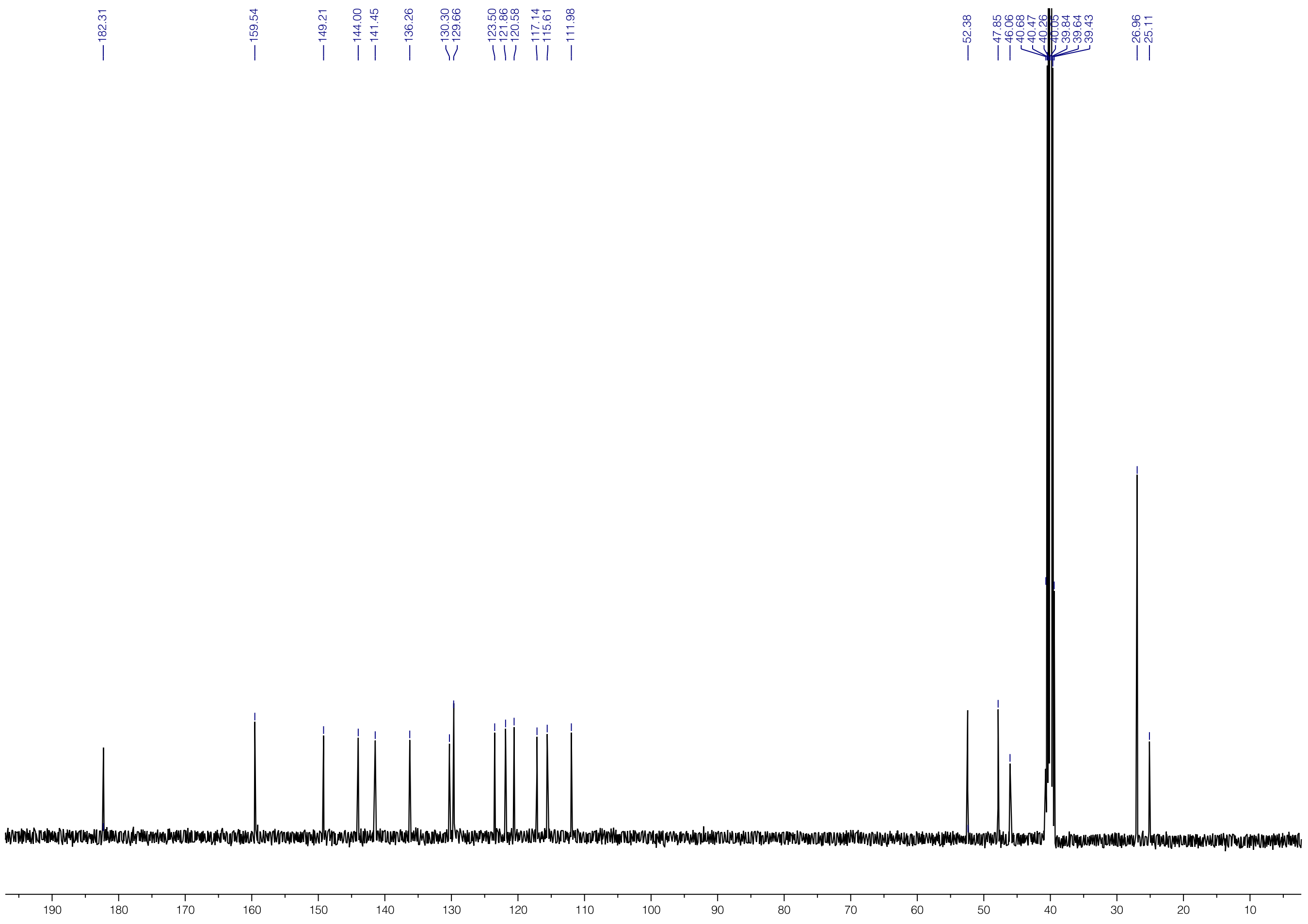

Supplementary Figure 9 | ^{13}C NMR spectrum of **5** (100 MHz, $(CD_3)_2SO$).

Supplementary Note 5 | Synthesis and characterization of guest **6**.

Guest **6** (1′,3′,3′-trimethylspiro[chromene-2,2′-indoline]) was synthesized according to a modified literature procedure[3]. An acetonitrile solution (45 mL) of 2,3,3-trimethylindolenine (3.85 g, 24.18 mmol) and methyl iodide (8.6 g, 60.58 mmol) was refluxed for 24 h under an argon atmosphere. Then, the reaction mixture was concentrated under reduced pressure and triturated with ether (60 mL) to yield a solid powder. The powder was washed several times with ether (3 × 60 mL), and dried under high vacuum. An aqueous solution of KOH was added to the resulting solid, and stirred for 30 min. It was then extracted with diethyl ether to afford 1,3,3-trimethyl-2-methyleneindoline (isolated yield: 87%). Next, 1,3,3-trimethyl-2-methyleneindoline (2.0 g, 11.5 mmol) and salicylaldehyde (1.80 g, 14.73 mmol) were dissolved in ethanol (130 mL) and refluxed under a nitrogen atmosphere for 15 h. The solvent was removed under reduced pressure and the residue was dissolved in CH_2Cl_2. It was then washed with aqueous NaOH and the resulting crude product was purified by column chromatography to afford pure **6** as a crystalline solid. Isolated yield: 87%.

^{1}H NMR (400 MHz, CD_2Cl_2): δ = 7.15 (t, ^3J = 7.6 Hz, 1H), 7.08 (m, 3H), 6.89 (d, ^3J = 10.2 Hz, 1H), 6.83 (t, ^3J = 7.3 Hz, 2H), 6.69 (d, ^3J = 8.1 Hz, 1H), 6.52 (d, ^3J = 7.7 Hz, 1H), 5.71 (d, ^3J = 10.2 Hz, 1H), 2.73 (s, 3H), 1.29 (s, 3H), 1.16 (s, 3H). **^{13}C NMR** (100 MHz, (CD_2Cl_2): δ = 154.48, 148.36, 136.89, 129.71, 129.49, 127.57, 126.77, 121.53, 120.11, 119.31, 119.07, 118.97, 114.74, 106.75, 104.50, 51.74, 28.70, 25.64, 19.80.

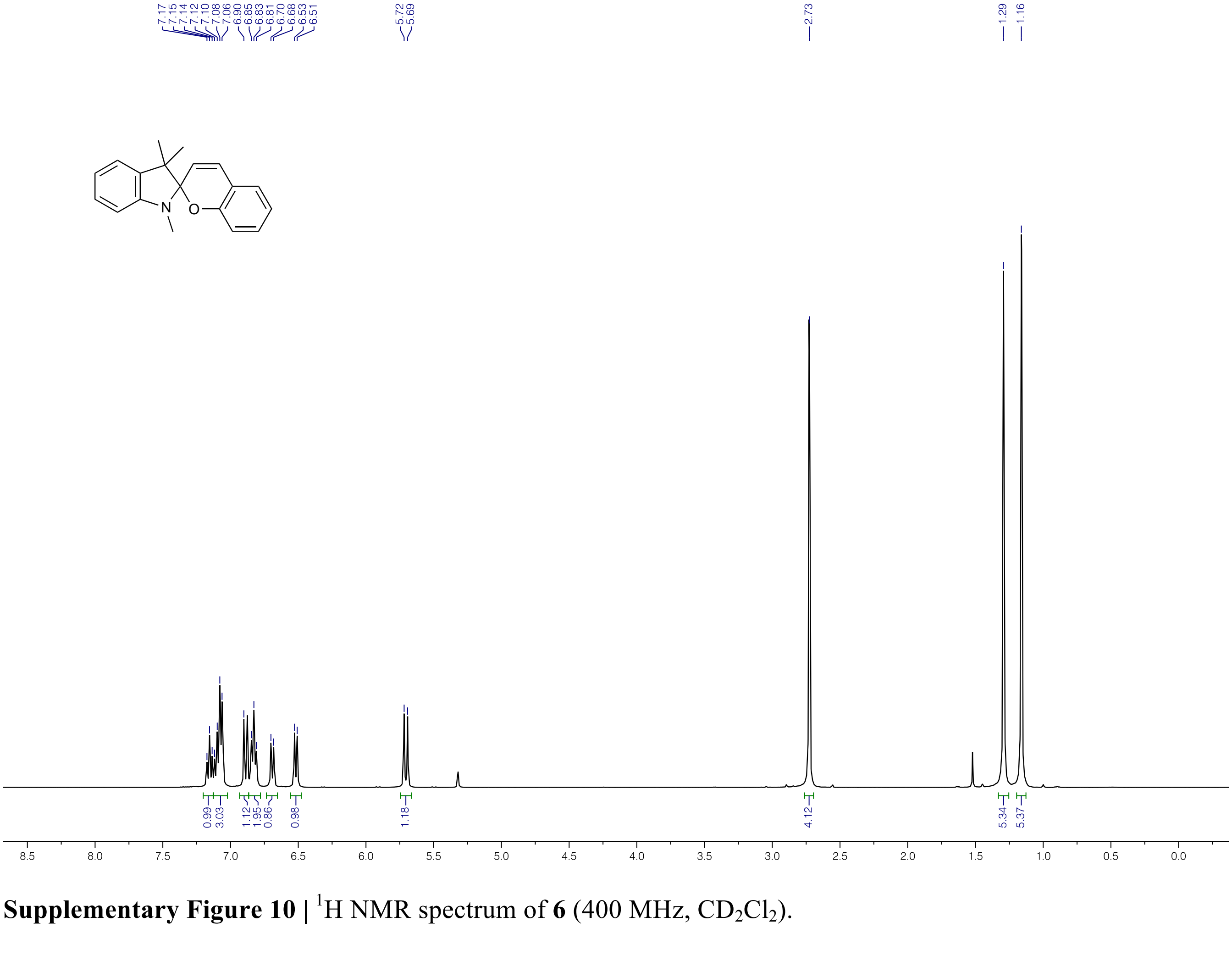

Supplementary Figure 10 | ^{1}H NMR spectrum of **6** (400 MHz, CD_2Cl_2).

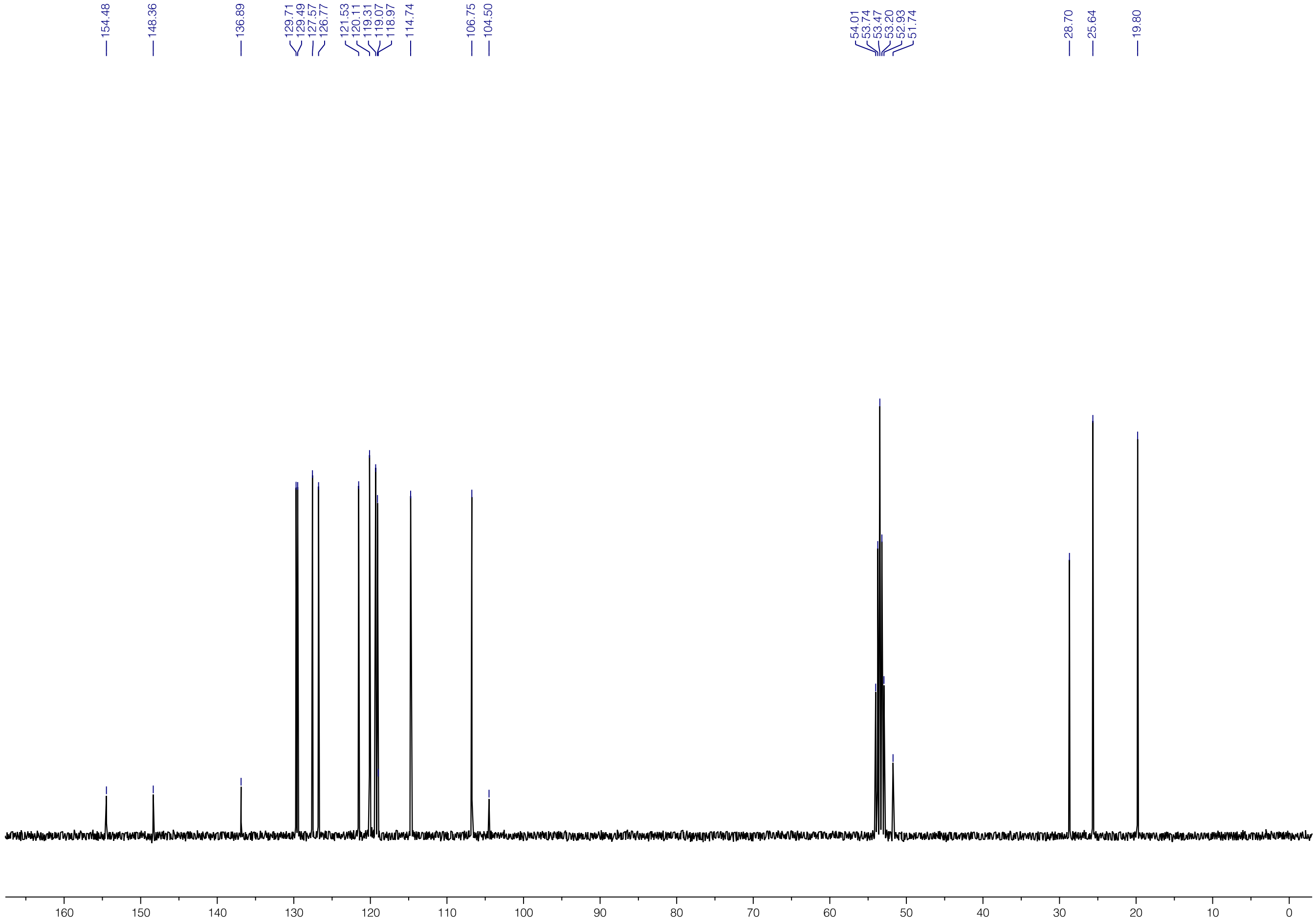

Supplementary Figure 11 | ^{13}C NMR spectrum of **6** (100 MHz, CD_2Cl_2).

Supplementary Note 6 | Synthesis of guest **7**.

Guest **7** (1′,3′,3′-trimethyl-6-nitrospiro[chromene-2,2′-indoline]) was synthesized based on a previously reported literature procedure[4].

Supplementary Note 7 | Synthesis and characterization of guest **8**.

Guest **8** (1′,3′,3′,8-tetramethylspiro[chromene-2,2′-indoline]) was synthesized by modifying a previously reported literature procedure[3]. An acetonitrile solution (45 mL) of 2,3,3-trimethylindolenine (3.85 g, 24.18 mmol) and methyl iodide (8.6 g, 60.58 mmol) was refluxed for 24 h under an argon atmosphere. Then, the reaction mixture was concentrated under reduced pressure and triturated with ether (60 mL) to yield a powder. The solid powder was washed several times with ether (3 × 60 mL) and dried under high vacuum. An aqueous solution of KOH was added to the resulting solid, and stirred for 30 min. It was then extracted with diethyl ether to afford 1,3,3-trimethyl-2-methyleneindoline (isolated yield: 87%). Next, 1,3,3-trimethyl-2-methyleneindoline (2.0 g, 11.5 mmol) and 2-hydroxy-3-methylbenzaldehyde (2.0 g, 14.7 mmol) were dissolved in ethanol (100 mL) and refluxed under a nitrogen atmosphere for 20 h. The solvent was removed under reduced pressure and the residue was dissolved in CH_2Cl_2. It was then washed twice with aqueous NaOH and the resulting crude product was purified by column chromatography to afford pure **8**. Isolated yield: 81%.

^{1}H NMR (500 MHz, CD_2Cl_2): δ = 7.13 (t, ^3J = 7.6 Hz, 1H), 7.06 (d, ^3J = 7.2 Hz, 1H), 6.96 (d, ^3J = 7.5 Hz, 1H), 6.91 (d, ^3J = 7.5 Hz, 1H), 6.86 (d, ^3J = 10.2 Hz, 1H), 6.80 (t, ^3J = 7.3 Hz, 1H), 6.73 (t, ^3J = 7.5 Hz, 1H), 6.50 (d, ^3J = 7.7 Hz, 1H), 5.68 (d, ^3J = 10.2 Hz, 1H), 2.68 (s, 3H), 1.95 (s, 3H), 1.27 (s, 3H), 1.16 (s, 3H). **^{13}C NMR** (125 MHz, (CD_2Cl_2): δ = 151.90, 148.31, 136.98, 130.81, 129.48, 127.34, 124.37, 123.96, 121.35, 119.46, 118.91, 118.84, 118.29, 106.58, 104.16, 51.36, 28.58, 25.57, 19.91, 14.82. **HRMS** calcd for $C_{20}H_{22}NO$ $[M + H]^+$, m/z = 292.1701, found 292.1700.

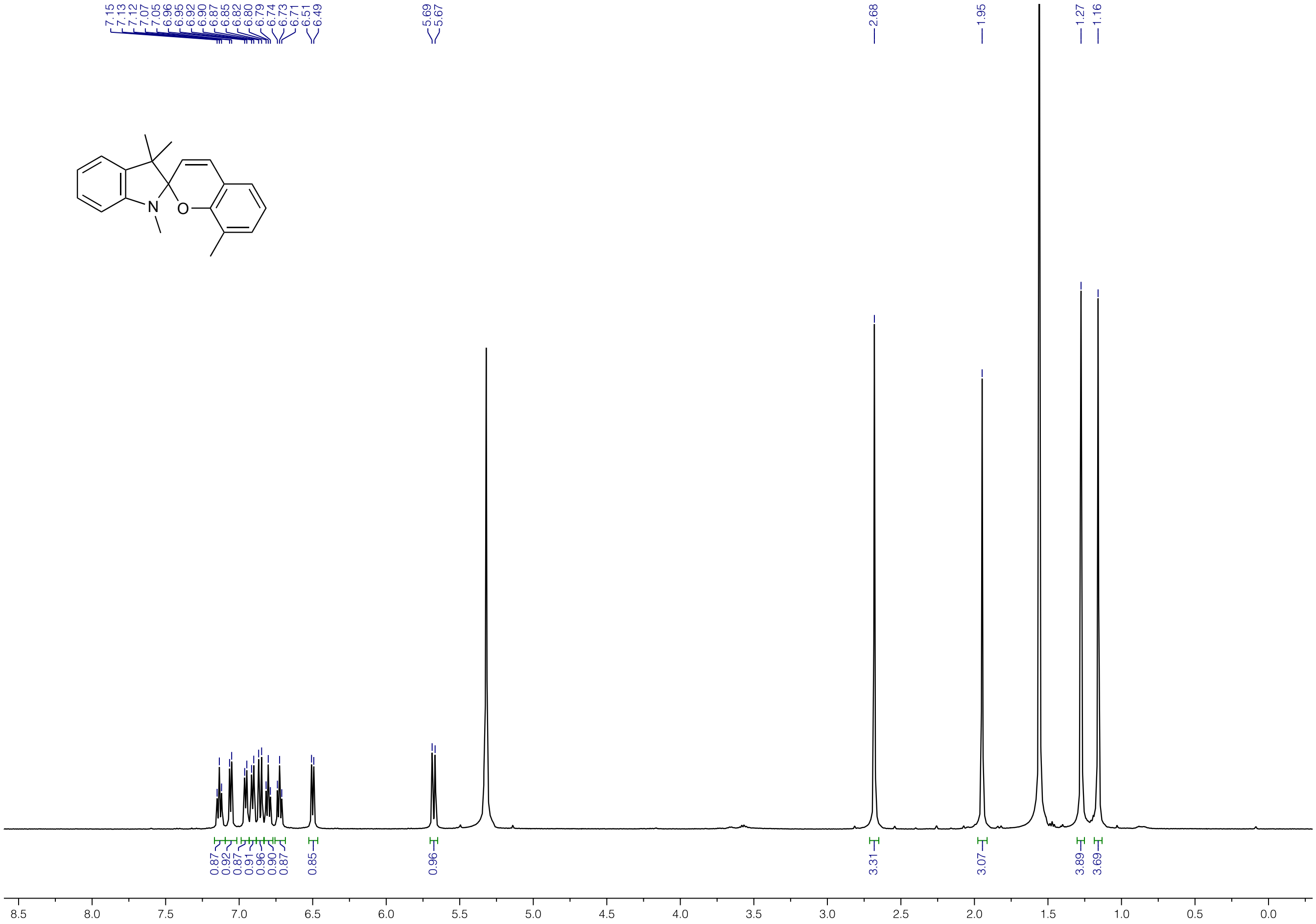

Supplementary Figure 12 | ^{1}H NMR spectrum of **8** (500 MHz, CD_2Cl_2).

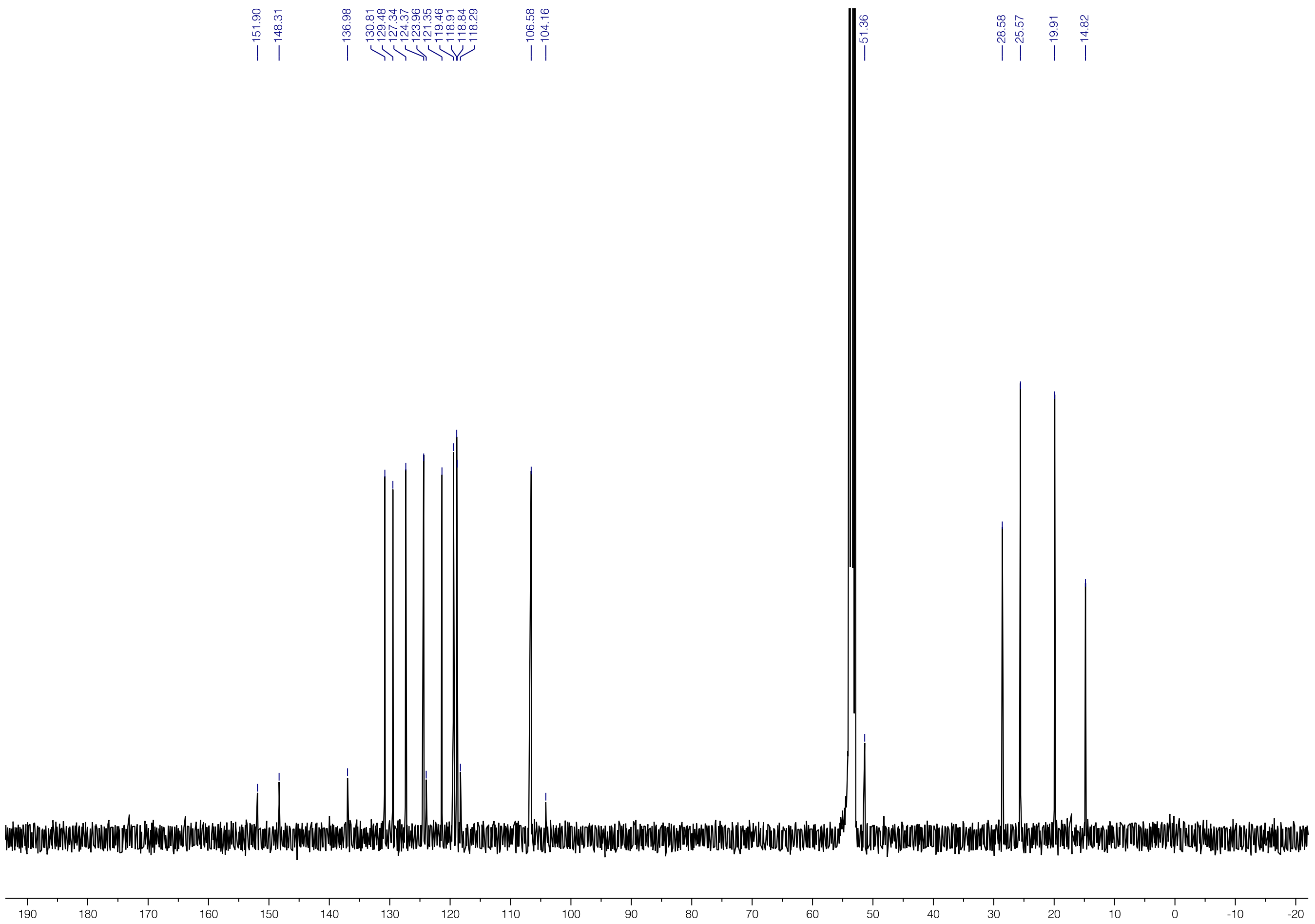

Supplementary Figure 13 | ^{13}C NMR spectrum of **8** (125 MHz, CD_2Cl_2).

Supplementary Note 8 | Characterization of inclusion complex **5**⊂**4**.

Inclusion complex **5**⊂**4** was obtained in ~100% yield.

^{1}H NMR (500 MHz, D_2O): δ = 9.33 (br, 4H, $\mathbf{4}_{\mathbf{1}}$), 9.13 (s, 8H, $\mathbf{4}_{\mathbf{4}}$), 7.97 (br, 4H, $\mathbf{4}_{\mathbf{2}}$), 7.77 (s, 4H, $\mathbf{4}_{\mathbf{8}}$), 7.74 (br, 12H, $\mathbf{4}_{\mathbf{6+3}}$), 7.62 (br, 16H, $\mathbf{4}_{\mathbf{5+7}}$), 7.32–6.40 (br, 10H, **5**), 3.92–3.50 (br, 2H, $\mathbf{5}_{\mathbf{m}}$), 3.17 (s, 8H, $\mathbf{4}_{\mathrm{CH2}}$), 3.13 (br, 2H, $\mathbf{5}_{\mathbf{n}}$), 3.07 (s, 16H, $\mathbf{4}_{\mathrm{CH2}}$), 2.82 (s, 24H, $\mathbf{4}_{\mathrm{CH3}}$), 2.72 (s, 24H, $\mathbf{4}_{\mathrm{CH3}}$), 2.61 (s, 24H, $\mathbf{4}_{\mathrm{CH3}}$), 1.51–1.22 (br, 2H, $\mathbf{5}_{\mathbf{o}}$), 0.13 (s, 6H, $\mathbf{5}_{\mathbf{k+l}}$). **^{13}C NMR** (200 MHz, D_2O): δ = 181.45 (**5**), 160.19 (**5**), 153.87 (**5**), 141.79 (**5**), 141.73 (**5**), 140.77 (**5**), 138.54 (**5**), 137.96 (**4**), 137.78 (**4**), 137.41 (**4**), 137.06 (**4**), 129.85 (**5**), 129.48 (**4**), 129.11 (**4**), 123.31 (**5**), 120.73 (**4**), 120.30 (**5**), 119.69 (**4**), 118.32 (**5**), 117.73 (**5**), 114.87 (**5**), 113.12 (**4**), 112.48 (**5**, 2C), 62.56 (**4**), 62.46 (**4**), 57.36 (**5**), 50.22 (**4**), 49.53 (**4**), 47.40 (**5**), 46.41 (**5**), 44.18 (**5**), 23.51(**5**). **^{1}H-DOSY NMR** (500 MHz, D_2O, 298 K): D = 0.19 (± 0.01) × 10^{-5} cm^2/s. **Elemental analysis**: calcd: C, 39.36; H, 4.71; N, 19.22; found: 39.40; H, 4.82; N, 19.26.

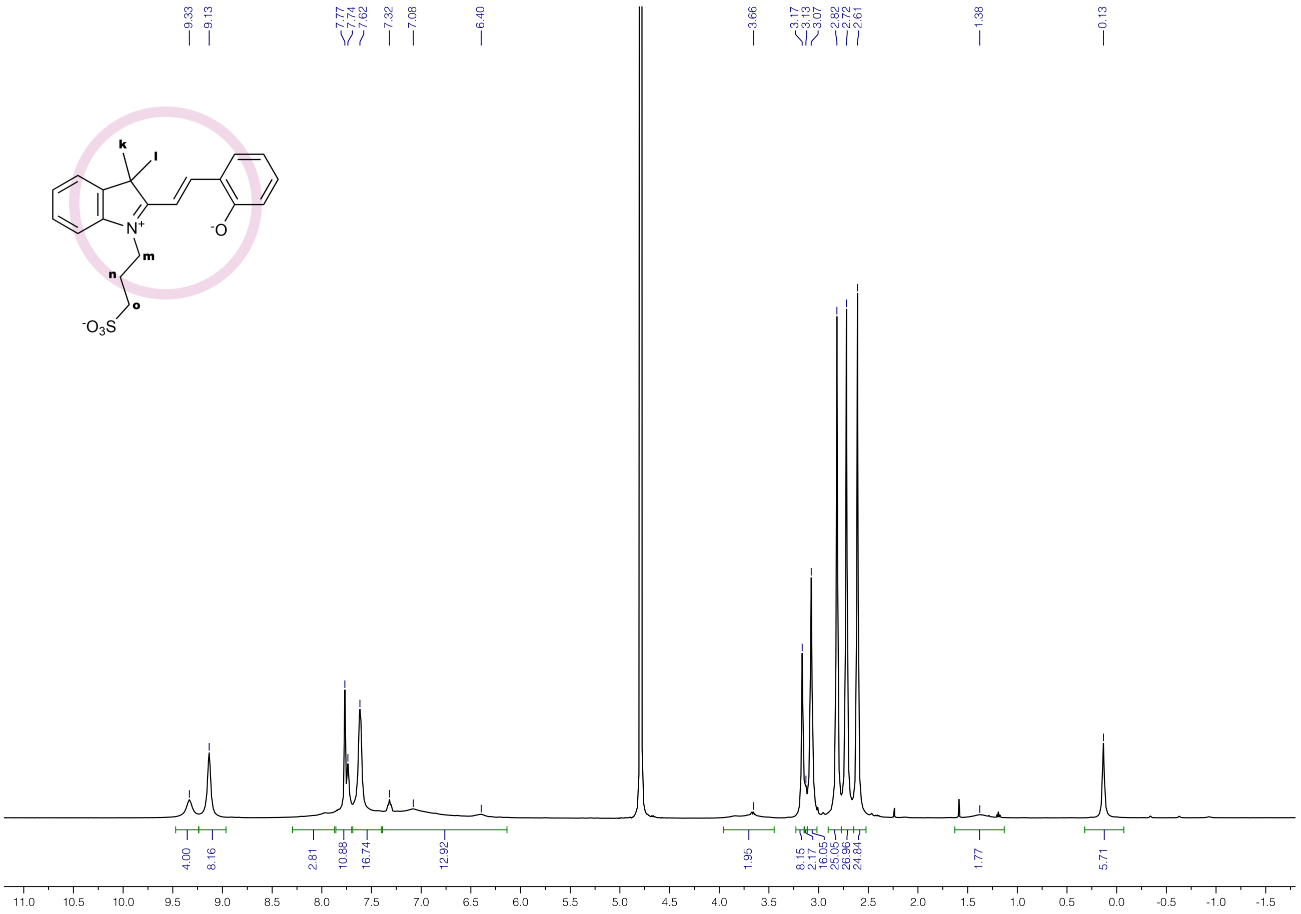

Supplementary Figure 14 | ^{1}H NMR spectrum of **5**⊂**4** (500 MHz, D_2O). For signal assignment, see Supplementary Note 8.

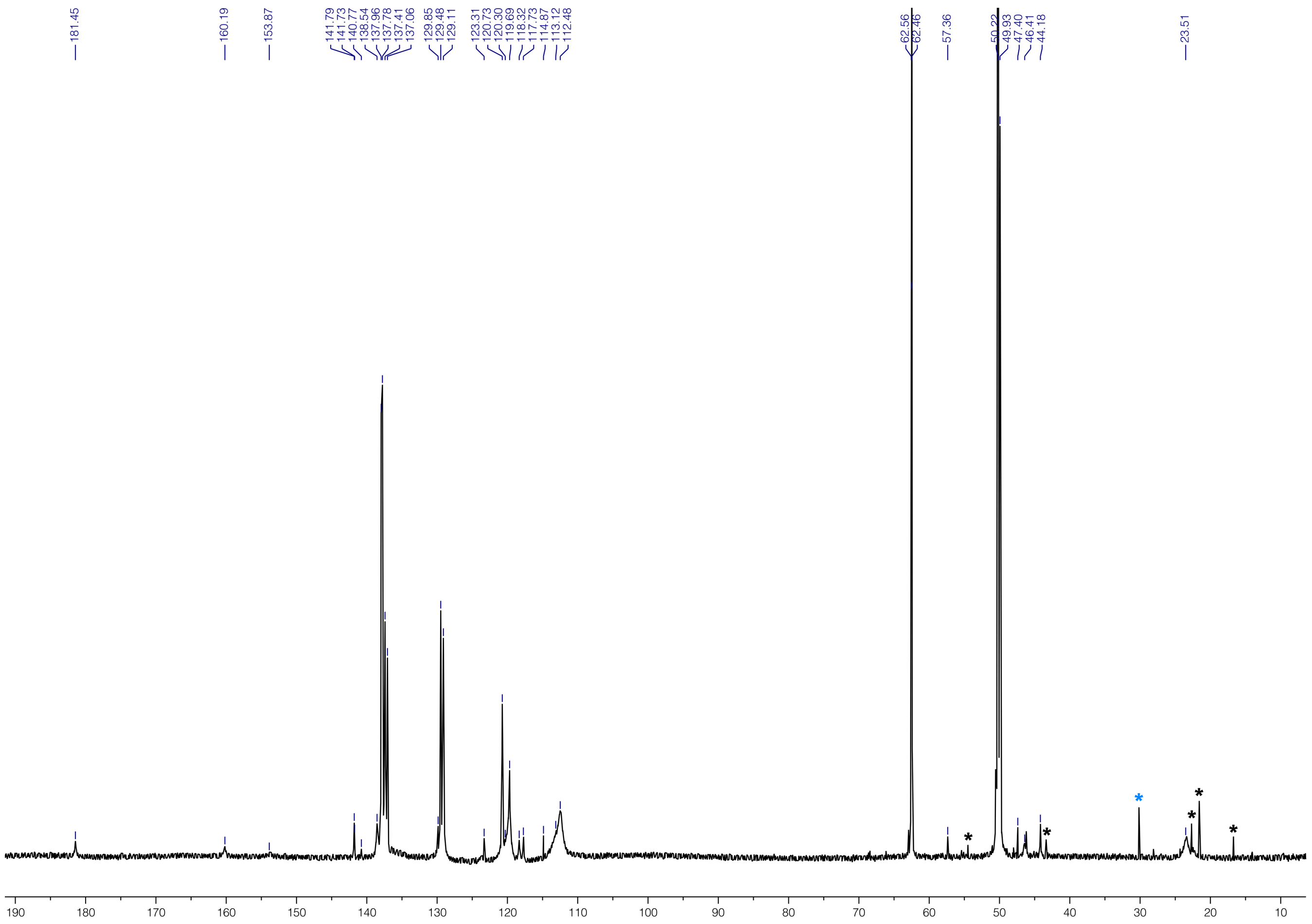

Supplementary Figure 15 | ^{13}C NMR spectrum of **5**$\subset$**4** (after baseline correction) (200 MHz, D_2O). The blue asterisk denotes acetone. The black asterisks denote contaminations.

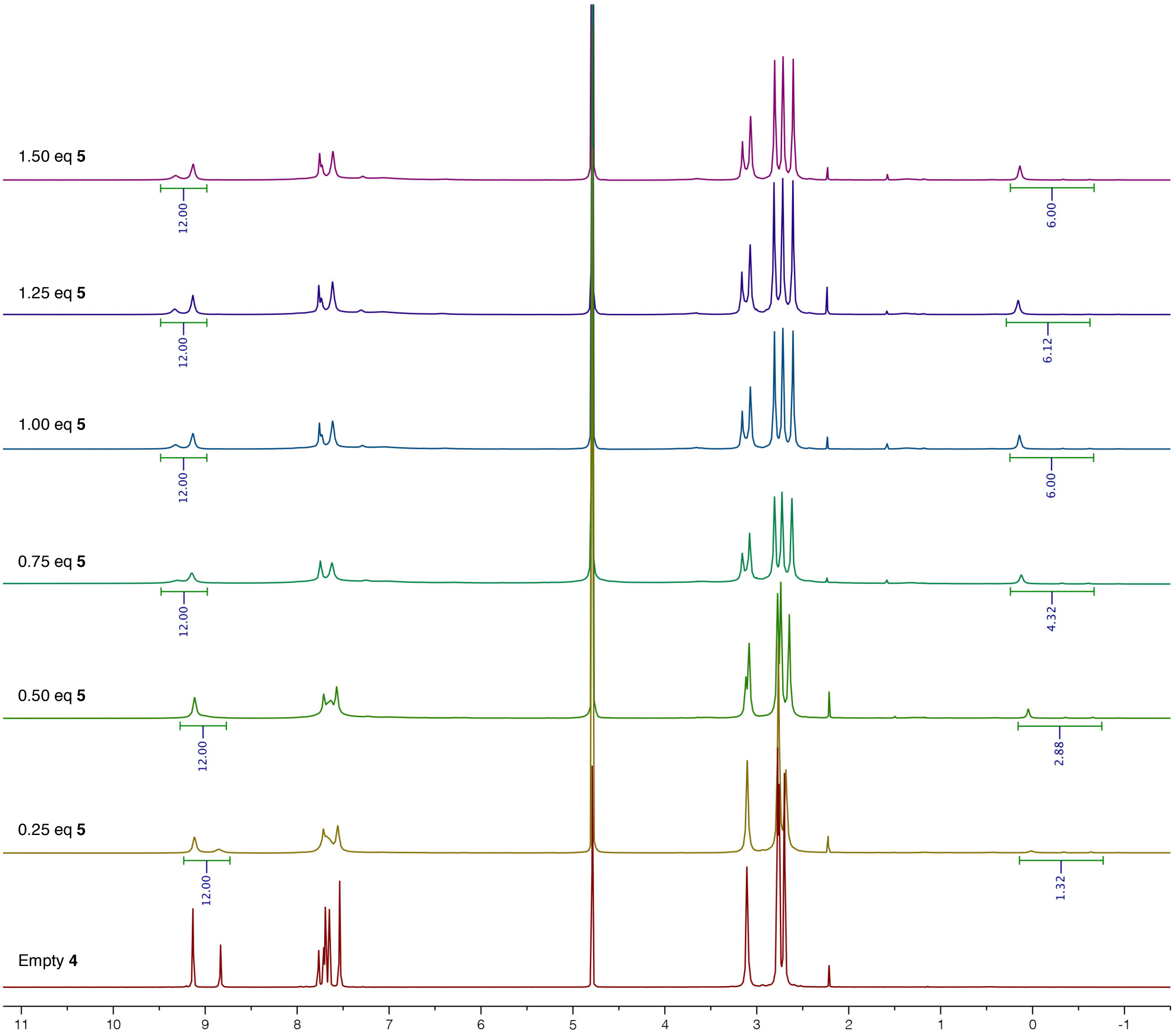

Supplementary Figure 16 | Changes in the ^{1}H NMR spectra of **4** (400 MHz, D_2O) in the presence of increasing amounts of **5**. The spectrum at the bottom corresponds to pure **4**. Subsequent spectra (from the bottom to the top) were recorded after addition of 0.25 eq, 0.50 eq, 0.75 eq, 1.00 eq, 1.25 eq, and 1.50 eq of **5**, followed by filtration to remove undissolved **5**.

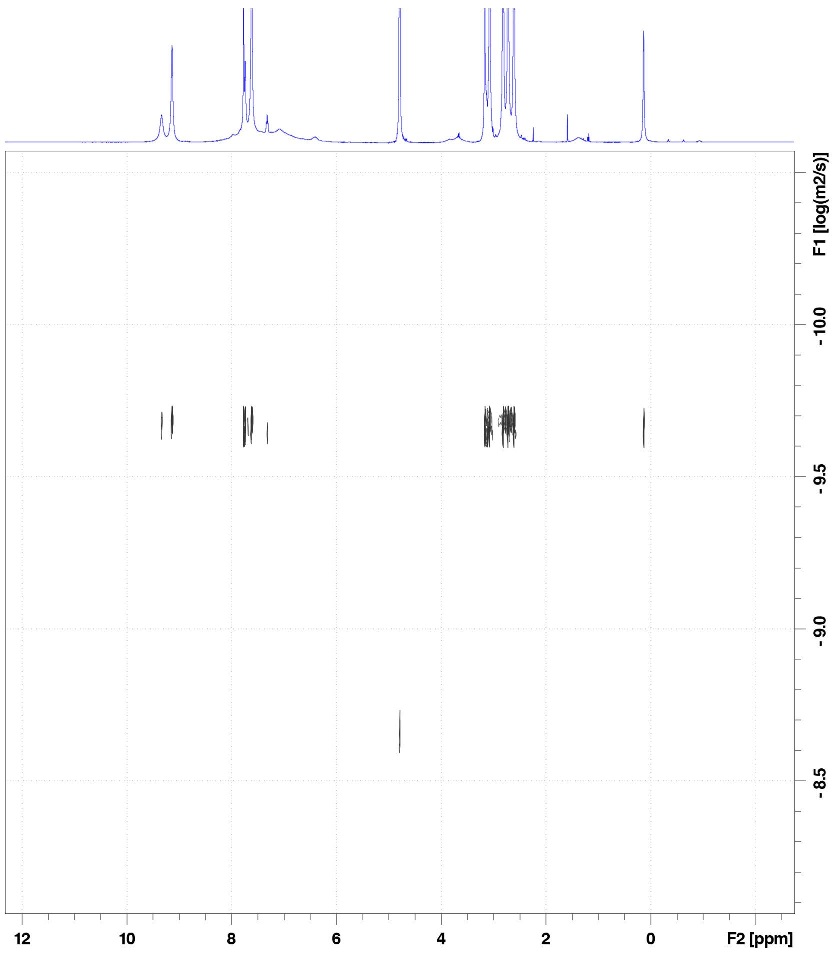

Supplementary Figure 17 | ^{1}H-DOSY NMR spectrum of **5**⊂**4** (500 MHz, D_2O).

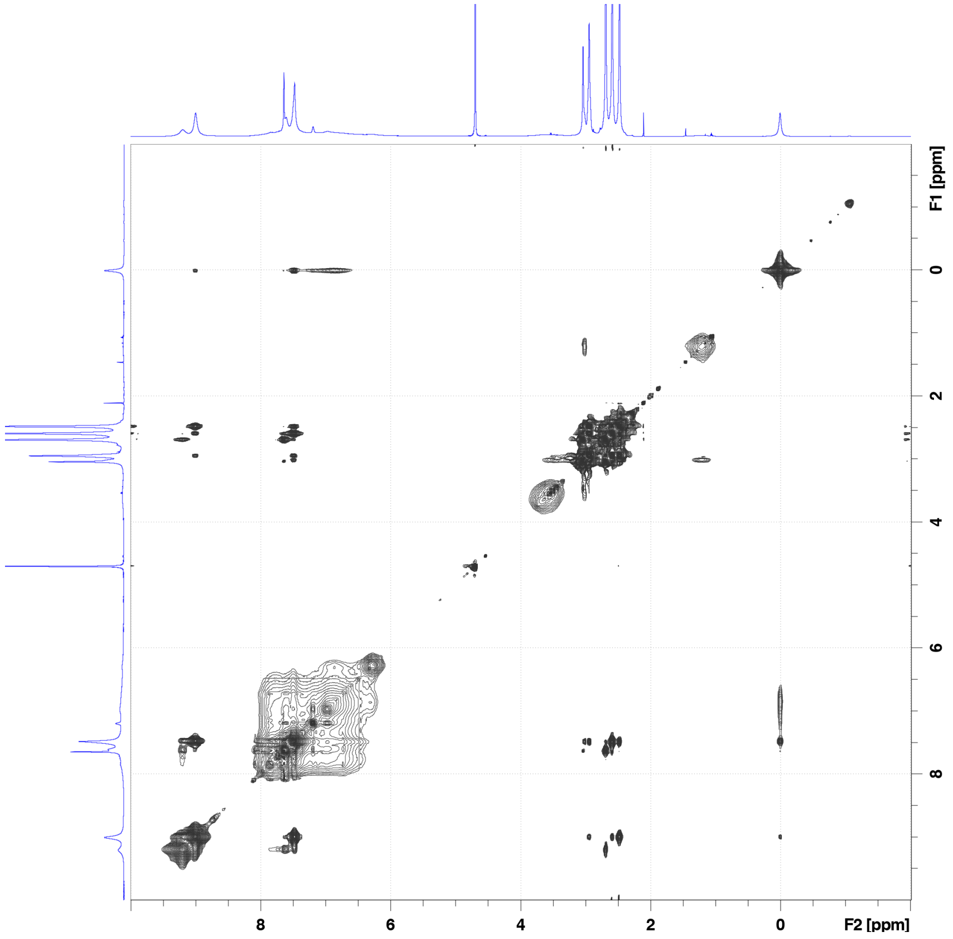

Supplementary Figure 18 | ^{1}H-^{1}H NOESY NMR spectrum of **5⊂4** (800 MHz, D_2O).

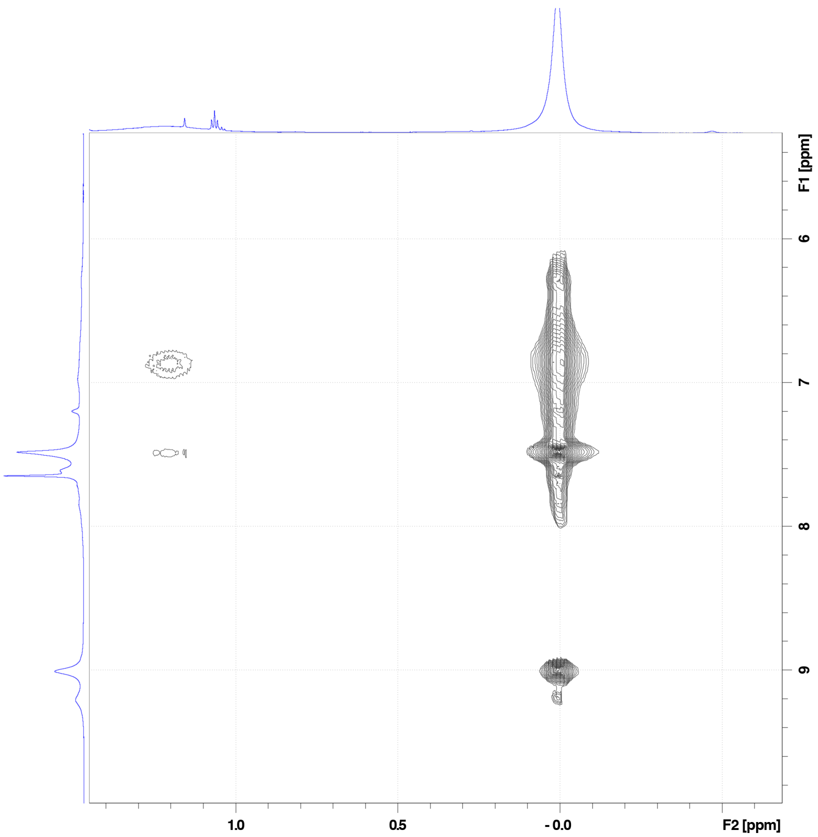

Supplementary Figure 19 | Partial ^{1}H-^{1}H NOESY NMR spectrum of **5**$\subset$**4** showing host-guest correlations (800 MHz, D_2O) (the corresponding full-range spectrum is shown in Supplementary Fig. 18). The signal at 0.0 ppm can be attributed to encapsulated **5**'s C**H**$_3$ protons and the broad signal centered at 1.2 ppm is most likely due to C**H**$_2$ protons adjacent to **5**'s sulfonate group. The signals at ~9.0 ppm and ~9.2 ppm are due to **4**'s acidic imidazole protons in the equatorial and apical positions, respectively, and the signal at ~7.5 ppm – due to other aromatic protons of **4**.

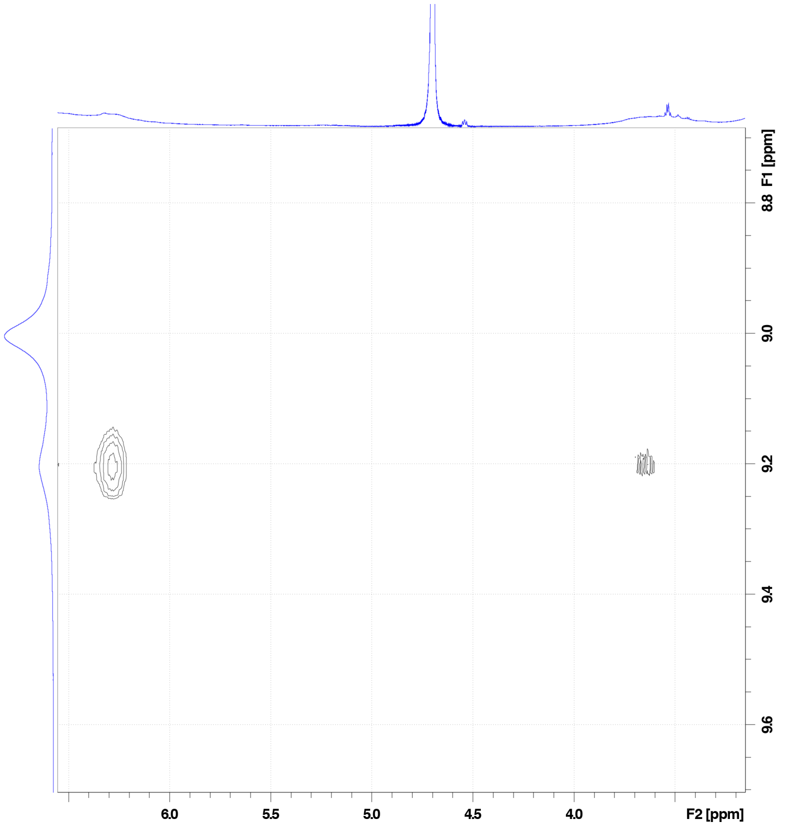

Supplementary Figure 20 | Partial ^{1}H-^{1}H NOESY NMR spectrum of **5**⊂**4** showing host-guest correlations (800 MHz, D_2O) (the corresponding full-range spectrum is shown in Supplementary Fig. 18). The signal centered at 9.2 ppm corresponds to **4**'s apical acidic imidazole protons. The broad signals centered at 3.6 ppm and 6.3 ppm are most likely due to encapsulated **5**'s =C**H**– protons and C**H**$_2$ protons adjacent to the N atom, respectively.

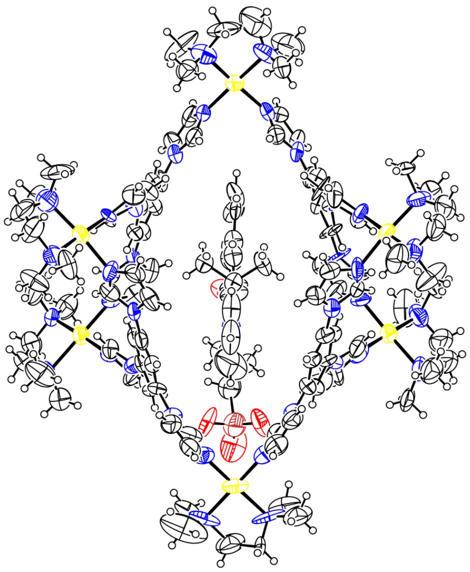

Supplementary Figure 21 | ORTEP representation of the X-ray structure of inclusion complex **5⊂4** (thermal ellipsoids at a 50% probability level). Anions and solvent molecules were eliminated for clarity**.** Pd, yellow; C white; N, blue; O, red; S, dark red; H, small white balls.

Supplementary Note 9 | Determination of the association constant of **5**⊂**4** by titrating guest **5** with cage **4**.

An aqueous solution of **5** (V = 1.0 mL; c = 5.3·10^{-6} M) was titrated with a 2.4·10^{-4} M aqueous solution of **4**. UV/Vis spectra were recorded after the addition of each 1.0 μL aliquot of **4** (Supplementary Fig. 22). Absorbance at 592 nm was plotted as a function of the amount of added **4**, and the association constant, K_{assoc}, was determined by applying a nonlinear method curve-fitting to absorption changes (ΔA) using[5,6] Supplementary Equation 1:

$$\Delta A = \frac{L(1+K_{\mathrm{assoc}}c_4+K_{\mathrm{assoc}}c_5)-\sqrt{\mathrm{L}^2(K_{\mathrm{assoc}}c_4+K_{\mathrm{assoc}}c_5+1)^2-4K_{\mathrm{assoc}}{}^2c_4c_5\mathrm{L}^2}}{2K_{\mathrm{assoc}}c_5}, \qquad (1)$$

where c_4 and c_5 denote the total concentration of host and guest, respectively, and L represents ΔA after complete encapsulation of the guest. This analysis leads to a $K_{\mathrm{assoc}} \approx 7 \cdot 10^8$ M^{-1}. We note, however, that owing to the high value of $K_{\mathrm{assoc}} \cdot c_5$ (= 3,975), there is a large error associated with this value (see References 7, 8).

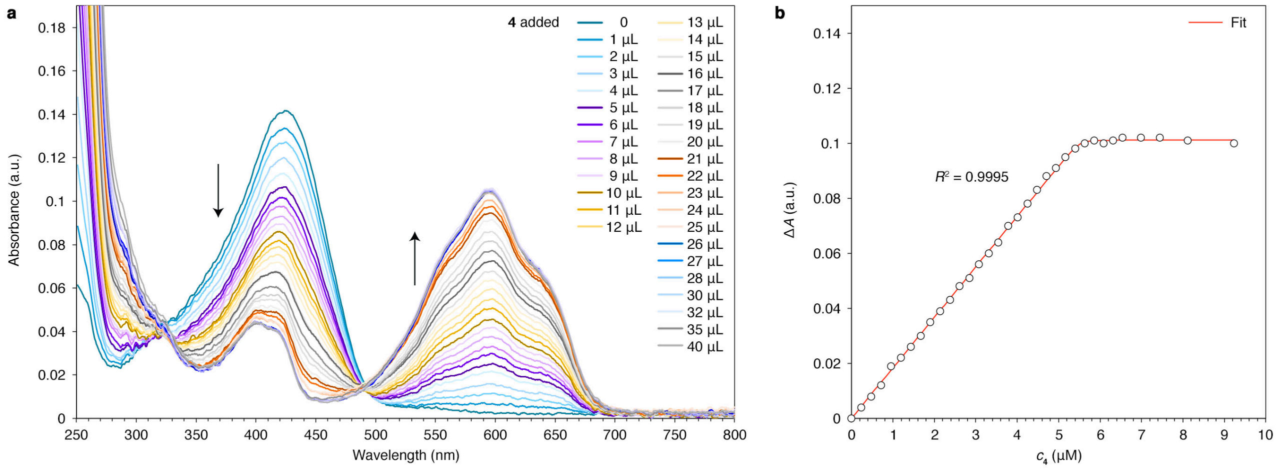

Supplementary Figure 22 | Changes in **a**, the UV/Vis spectra, and **b**, ΔA at 592 nm during titration of **5** with **4**.

Supplementary Note 10 | Analysis of water molecules associated with **4**.

We hypothesize that the encapsulation of spiropyrans by cage **4** is facilitated by the release of high-energy water[9,10] from the cage's cavity. The following observations point to the presence of water in the crystal structure and/or in the cavity of **4**.

- First, refinement of the X-ray crystal structure of **4** allows us to model 17 water molecules per cage (see the CIF file), four of which reside in the cavity (Supplementary Fig. 23a). Notably, water molecules were also resolved—as an adamantane-like cluster comprising ten water molecules—inside the cavity of a cage similar to **2** (with 2,2′-bipyridine rather than tmeda as the blocking ligand)[11].
- Second, we performed atomistic molecular dynamics simulation of **4** in explicit water at room temperature (see Methods in the main text). Supplementary Fig. 23b shows a representative snapshot of the system, where we highlighted water molecules and nitrates residing within the cage's cavity, approximated by a sphere centered at the geometrical center of the six Pd ions and with a radius of 5.83 Å equal to the average distance from the cage's center to the benzene rings of the cage walls. This volume (0.832 nm^3) corresponds to 20 water molecules (see Supplementary Fig. 23c, dashed line). The average water density in the cavity (calculated from the distribution of the oxygen atoms) 0.723 $g \cdot cm^{-3}$. All the numbers were averaged over 600 frames (the last 3 ns) of the simulations.
- Third, we performed thermogravimetric analysis (TGA) of **4** recrystallized from water. Mass decrease in the temperature range between r.t. and ~150 °C can be attributed to the loss of water[12]. We found a mass decrease of ~13% (Supplementary Fig. 23e), which corresponds to 26 water molecules per cage.

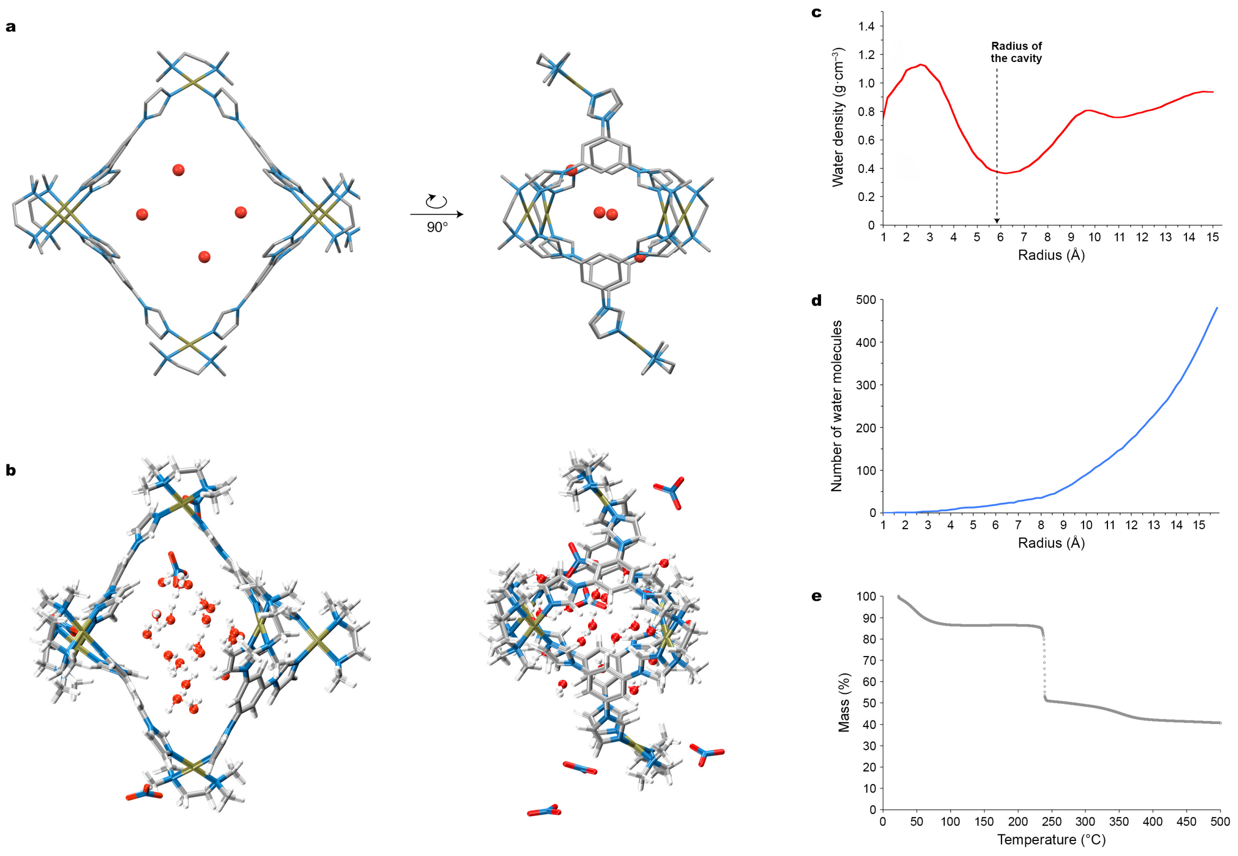

Supplementary Figure 23 | a, X-ray structure of cage **4** showing four ordered water molecules residing in specific positions in the cage's cavity (hydrogen atoms omitted for clarity). **b**, Two views of a snapshot from atomistic simulations of **4** in water. Pd, yellow; C gray; N, blue; O, red; H, white. **c**, Distribution of water density as a function of the distance from the center of the cavity. **d**, Cumulative number of water molecules contained within concentric spheres centered at the center of the cavity of the cage. **e**, Thermogravimetric analysis (TGA) of solid **4**.

Supplementary Note 11 | Analysis of guest-induced deformation of **4**.

The X-ray crystal structure of **4** was previously reported for a 1:1 *exo* complex of **4** and small molecule 4,5-dichloro-3,6-dioxocyclohexa-1,4-diene-1,2-bis(olate) (CCDC accession code 867175; HAZLAX)[2]. This complex formed upon co-crystallization of the two compounds, and the resulting co-crystal featured molecules of 4,5-dichloro-3,6-dioxocyclohexa-1,4-diene-1,2-bis(olate) placed between unfilled cages **4**.[2] The unfilled cage **4** extracted from this crystal structure is shown in red in Supplementary Fig. 24a, where it is superimposed with our **4** (shown in green). These two structures superimpose very well, with a root-mean-square (RMS) overlay of 0.432.

To determine the degree of structural deformation of cage **4** induced by guest **5**, we superimposed the X-ray structures of **4** and **5**⊂**4**. As Supplementary Fig. 24b shows, **4** undergoes a pronounced structural change upon binding **5**, with a RMS overlay of these two structures = 1.12 (maximal displacement = 2.38 Å). We also compared the structure of **5**⊂**4** with that of the previously reported inclusion complex (pyrene-1-carbaldehyde)$_2$⊂**4** (CCDC accession code 867176; HAZLEB)[2]. These two complexes are shown in Supplementary Fig. 24c. Interestingly, despite binding very different guests and the resulting inclusion complexes being of different stoichiometries (1:1 and 1:2), there is a good fit between the structures of **4** in these two complexes (RMS overlay = 0.62).

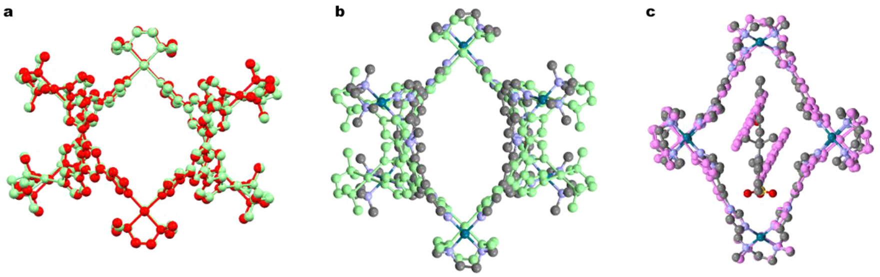

Supplementary Figure 24 | Comparison of different X-ray structures of **4**. **a**, Green: cage **4**; red: cage **4** from a co-crystal with 4,5-dichloro-3,6-dioxocyclohexa-1,4-diene-1,2-bis(olate)[2] (guest not shown for clarity). **b**, Green: cage **4**; colored by elements: inclusion complex **5**⊂**4** (guest not shown for clarity). **c**, Colored by elements: inclusion complex **5**⊂**4**; purple: inclusion complex[2] (pyrene-1-carbaldehyde)$_2$⊂**4**.

Supplementary Note 12 | Characterization of inclusion complex **6**⊂**4**.

Inclusion complex **6**⊂**4** was obtained in ~50% yield.

^{1}**H NMR** (800 MHz, D_2O): δ = 9.15 (br, 8H, **4**$_{4}$), 8.82 (s, 4H, **4**$_{1}$), 7.90–7.56 (m, 36H, **4**$_{2+3+5+6+7+8}$), 6.82 (d, ^{3}J = 7.0 Hz, 1H, **6**$_{o}$), 6.73 (t, ^{3}J = 7.0 Hz, 1H, **6**$_{n}$), 6.27 (t, ^{3}J = 7.2 Hz, 1H, **6**$_{d}$), 6.05 (t, ^{3}J = 7.2 Hz, 1H, **6**$_{c}$), 6.00 (d, ^{3}J = 9.9 Hz, 1H, **6**$_{j}$), 5.76 (d, ^{3}J = 6.9 Hz, 1H, **6**$_{e}$), 4.98 (d, ^{3}J = 7.5 Hz, 1H, **6**$_{l}$), 4.94 (t, ^{3}J = 6.5 Hz, 1H, **6**$_{m}$), 4.60 (d, ^{3}J = 7.5 Hz, 1H, **6**$_{b}$), 4.46 (d, ^{3}J = 9.9 Hz, 1H, **6**$_{i}$), 3.08 (s, 12H, **4**$_{CH2}$), 3.06 (s, 12H, **4**$_{CH2}$), 2.73 (s, 24H, **4**$_{CH3}$), 2.71 (s, 24H, **4**$_{CH3}$), 2.67 (s, 24H, **4**$_{CH3}$), 0.44 (s, 3H, **6**$_{s}$), –0.62 (s, 3H, **6**$_{q/r}$), –0.68 (s, 3H, **6**$_{q/r}$). ^{13}**C NMR** (200 MHz, D_2O): δ = 152.86 (**6**$_{p}$), 145.47 (**6**$_{a}$), 138.01 (**4**$_{9}$), 137.61 (**4**$_{10}$), 137.44 (**4**$_{4}$), 136.82 (**4**$_{1}$), 135.10 (**6**$_{f}$), 128.88 (**4**$_{7+8}$), 128.61 (**6**$_{j}$), 127.83 (**6**$_{m}$), 127.64 (**6**$_{c}$), 127.23 (**6**$_{o}$), 120.88 (**6**$_{n}$), 120.56 (**4**$_{3+6}$), 119.90 (**6**$_{e}$), 119.54 (**6**$_{d}$), 118.77 (**6**$_{k}$), 115.93 (**6**$_{i}$), 114.37 (**4**$_{2}$), 112.64 (**6**$_{l}$), 112.50 (**4**$_{5}$), 105.44 (**6**$_{b}$), 102.13 (**6**$_{h}$), 62.47 (**4**$_{CH2}$), 50.24 (**4**$_{CH3}$), 50.15 (**4**$_{CH3}$), 49.95 (**4**$_{CH3}$), 47.75 (**6**$_{g}$), 26.64 (**6**$_{s}$), 22.61 (**6**$_{q/r}$), 16.79 (**6**$_{q/r}$). ^{1}**H-DOSY NMR** (800 MHz, D_2O, 298 K): D = 0.19 (± 0.01) × 10^{-5} cm^2/s. **Elemental analysis**: calcd: C, 38.12; H, 4.66; N, 20.44; found: 37.91; H, 4.78; N, 20.47.

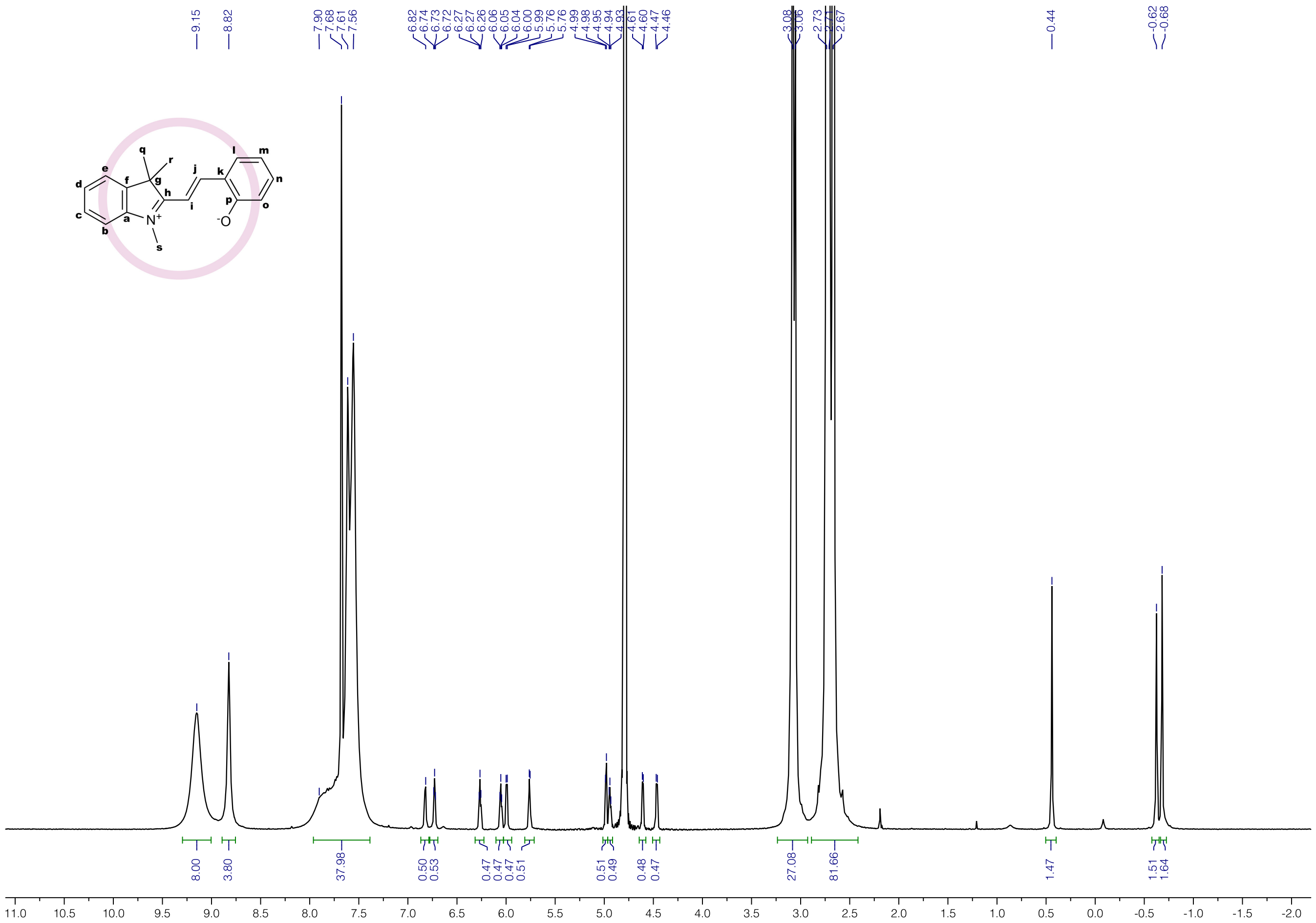

Supplementary Figure 25 | ^{1}H NMR spectrum of **6**⊂**4** (800 MHz, D_2O). For signal assignment, see Supplementary Note 12.

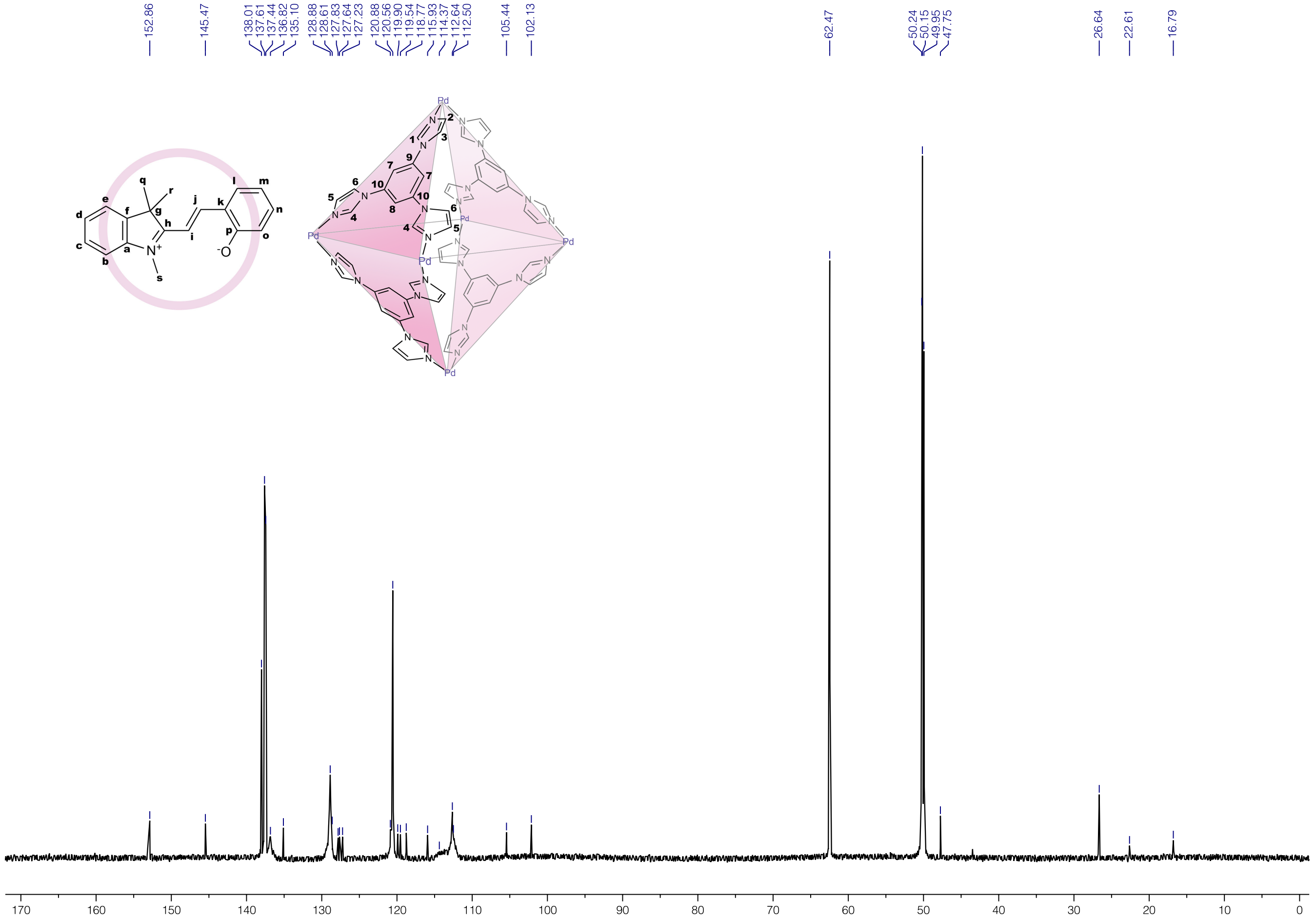

Supplementary Figure 26 | ^{13}C NMR spectrum of **6⊂4** (after baseline correction) (200 MHz, D_2O). For signal assignment, see Supplementary Note 12.

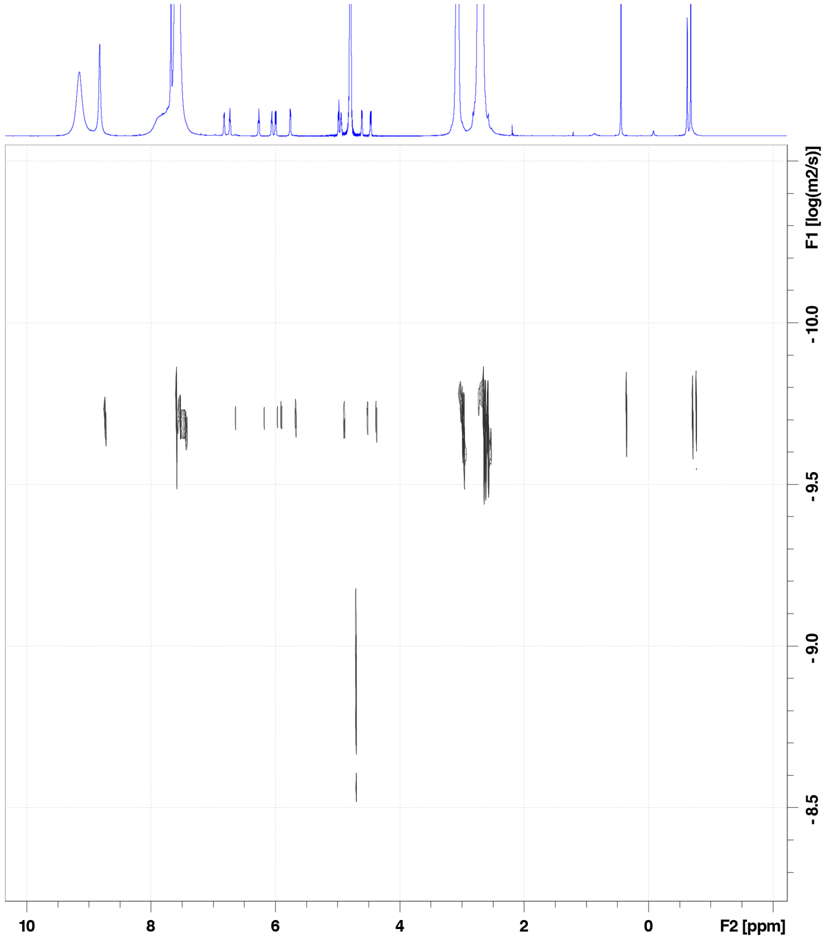

Supplementary Figure 27 | ^{1}H-DOSY NMR spectrum of **6⊂4** (800 MHz, D_2O).

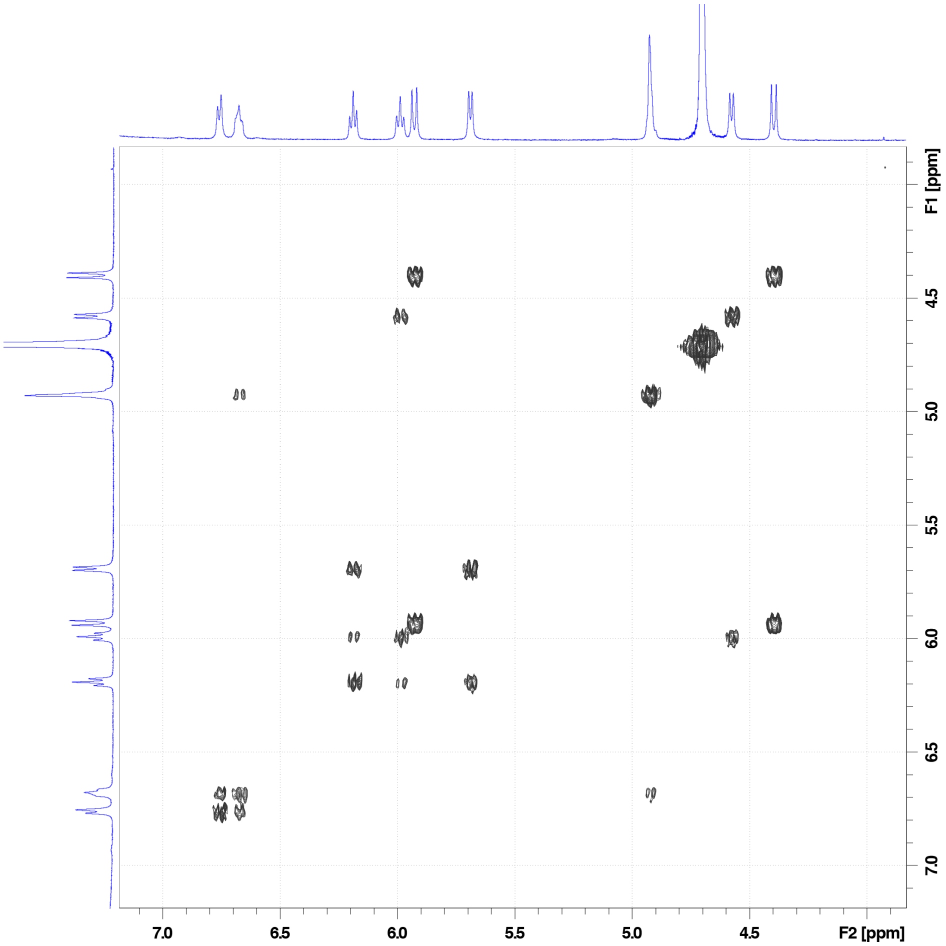

Supplementary Figure 28 | Partial ^{1}H-^{1}H COSY NMR spectrum of **6**⊂**4** (500 MHz, D_2O).

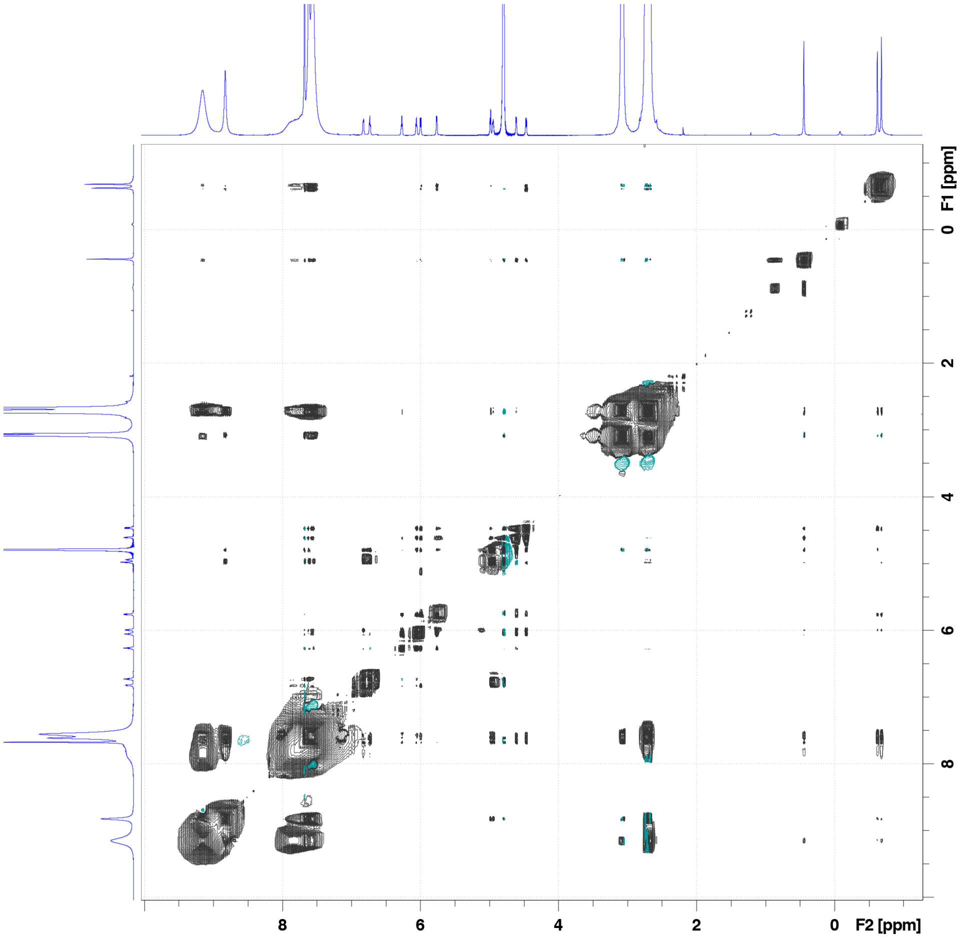

Supplementary Figure 29 | ^{1}H-^{1}H NOESY NMR spectrum of **6⊂4** (800 MHz, D_2O).

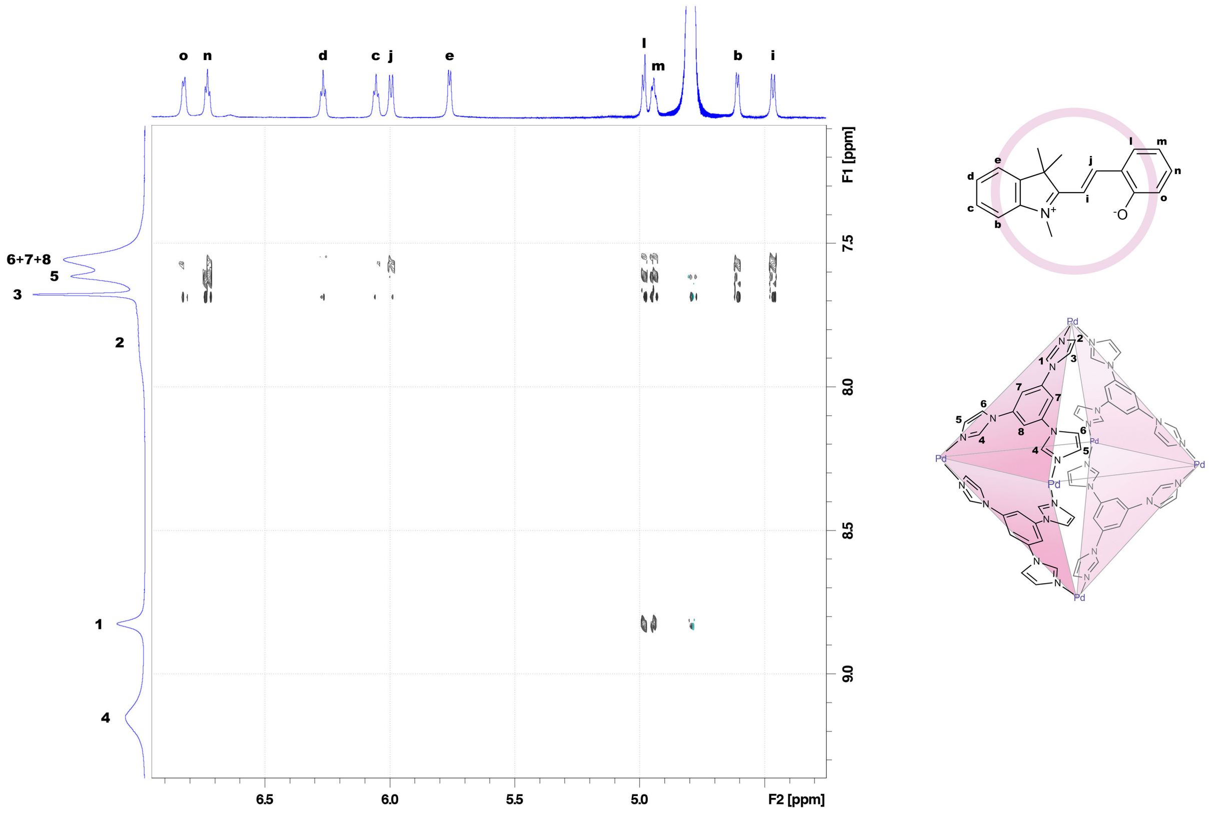

Supplementary Figure 30 | Partial ^{1}H-^{1}H NOESY NMR spectrum of **6⊂4** showing host-guest correlations (800 MHz, D_2O) (the corresponding full-range spectrum is shown in Supplementary Fig. 29).

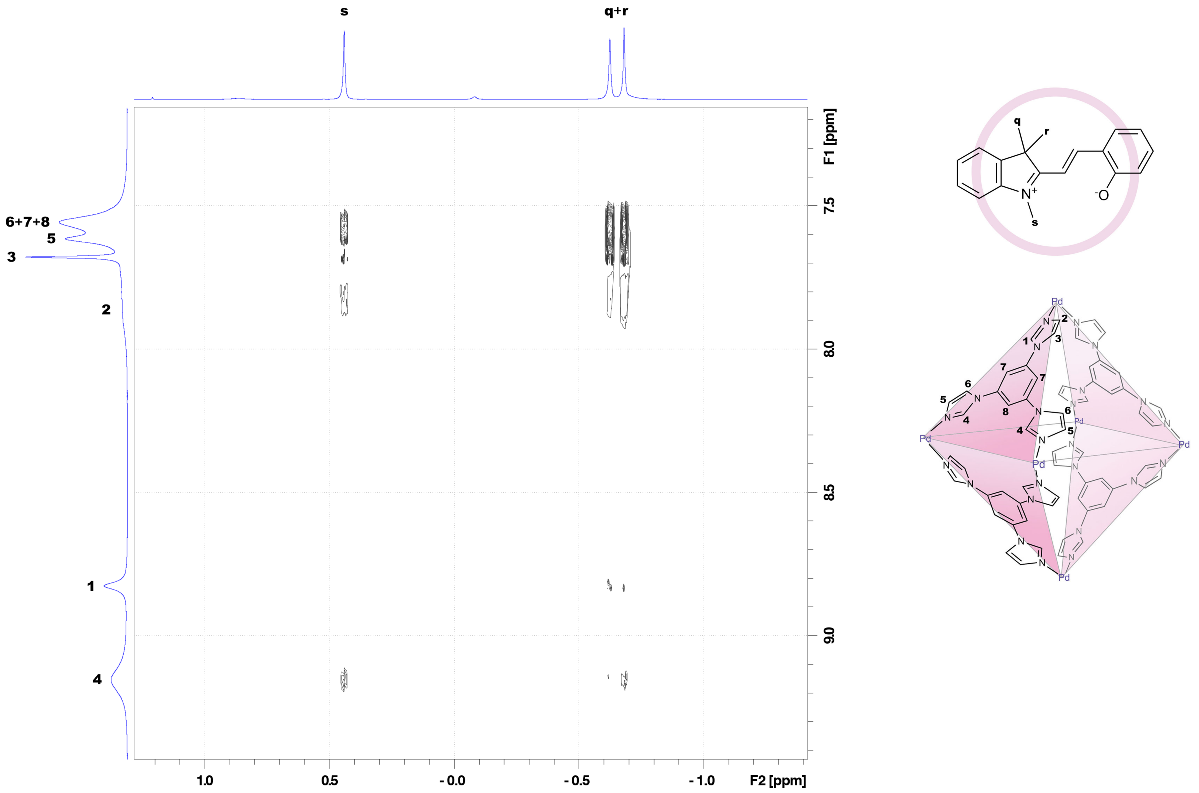

Supplementary Figure 31 | Partial ^{1}H-^{1}H NOESY NMR spectrum of **6⊂4** showing host-guest correlations (800 MHz, D_2O) (the corresponding full-range spectrum is shown in Supplementary Fig. 29).

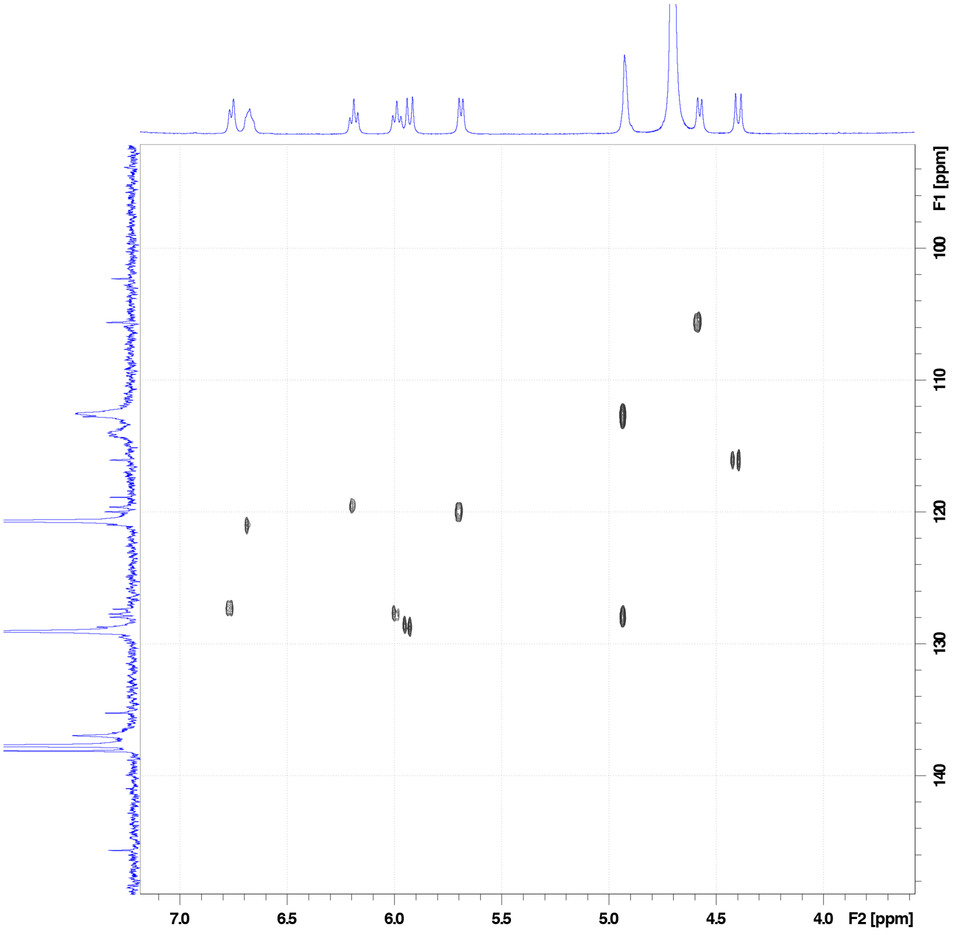

Supplementary Figure 32 | Partial ^{1}H-^{13}C HSQC NMR spectrum of **6**⊂**4** (500 MHz, D_2O).

Supplementary Note 13 | Characterization of inclusion complex **8⊂4**.

Inclusion complex **8⊂4** was obtained in ~70% yield.

^{1}H NMR (500 MHz, D_2O): δ = 9.24 (br, 8H, $\mathbf{4}_{\mathbf{4}}$), 8.94 (br, 4H, $\mathbf{4}_{\mathbf{1}}$), 7.97 (br, 4H, $\mathbf{4}_{\mathbf{5}}$), 7.77 (br, 4H, $\mathbf{4}_{\mathbf{3}}$), 7.68 (br, 8H, $\mathbf{4}_{\mathbf{2+5}}$), 7.64 (br, 12H, $\mathbf{4}_{\mathbf{7+8}}$), 7.49 (br, 4H, $\mathbf{4}_{\mathbf{6}}$), 7.37 (br, 4H, $\mathbf{4}_{\mathbf{6}}$), 6.87 (m, 2H, $\mathbf{8}_{\mathbf{m+n}}$), 6.35 (d, ^3J = 10.0 Hz, 1H, $\mathbf{8}_{\mathbf{j}}$), 6.18 (t, ^3J = 7.6 Hz, 1H, $\mathbf{8}_{\mathbf{d}}$), 5.80 (d, ^3J = 7.2 Hz, 1H, $\mathbf{8}_{\mathbf{e}}$), 5.76 (d, ^3J = 6.9 Hz, 1H, $\mathbf{8}_{\mathbf{l}}$), 5.59 (t, ^3J = 7.6 Hz, 1H, $\mathbf{8}_{\mathbf{c}}$), 4.81 (d, ^3J = 10.0 Hz, 1H, $\mathbf{8}_{\mathbf{i}}$), 4.49 (d, ^3J = 7.6 Hz, 1H, $\mathbf{8}_{\mathbf{b}}$), 3.12 (br, 16H, $\mathbf{4}_{CH2}$), 3.10 (br, 8H, $\mathbf{4}_{CH2}$), 2.76 (br, 24H, $\mathbf{4}_{CH3}$), 2.75 (br, 24H, $\mathbf{4}_{CH3}$), 2.70 (br, 24H, $\mathbf{4}_{CH3}$), 0.32 (s, 3H, $\mathbf{8}_{\mathbf{s}}$), 0.02 (s, 3H, $\mathbf{8}_{\mathbf{t}}$), –0.56 (s, 3H, $\mathbf{8}_{\mathbf{q/r}}$), –0.66 (s, 3H, $\mathbf{8}_{\mathbf{q/r}}$). **^{13}C NMR** (125 MHz, D_2O): δ = 150.58 ($\mathbf{8}_{\mathbf{p}}$), 145.74 ($\mathbf{8}_{\mathbf{a}}$), 138.10 ($\mathbf{4}_{\mathbf{9}}$), 137.72 ($\mathbf{4}_{\mathbf{10}}$), 137.46 ($\mathbf{4}_{\mathbf{4}}$), 136.66 ($\mathbf{4}_{\mathbf{1}}$), 135.96 ($\mathbf{8}_{\mathbf{f}}$), 129.60 ($\mathbf{8}_{\mathbf{l}}$), 129.25 ($\mathbf{8}_{\mathbf{j}}$), 129.06 ($\mathbf{4}_{\mathbf{7+8}}$), 127.25 ($\mathbf{8}_{\mathbf{c}}$), 125.25 ($\mathbf{8}_{\mathbf{n}}$), 122.12 ($\mathbf{8}_{\mathbf{o}}$), 120.66 ($\mathbf{4}_{\mathbf{6+6}}$), 120.59 ($\mathbf{4}_{\mathbf{3}}$), 120.54 ($\mathbf{8}_{\mathbf{m}}$), 119.61 ($\mathbf{8}_{\mathbf{e}}$), 119.05 ($\mathbf{8}_{\mathbf{d}}$), 118.96 ($\mathbf{8}_{\mathbf{k}}$), 115.80 ($\mathbf{8}_{\mathbf{i}}$), 113.82 ($\mathbf{4}_{\mathbf{5}}$), 112.99 ($\mathbf{4}_{\mathbf{2}}$), 111.86 ($\mathbf{4}_{\mathbf{5}}$), 104.84 ($\mathbf{8}_{\mathbf{b}}$), 101.29 ($\mathbf{8}_{\mathbf{h}}$), 62.56 ($\mathbf{4}_{CH2}$), 50.32 ($\mathbf{4}_{CH3}$), 50.22 ($\mathbf{4}_{CH3}$), 50.01 ($\mathbf{4}_{CH3}$), 47.09 ($\mathbf{8}_{\mathbf{g}}$), 26.34 ($\mathbf{8}_{\mathbf{s}}$), 22.52 ($\mathbf{8}_{\mathbf{q/r}}$), 17.01 ($\mathbf{8}_{\mathbf{q/r}}$), 13.56 ($\mathbf{8}_{\mathbf{t}}$). **^{1}H-DOSY NMR** (500 MHz, D_2O, 298 K): D = 0.19 (± 0.01) × 10^{-5} cm^2/s. **Elemental analysis**: calcd: C, 38.98; H, 4.72; N, 20.13; found: 38.84; H, 4.74; N, 20.05.

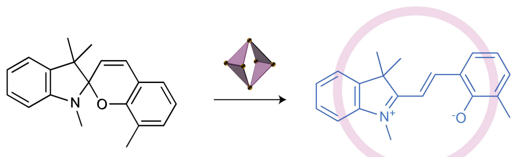

$\mathbf{8}_{SP}$

$\mathbf{8}_{MC}$**⊂4**

Supplementary Figure 33 | Cage-induced ring-opening isomerization of **8**.

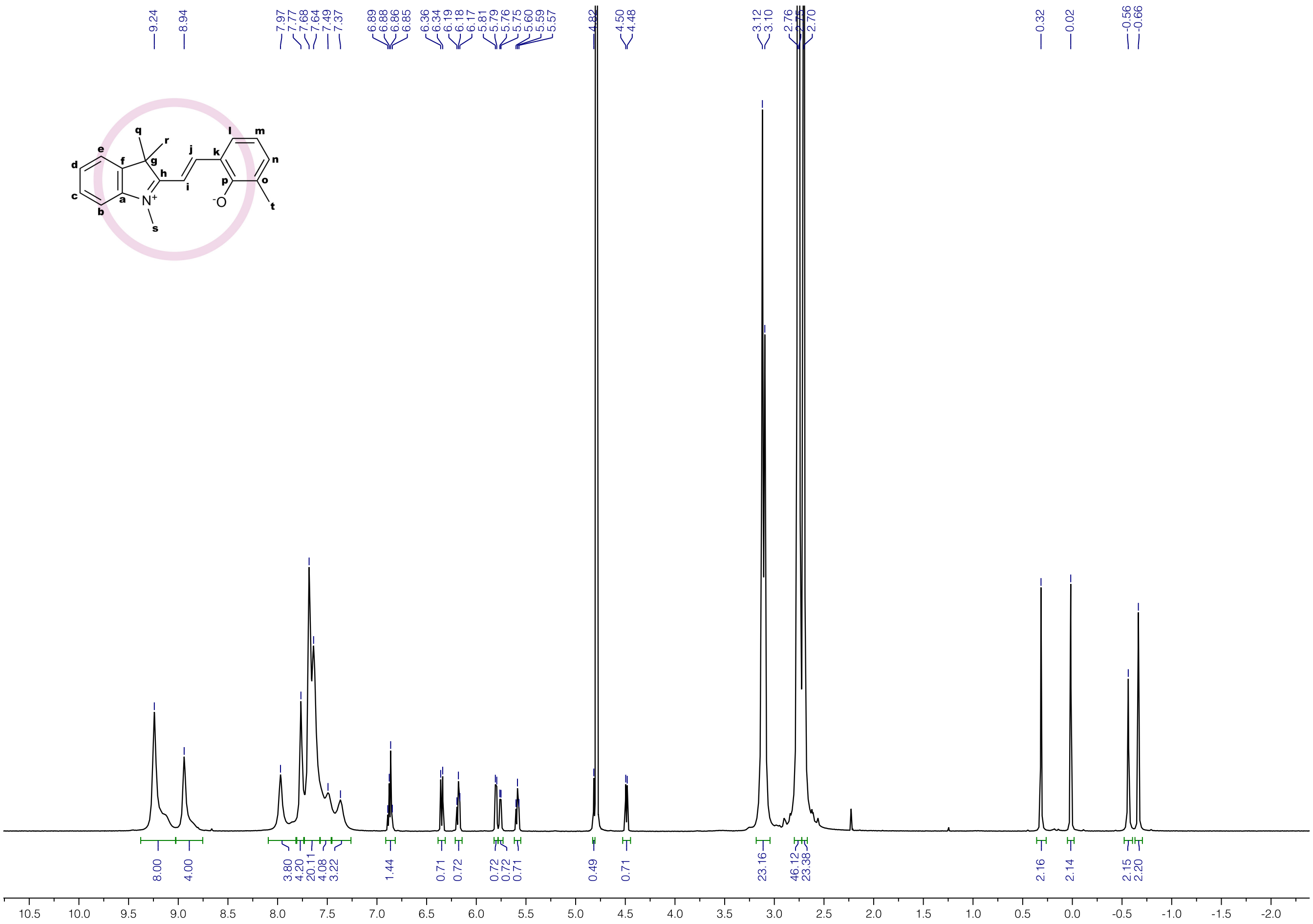

Supplementary Figure 34 | ^{1}H NMR spectrum of **8⊂4** (500 MHz, D_2O). For signal assignment, see Supplementary Note 13.

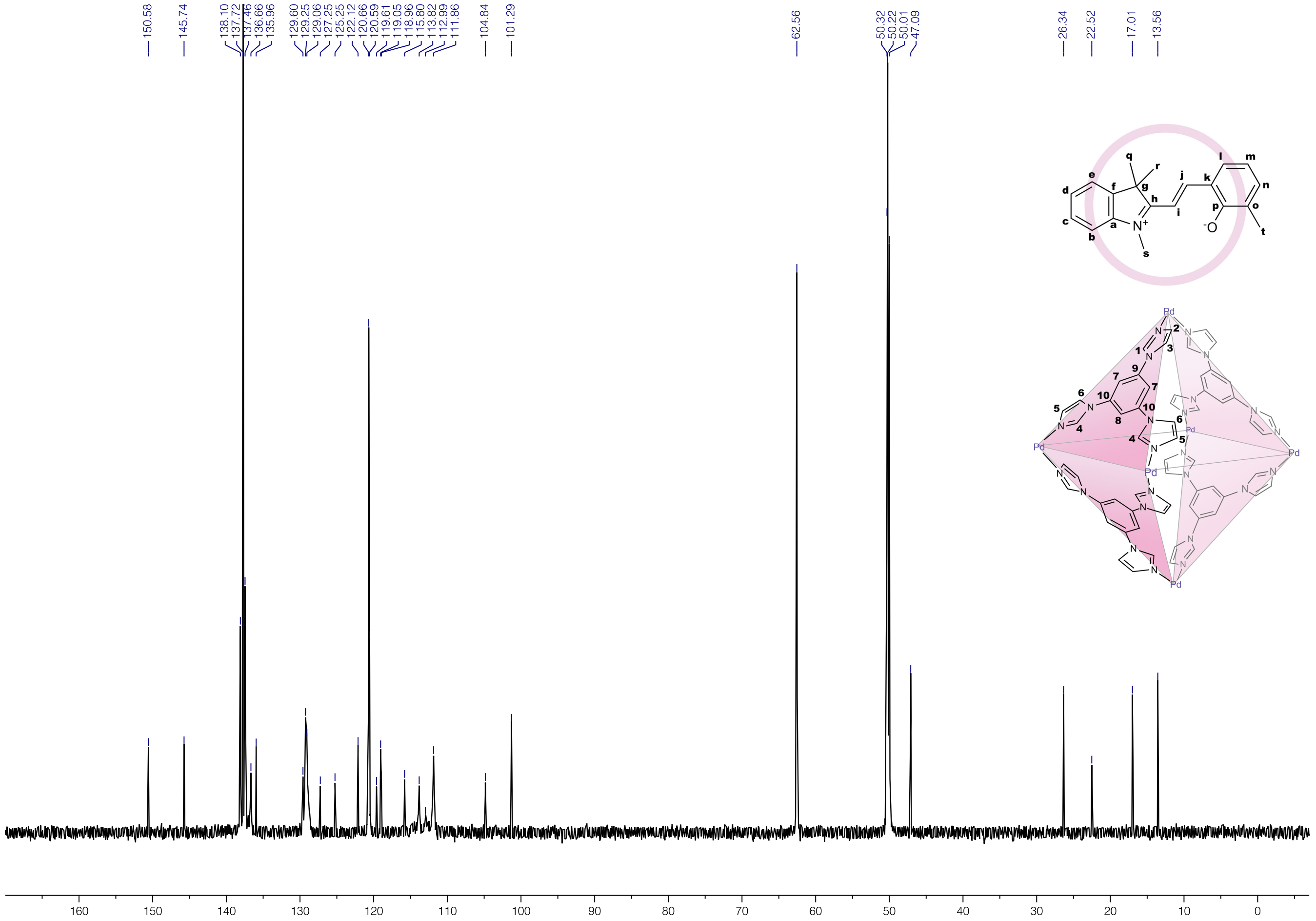

Supplementary Figure 35 | ^{13}C NMR spectrum of **8⊂4** (125 MHz, D_2O). For signal assignment, see Supplementary Note 12.

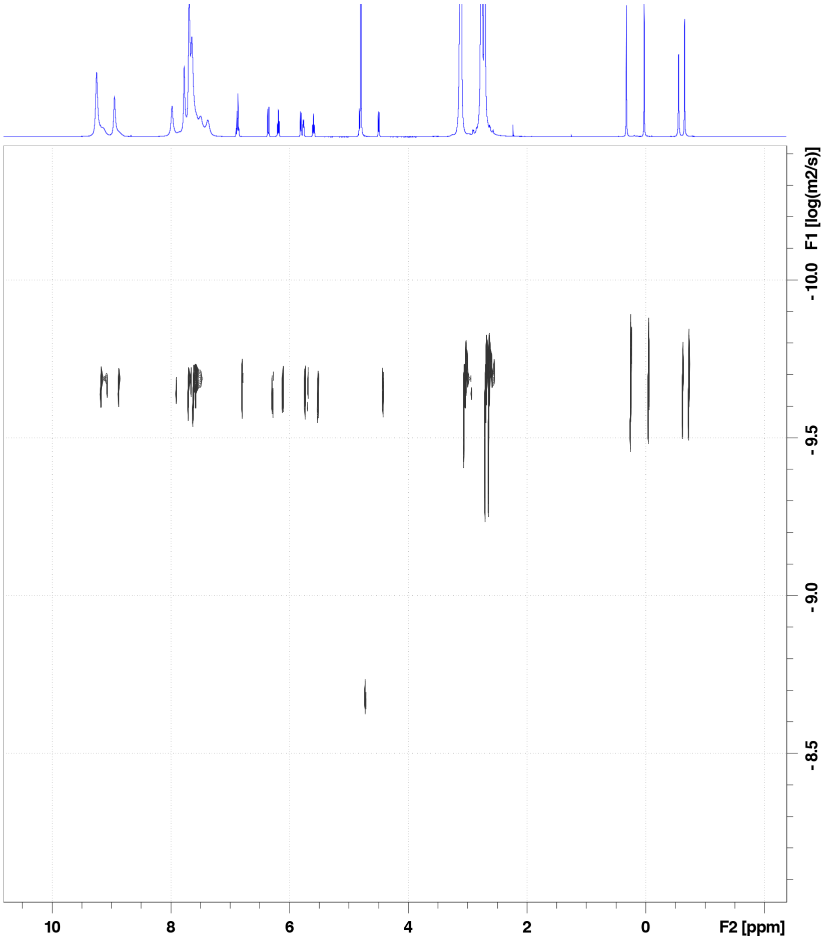

Supplementary Figure 36 | ^{1}H-DOSY NMR spectrum of **8⊂4** (500 MHz, D_2O).

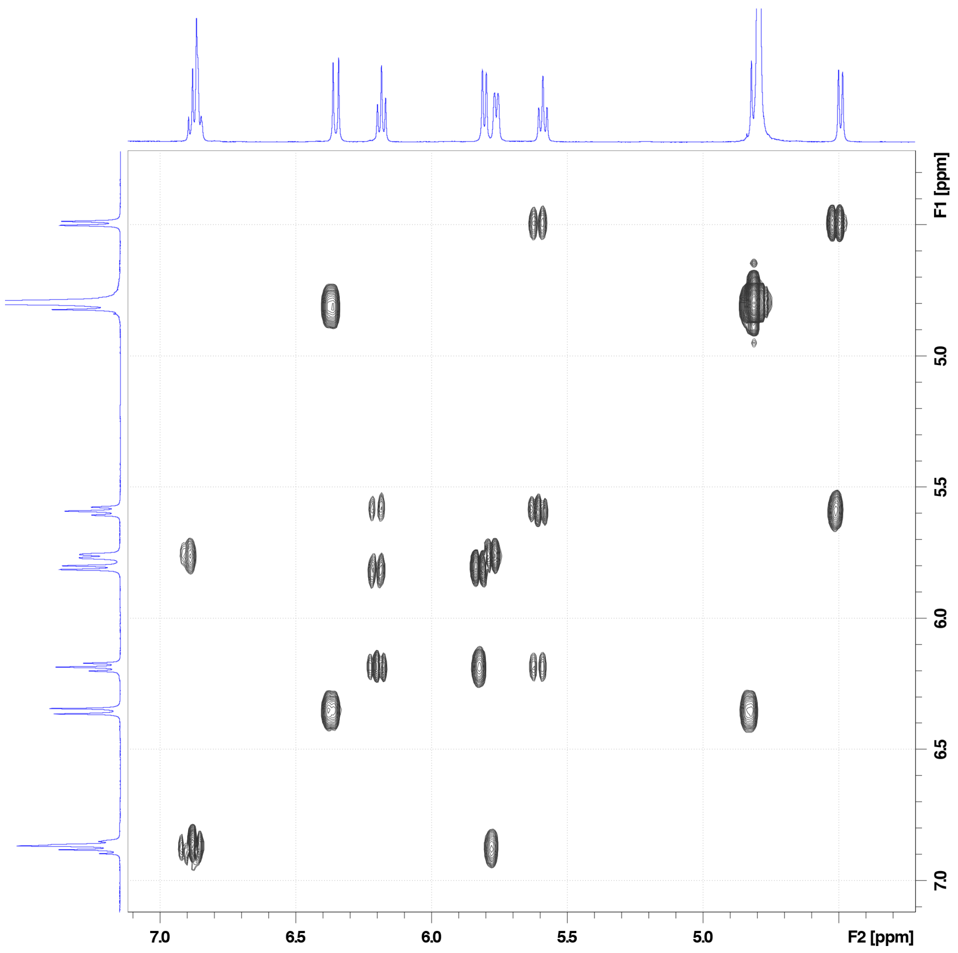

Supplementary Figure 37 | Partial ^{1}H-^{1}H COSY NMR spectrum of **8⊂4** (500 MHz, D_2O).

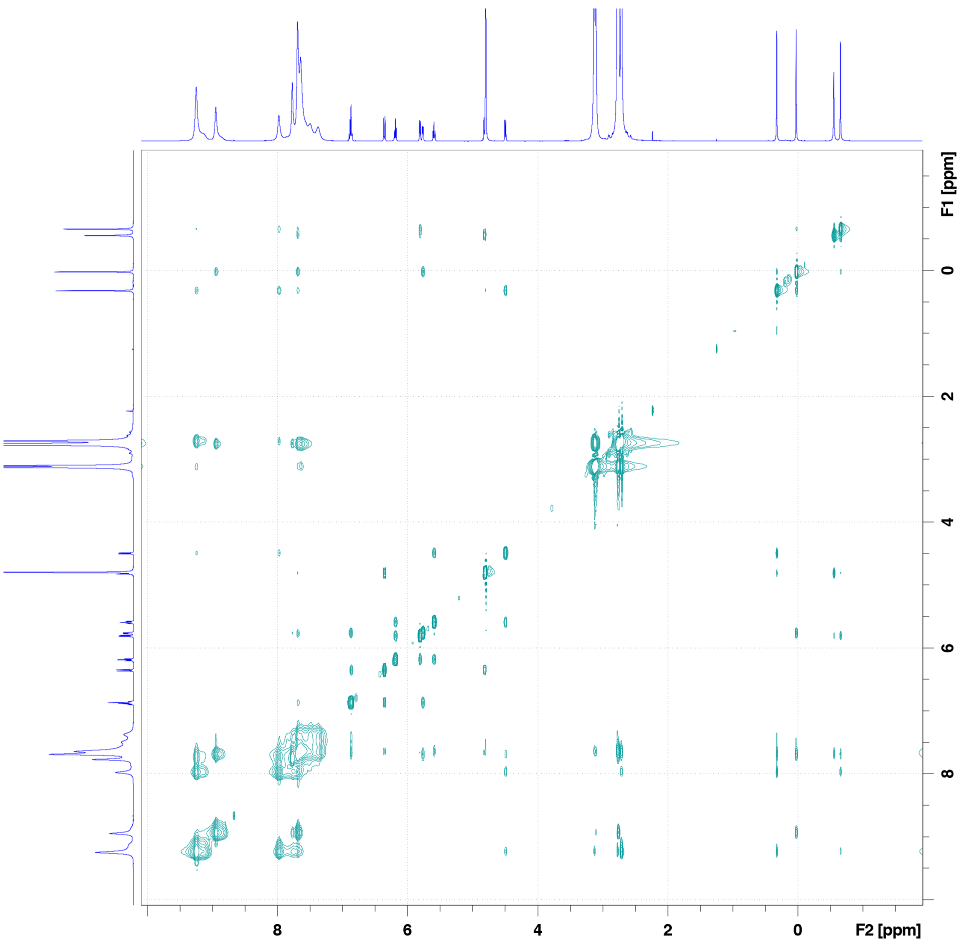

Supplementary Figure 38 | ^{1}H-^{1}H NOESY NMR spectrum of **8⊂4** (500 MHz, D_2O).

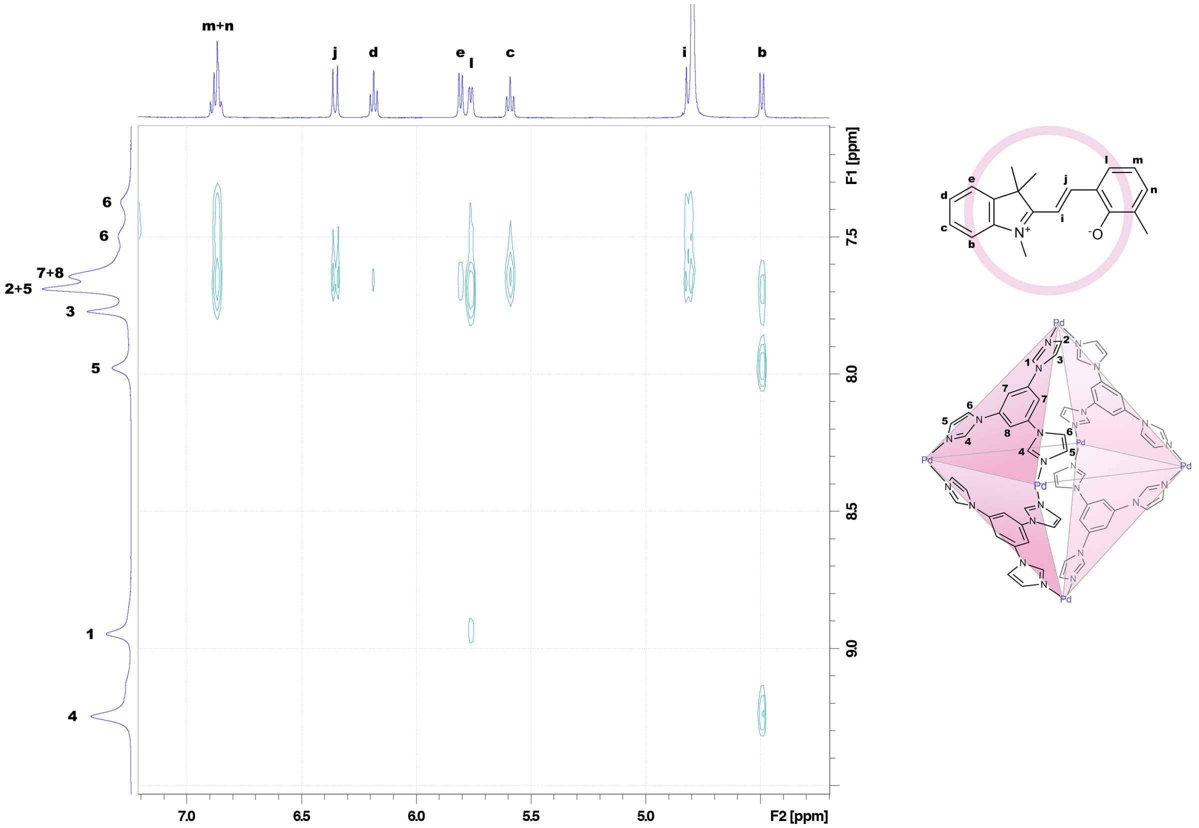

Supplementary Figure 39 | Partial ^{1}H-^{1}H NOESY NMR spectrum of **8⊂4** showing host-guest correlations (500 MHz, D_2O) (the corresponding full-range spectrum is shown in Supplementary Fig. 38).

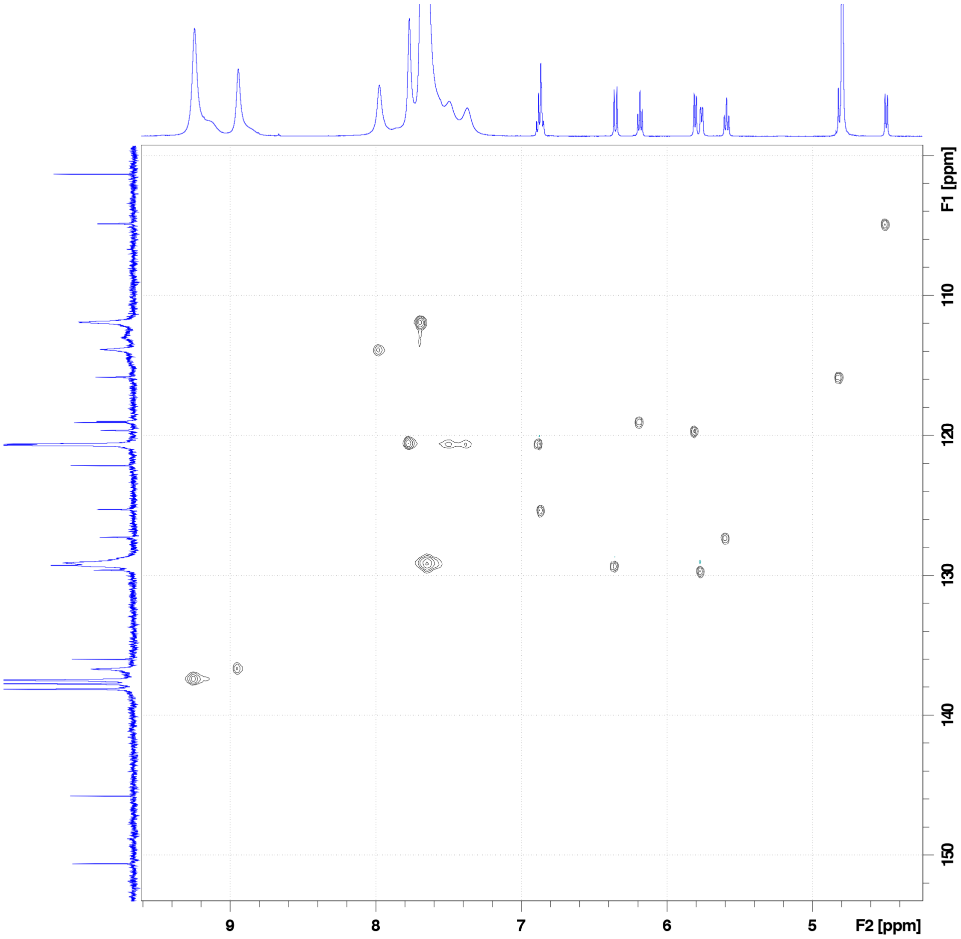

Supplementary Figure 40 | Partial ^{1}H-^{13}C HSQC NMR spectrum of **8⊂4** (500 MHz, D_2O).

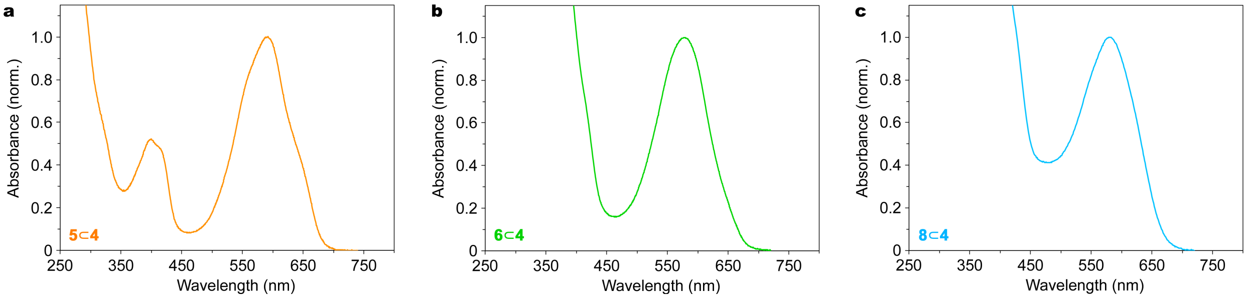

Supplementary Figure 41 | Normalized UV/Vis absorption spectra of dilute solutions of **5⊂4**, **6⊂4**, and **8⊂4**. Note the fine structure of **5⊂4**'s main absorption band (~600 nm).

Supplementary Note 14 | Fluorescent properties of **5⊂4**.

The open (MC) form of spiropyran is known to exhibit red fluorescence[13,14]. Exposing a dilute aqueous solution of **4**-encapsulated **5** to blue light (which acts both as an excitation source and a stimulus, inducing the ring-closing isomerization) resulted in the appearance of red fluorescence, which quickly disappeared as a result of the ring-closing reaction. Emission spectrum after exposing the solution to blue light is shown as the gray trace in Supplementary Fig. 42.

Working with more concentrated (~10 mg/mL) solutions of **5⊂4**, we observed beautiful patterns of red and green fluorescence (examples shown in Supplementary Fig. 43). In these experiments we placed a solution of the inclusion complex in H_2O in an NMR tube and irradiated it with blue (460 nm) LED through an optical fiber (thickness = 1 mm). Because of the high concentration of the solution and the relatively low intensity of the LED, only a thin layer of the solution turned colorless. Red emission due to MC was observed within a thin layer at the boundary between the photoisomerized (i.e., colorless; SP) and the unaffected (deeply colored; MC/MCH^+) portion of the solution due to the high optical density of – and limited light penetration throughout – the concentrated solution.

Interestingly, green fluorescence was observed close to the tip of the optical fiber. Within this region, the ring-closing isomerization took place and the majority of **5** was in the SP form. Green fluorescence of the SP isomer is typically not observed, however, it can be seen upon constraining the SP units. For example, Liu *et al*. observed green emission of SP upon confining it within polymer nanoparticles.[15] In our case, confining is achieved upon encapsulating spiropyran **5** within cage **4**. Indeed, no fluorescence was observed upon exciting a methanolic solution of unsubstituted spiropyran **6** with light at λ = 460 nm.

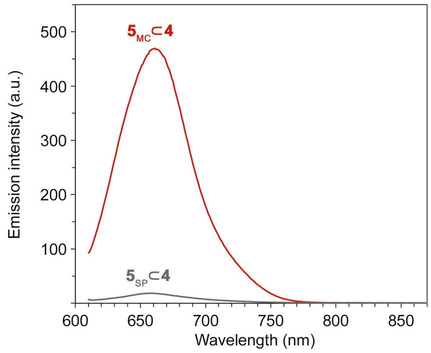

Supplementary Figure 42 | Emission spectra of a dilute solution of **5⊂4** in water before (red) and after (gray) exposure to blue light.

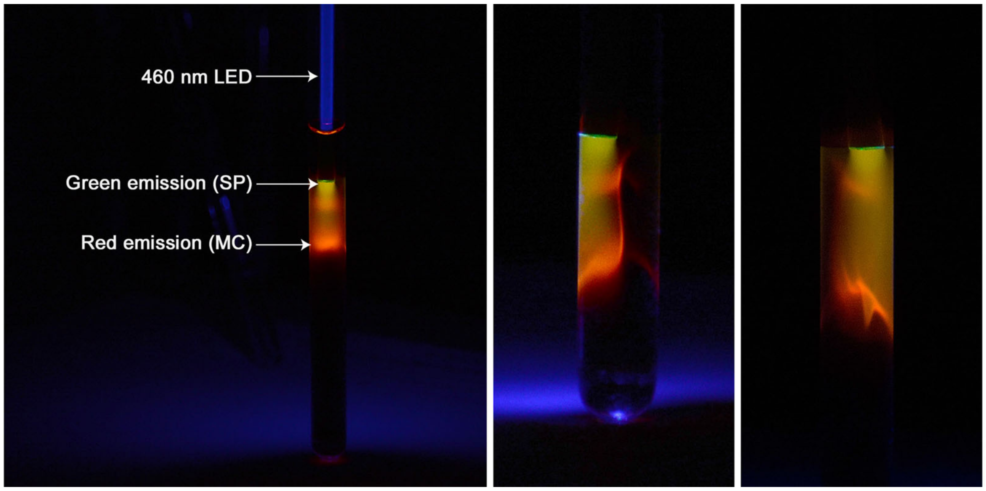

Supplementary Figure 43 | Photographs of an aqueous solution of **5⊂4** irradiated with a 460 nm LED.

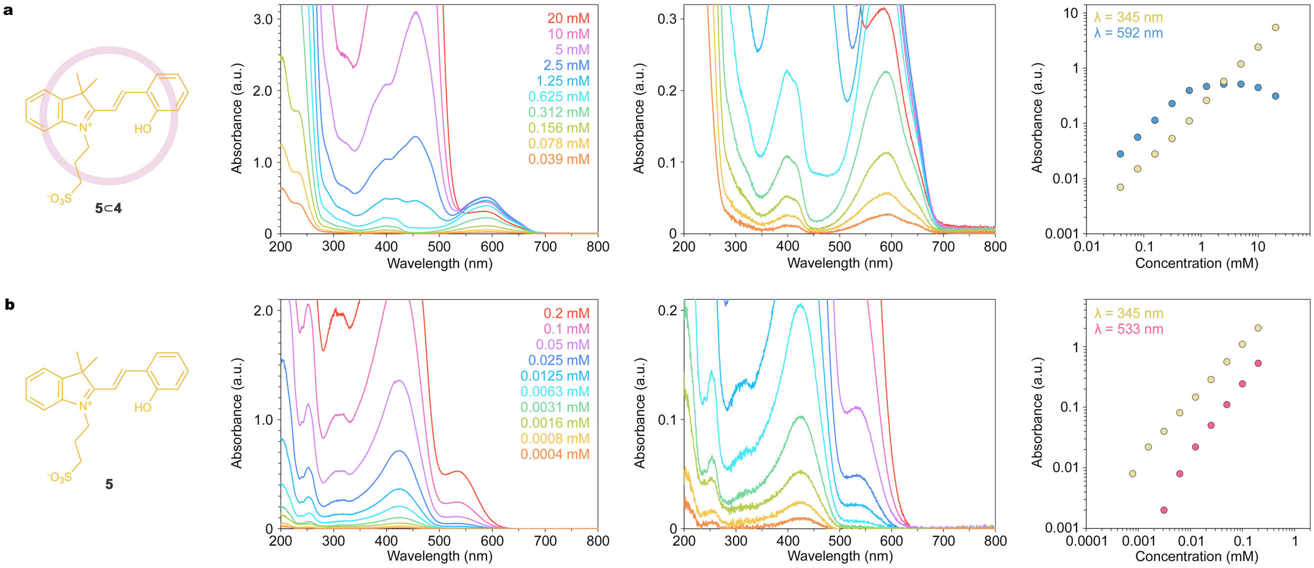

Supplementary Figure 44 | Cage-induced, concentration-dependent optical properties of spiropyran 5. a, A series of UV/Vis spectra of **5⊂4** in water at different concentrations of the inclusion complex. The initial spectrum (in red) was recorded for a solution of 67.3 mg (0.019 mmol) of **5⊂4** in 1.0 mL of pure water. Subsequent spectra were recorded after twofold dilutions. Cuvette with a 0.5 mm light path length was used. **b**, Lack of concentration-dependence of the optical properties of **4**-free **5**. The initial spectrum (in red) was recorded on a saturated solution of **5** in pure water (~0.2 mM). Subsequent spectra were recorded after twofold dilutions. Cuvette with a 10 mm light path length was used.

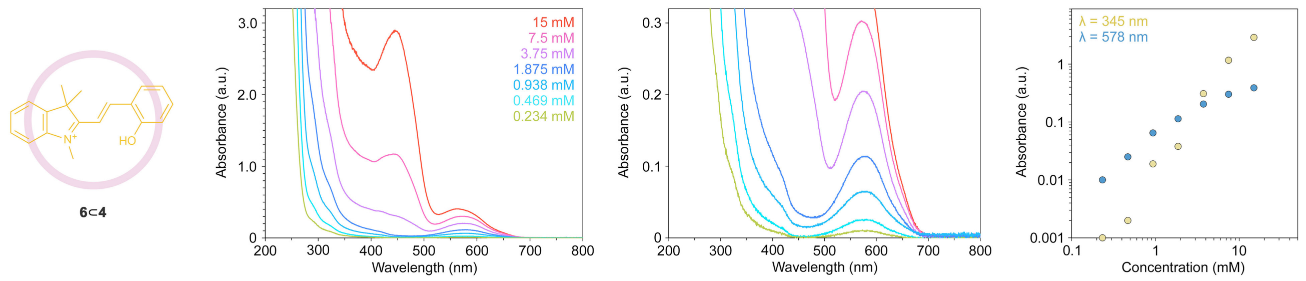

Supplementary Figure 45 | Cage-induced concentration-dependent optical properties of spiropyran 6. a, A series of UV/Vis spectra of **6⊂4** in water at different concentrations of the inclusion complex (cuvette with a 0.5 mm light path length was used). Note that the complex formed in a ~50% yield, i.e., approximately half of the cages remained empty.

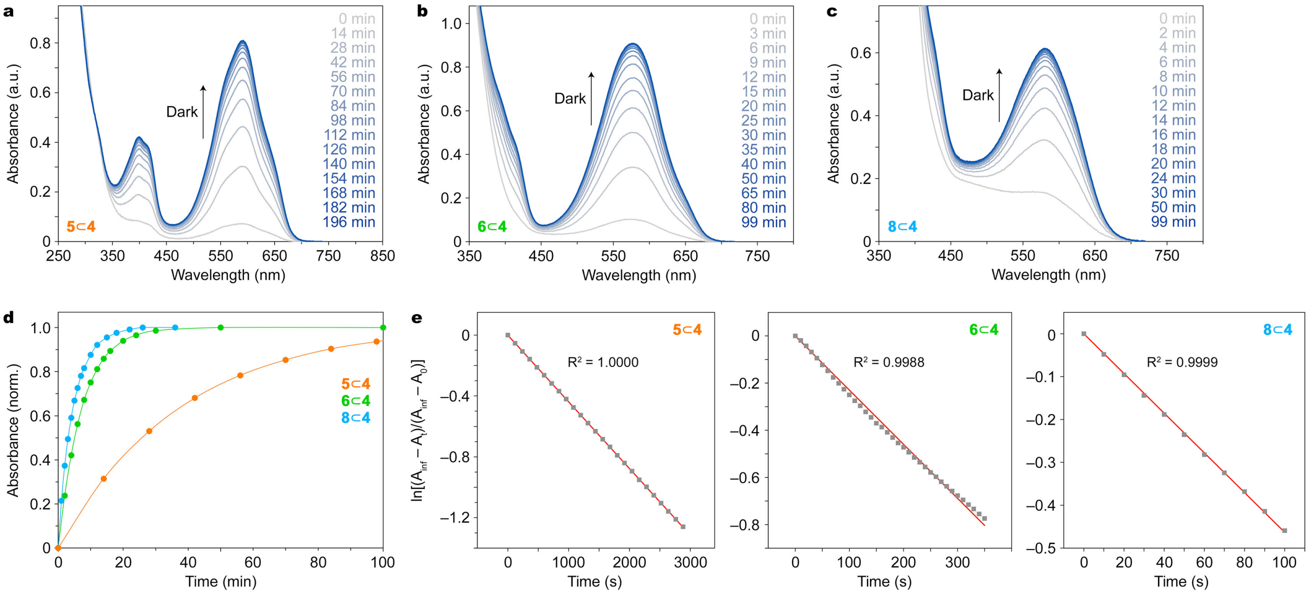

Supplementary Figure 46 | Photoisomerization and thermal relaxation of **5⊂4**, **6⊂4**, and **8⊂4**. Series of UV/Vis spectra following the spontaneous (dark) SP→MC ring-opening reaction occurring within **5⊂4** (**a**, replotted from Fig. 4d in the main text), **6⊂4** (**b**), and **8⊂4** (**c**). In all cases, the solutions were exposed to 460 nm light (1 min for **5⊂4**, 4 min for **6⊂4**, 8 min for **8⊂4**) and the resulting spectra are labeled as 0 min. Subsequent spectra were recorded at the times indicated. **d**, Normalized absorbance at the wavelength of maximum absorption for **5⊂4**, **6⊂4**, and **8⊂4** plotted as a function of time (wavelengths of maximum absorption: 592 nm for **5⊂4**, 578 nm for **6⊂4**, and 581 nm for **8⊂4**). **e**, Fitting of the experimental data derived from **d** (gray markers) to first-order kinetics. Time constants, $\tau_{1/2}$ = 26.3 min for **5⊂4**, $\tau_{1/2}$ = 5.0 min for **6⊂4**, and $\tau_{1/2}$ = 2.5 min for **8⊂4**.

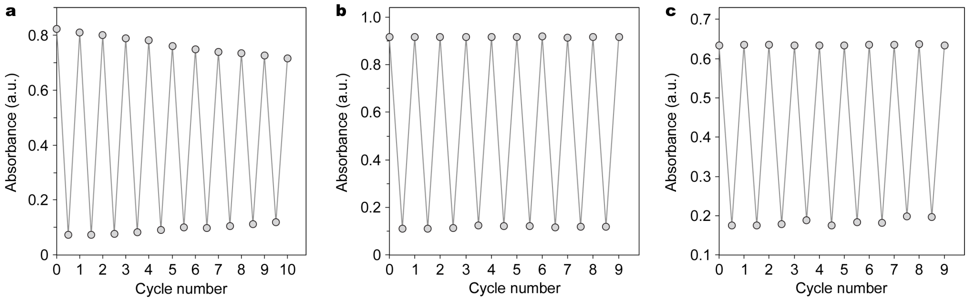

Supplementary Figure 47 | Reversible isomerization of encapsulated spiropyrans: **5⊂4** (**a**, replotted from Fig. 4e in the main text), **6⊂4** (**b**), and **8⊂4** (**c**). The y-axes show the absorbance at the wavelength of maximum absorption: 592 nm for **5⊂4**, 578 nm for **6⊂4**, and 581 nm for **8⊂4**. Each cycle comprised exposure to 460 nm LED (1 min for **5⊂4**, 4 min for **6⊂4**, and 8 min for **8⊂4**) followed by dark relaxation (200 min for **5⊂4**, 100 min for **6⊂4**, and 100 min for **8⊂4**).

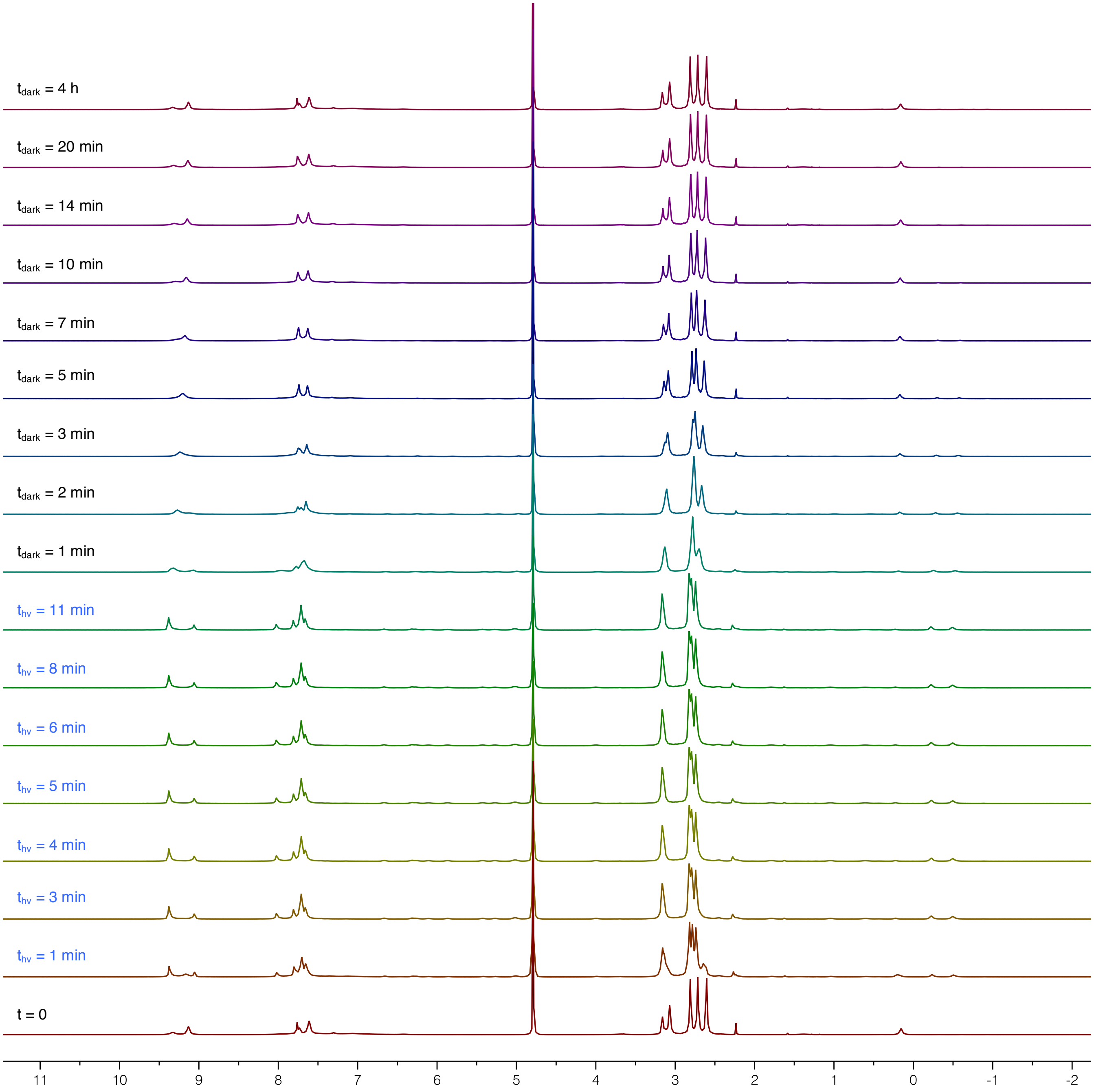

Supplementary Figure 48 | Following reversible isomerization of **5**$\subset$**4** by NMR. A series of ^{1}H NMR spectra (400 MHz, D_2O) of **5**$\subset$**4** at a concentration of ~15 mg/mL following exposure to blue light (λ = 460 nm) inside the NMR spectrometer for different periods of time (t_{hv} = 1 min, 3 min, etc.) for up to 11 min and after thermal relaxation at room temperature in the dark (t_{dark} = 1 min, 2 min, etc.) for up to 4 hrs.

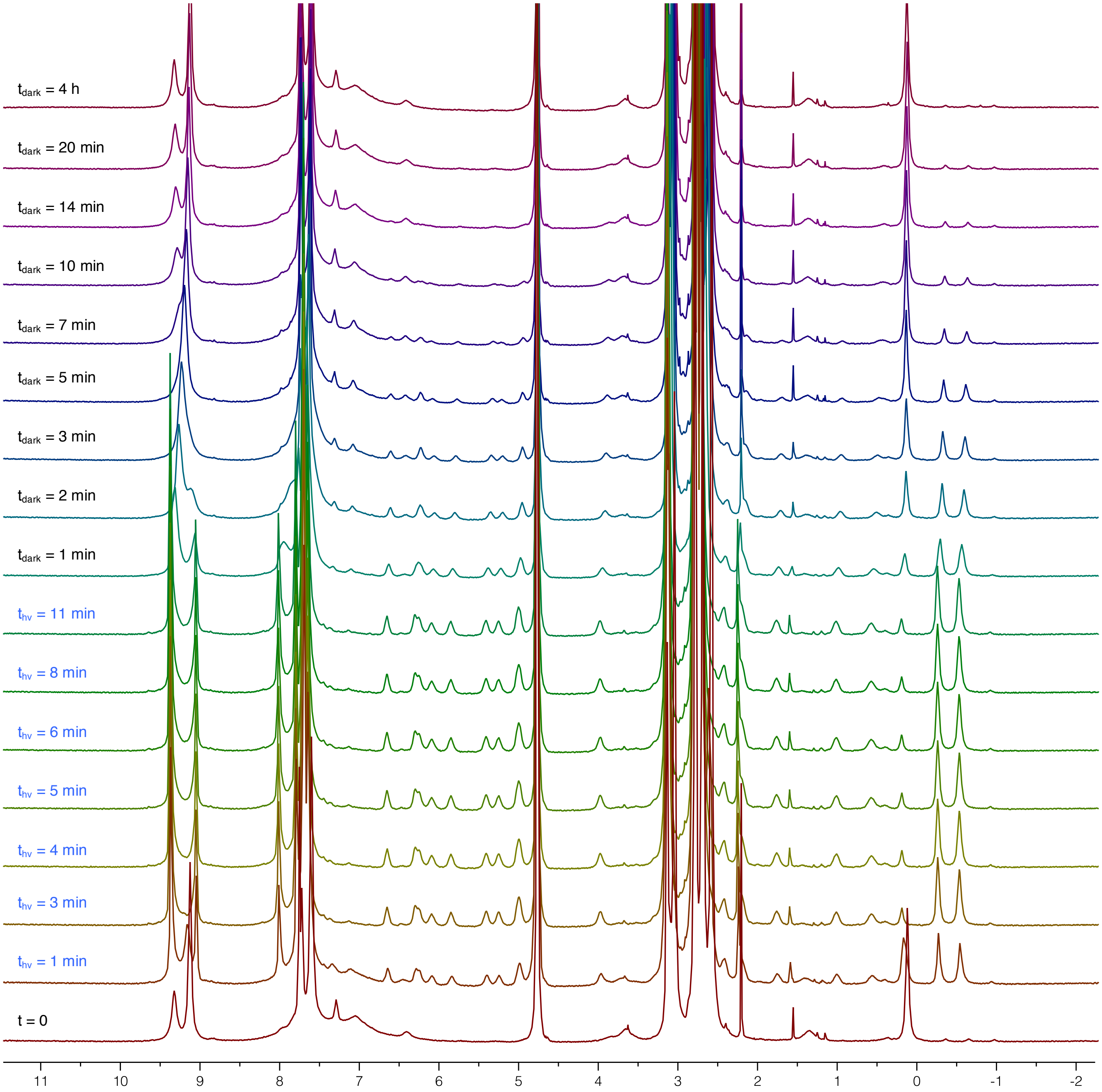

Supplementary Figure 49 | A series of ^{1}H NMR spectra (400 MHz, D_2O) of **5⊂4** at a concentration of ~15 mg/mL following exposure to blue light (λ = 460 nm) inside the NMR spectrometer and after dark incubation. The spectra shown here are magnified from Supplementary Fig. 48. The signal at 0.14 ppm can be assigned to the C(C**H**$_3$)$_2$ protons in the MCH^+ form; the signals at –0.33 ppm and –0.62 ppm can be assigned to the C(C**H**$_3$)$_2$ protons in the SP form.

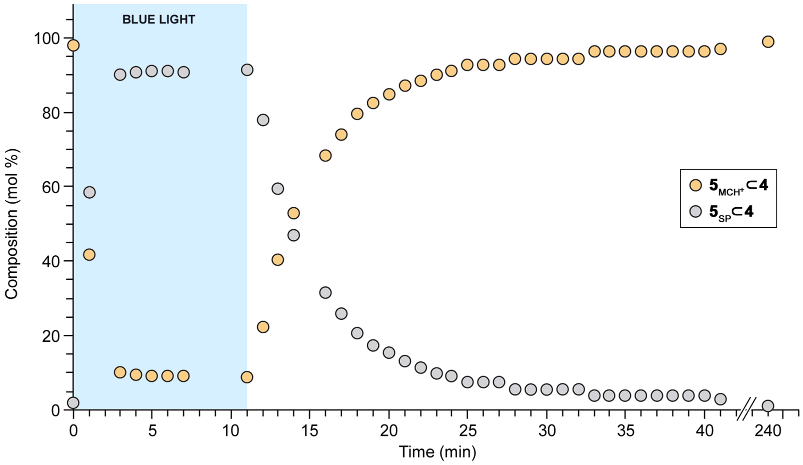

Supplementary Figure 50 | Kinetics of the light-induced ring-closing reaction and the subsequent ring-opening reaction in the dark. Plotted based on the data shown in Supplementary Fig. 48/49.

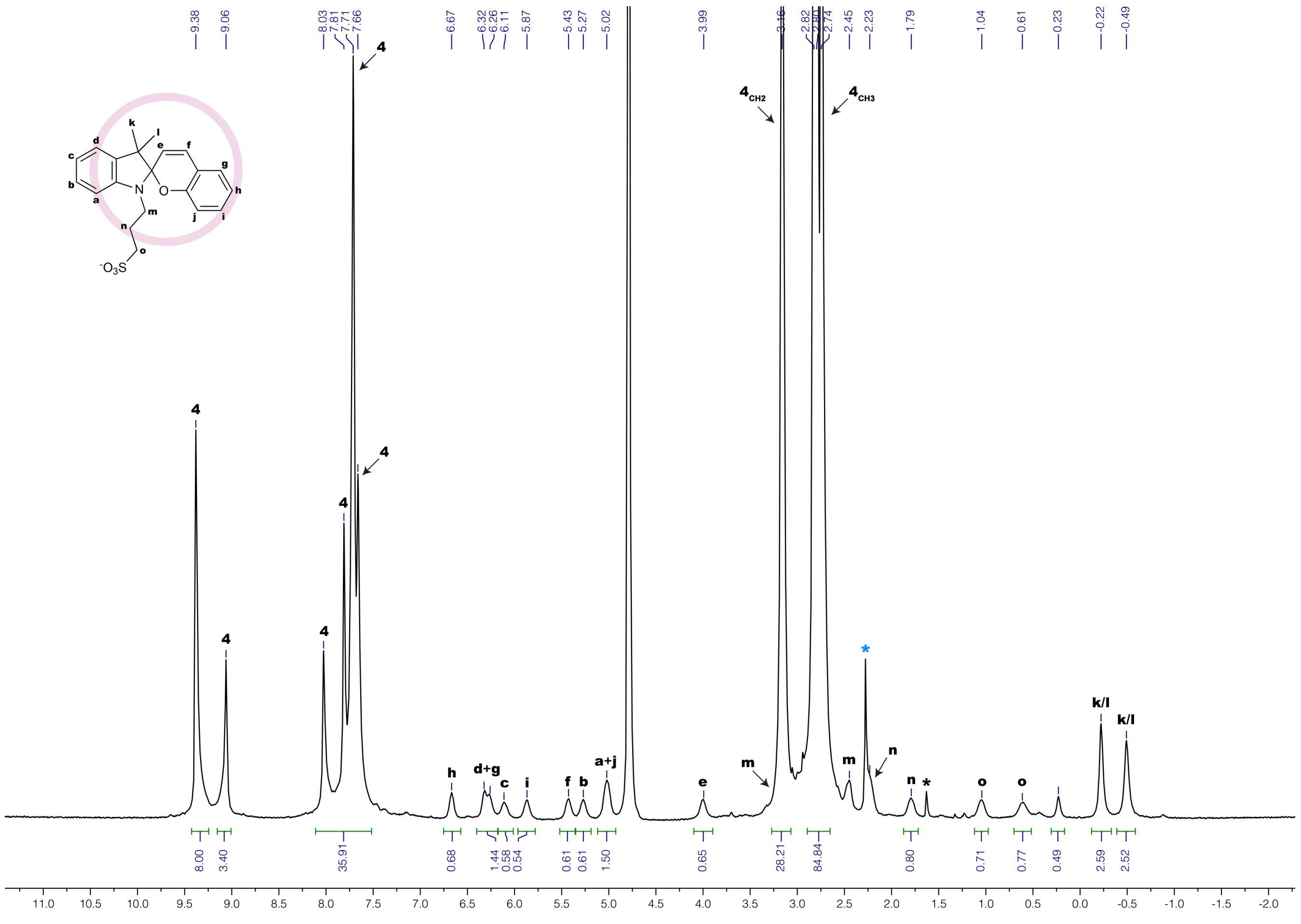

Supplementary Figure 51 | ^{1}H NMR spectrum of **5⊂4** (500 MHz, D_2O) recorded during irradiation with blue light inside the NMR spectrometer (using an optical fiber). Blue asterisk denotes acetone. Black asterisk denotes a contamination. The signal at ~0.25 ppm corresponds to residual $\mathbf{5}_{MCH+}$.

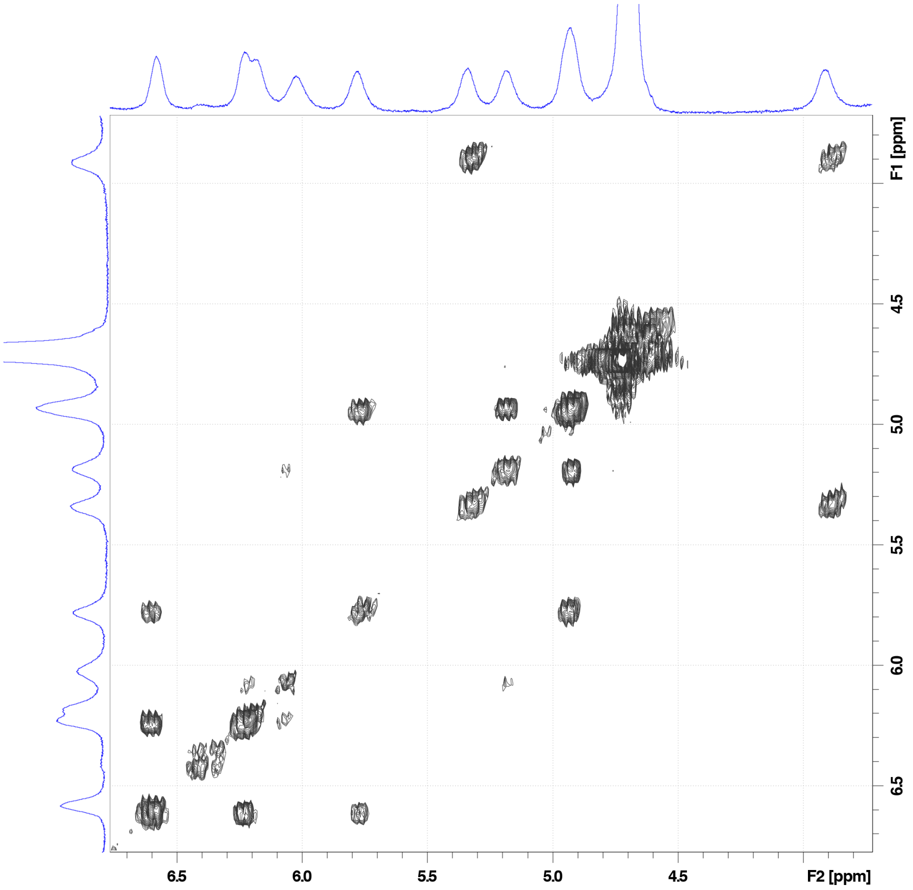

Supplementary Figure 52 | Partial ^{1}H-^{1}H COSY spectrum of **5**$\subset$**4** (500 MHz, D_2O) recorded during irradiation with blue light inside the NMR spectrometer (using an optical fiber).

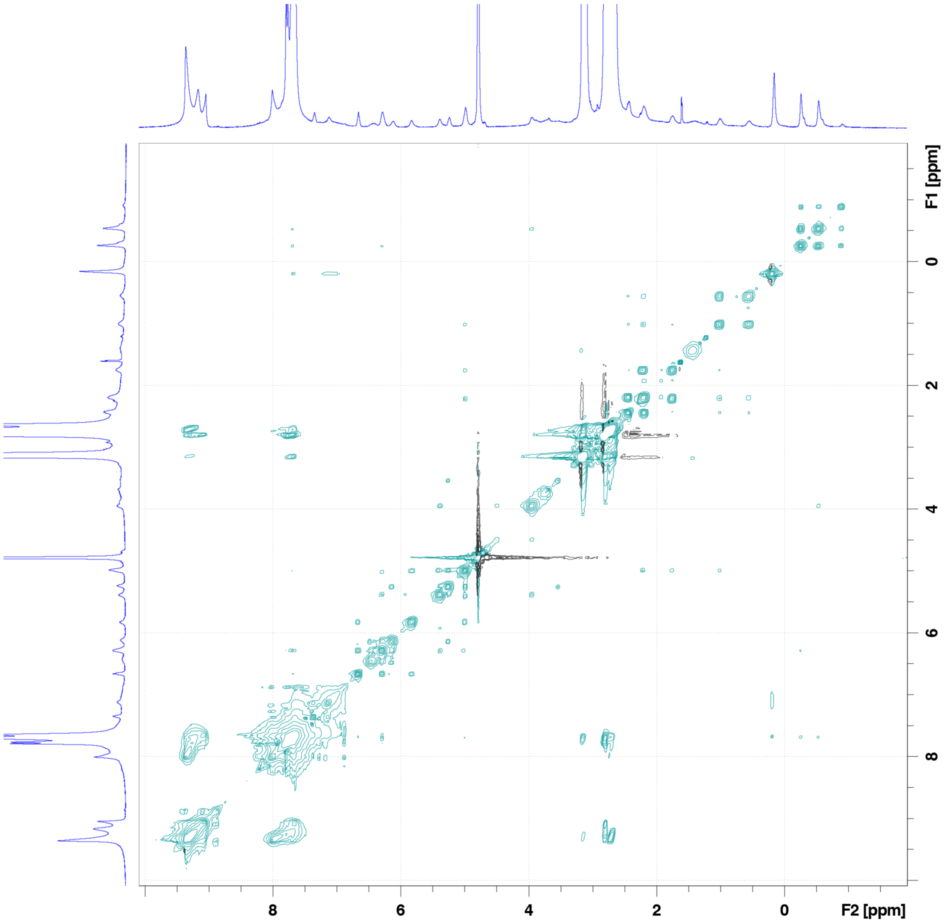

Supplementary Figure 53 | ^1H-^1H NOESY NMR spectrum of **5⊂4** (500 MHz, D_2O) recorded during irradiation with blue light inside the NMR spectrometer.

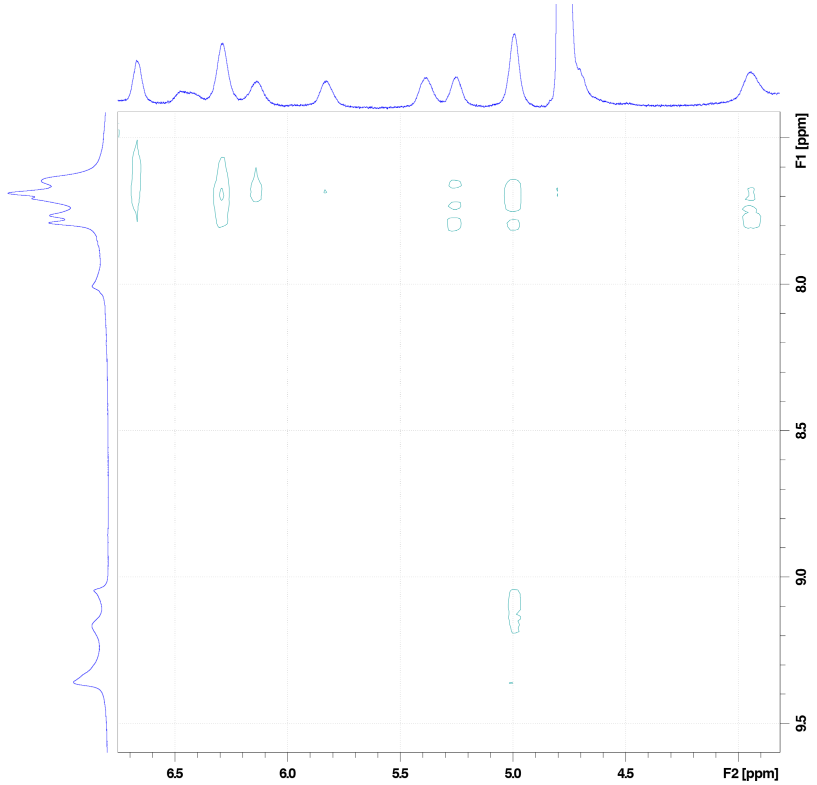

Supplementary Figure 54 | Partial ^{1}H-^{1}H NOESY NMR spectrum of **5**⊂**4** (500 MHz, D_2O) recorded during irradiation with blue light inside the NMR spectrometer. The spectrum focuses on the range showing host-guest correlations (the corresponding full-range spectrum is shown in Supplementary Fig. 53).

Supplementary Note 15 | The effect of pH on the rate of light-induced decoloration of **5**⊂**4**.

To demonstrate the involvement of H^+ in light-induced isomerization of **4**-encapsulated $\mathbf{5}_{MC}$, we irradiated **5**⊂**4** (*c* = 0.067 mM) dissolved in three different buffer solutions: at pH = 6.6 (obtained by mixing solutions of Na_2HPO_4 and citric acid; final concentrations = 138 mM and 26 mM, respectively), pH = 7.0 (156 mM Na_2HPO_4 + 17 mM citric acid), and pH = 7.4 (172 mM Na_2HPO_4 + 9 mM citric acid). One-milliliter aliquots of the solutions were exposed to 460 nm LED radiation and the absorbance at 592 nm was recorded. A direct $\mathbf{5}_{MC}\subset\mathbf{4} \rightarrow \mathbf{5}_{SP}\subset\mathbf{4}$ transformation should not be affected by solution pH; however, we observed pronounced acceleration of the reaction with decreasing pH, suggesting that the reaction is catalyzed by H^+ according to the equation, $\mathbf{5}_{MC}\subset\mathbf{4} + H^+ \leftrightarrows \mathbf{5}_{MCH+}\subset\mathbf{4} \rightarrow \mathbf{5}_{SP}\subset\mathbf{4} + H^+$.

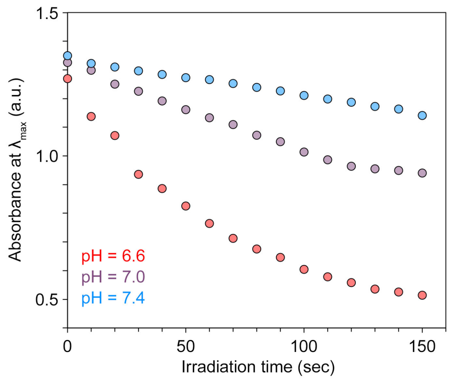

Supplementary Figure 55 | pH-dependent decrease of absorbance at the wavelength of maximum absorption (592 nm for **5**⊂**4**).

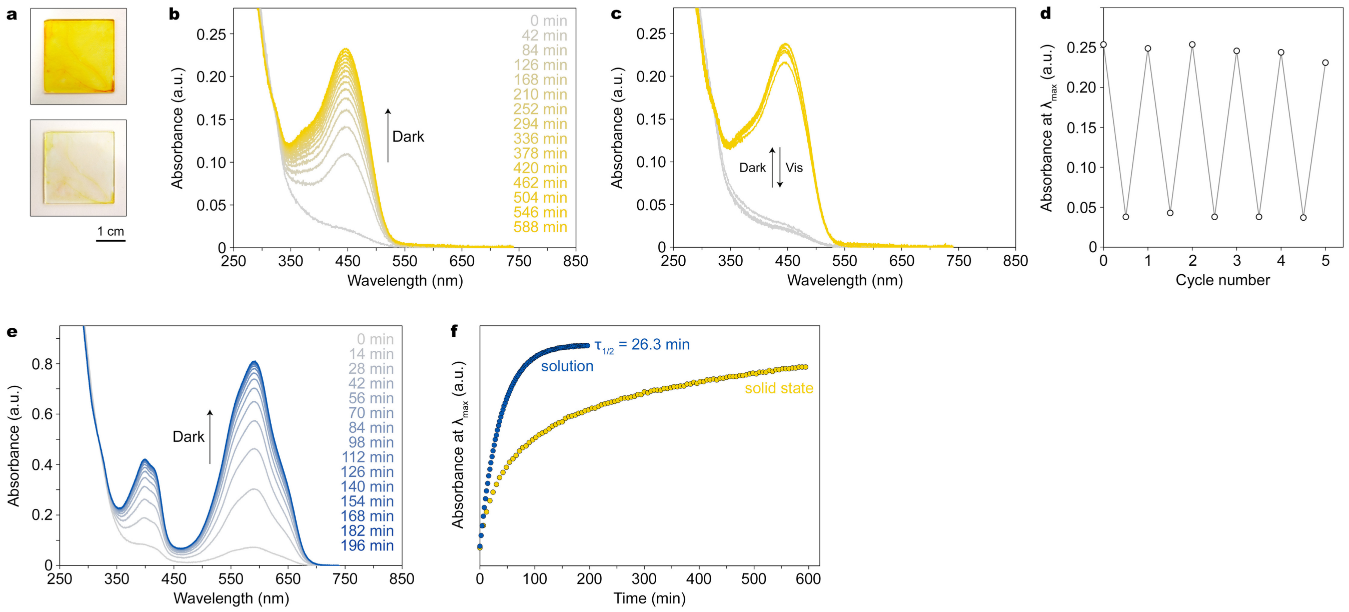

Supplementary Figure 56 | Reversible photochromism of 4-encapsulated 5 in the solid state. a, Thin film of **5**⊂**4** deposited on a glass slide before (top) and after (bottom) exposure to a low-intensity blue LED for 1 min. **b,** A series of solid-state UV/Vis spectra accompanying the spontaneous (dark) SP→MCH^+ ring-opening reaction occurring within **5**⊂**4** deposited as a thin film on a glass slide. Initially, the film was exposed to blue light for 1 min, resulting in the spectrum shown in gray (0 min). Subsequent spectra were recorded at the times indicated on the right. **c,** Reversible switching between the SP and the MCH^+ forms for five cycles. The UV/Vis spectra were recorded following alternating exposure to blue light and dark incubation. **d,** Reversible switching of absorbance at λ_{max} (= 446 nm for the MCH^+ form in the solid state) (plotted based on the data shown in **c**). **e,** A series of UV/Vis spectra following the spontaneous (dark) SP→MC ring-opening reaction occurring within **5**⊂**4** dissolved in water (replotted from Fig. 4d in the main text). Initially, the solution was exposed to blue light for 1 min, resulting in the spectrum shown in gray (0 min). Subsequent spectra were recorded at the times indicated on the right. **f,** Kinetics of the spontaneous coloration reaction in solution (blue) and in the solid state (yellow) (plotted based on the data shown in **b** and **e**). The solution curve can be fitted to a first-order kinetics equation with a time constant, $\tau_{1/2}$ = 26.3 min. No such fit was possible for the solid-state curve. Absorbance at λ_{max} (y-axis) corresponds to 446 nm for the solid-state samples and to 592 nm for the aqueous solution.

Supplementary Note 16 | X-ray data collection and structure refinement.

Single crystals of cage **4** were obtained by slow vapor diffusion of acetone to an aqueous solution of **4** and those of inclusion complex **5⊂4** were obtained by slow evaporation of water from the respective aqueous solution. The diffraction data were collected on a Rigaku XtaLAB PRO diffractometer and processed with CrysAlisPRO. The intensity data of **5⊂4** were collected using Cu-Kα radiation (1.54184 Å) and of **4** – using Mo-Kα radiation (0.7107 Å). Data collection was performed under LN_2 at 100 K. The structures were solved by direct methods using SHELXT[16]. All non-hydrogen atoms were further refined by SHELXL with anisotropic displacement coefficients[17]. Hydrogen atoms were assigned isotropic displacement coefficients, U(H) = 1.2U(C) or 1.5U (C-methyl), and their coordinates were allowed to ride on their respective carbons. The quality of the obtained X-ray data of the complex **5⊂4** was poor due to the presence of severely disordered solvent molecules and counterions. Crystallographic data and refinement parameters are summarized in Supplementary Table 1.

Species	4	5⊂4
**CCDC No.**	1551434	1551437
**Formula**	$C_{96}H_{144}N_{48}O_{53}Pd_6$	$C_{117}H_{166}N_{40}O_{13}Pd_6S$
**Formula weight**	3457.00	3011.37
**Crystal system**	Triclinic	Monoclinic
**Space group**	$P\bar{1}$	$P2_1/c$
**Crystal size (mm)**	0.176×0.062×0.052	0.200×0.180×0.120
**Crystal color and shape**	Colorless prism	Rectangular yellow
**Temperature (K)**	100	100
**Wavelength (Å)**	0.71073	1.54178
**a (Å)**	12.2561(4)	24.4193(4)
**b (Å)**	15.7911(6)	25.0815(5)
**c (Å)**	22.3200(5)	30.6327(7)
**α (°)**	108.893(3)	90
**β (°)**	102.292(2)	107.049(2)
**γ (°)**	95.182(3)	90
**Volume (Å^3)**	3933.6(2)	17937.2(6)
**Z**	1	4
**$\rho_{calcd,}$(g cm^{-1})**	1.459	1.155
**μ (mm^{-1})**	0.763	5.300
**No. of reflection (unique)**	73768 (17964)	126975 (28568)
**R_{int}**	0.0595	0.0722
**Completeness to θ (%)**	99.7	99.9
**Data / restraints / parameters**	17964 / 20 / 907	28568 / 15 / 1426
**Goodness-of-fit on F^2**	1.069	1.416
**Final R_1 and wR_2 indices [$I > 2\sigma(I)$]**	0.0889, 0.2366	0.1275, 0.3502
**R_1 and wR_2 indices (all data)**	0.1161, 0.2546	0.1403, 0.3613

Supplementary Table 1 | Crystallographic data.

Supplementary References

1. Fujita, M., Oguro, D., Miyazawa, M., Oka, H., Yamaguchi, K. & Ogura, K. Self-assembly of 10 molecules into nanometer-sized organic host frameworks. *Nature* **378**, 469-471 (1995).
2. Shi, Z., Peng, P., Strohecker, D. & Liao, Y. Long-lived photoacid based upon a photochromic reaction. *J. Am. Chem. Soc.* **133**, 14699-14703 (2011).
3. Kundu, P. K., Samanta, D., Leizrowice, R., Margulis, B., Zhao, H., Börner, M., Udayabhaskararao, T., Manna, D. & Klajn, R. Light-controlled self-assembly of non-photoresponsive nanoparticles. *Nat. Chem.* **7**, 646-652 (2015).
4. Amdursky, N., Kundu, P. K., Ahrens, J., Huppert, D. & Klajn, R. Noncovalent interactions with proteins modify the physicochemical properties of a molecular switch. *ChemPlusChem* **81**, 44-48 (2016).
5. Hajjaj, F., Tashiro, K., Nikawa, H., Mizorogi, N., Akasaka, T., Nagase, S., Furukawa, K., Kato, T. & Aida, T. Ferromagnetic spin coupling between endohedral metallofullerene La@C_{82} and a cyclodimeric copper porphyrin upon inclusion. *J. Am. Chem. Soc.* **133**, 9290-9292 (2011).
6. Hristova, Y. R., Smulders, M. M. J., Clegg, J. K., Breiner, B. & Nitschke, J. R. Selective anion binding by a "Chameleon" capsule with a dynamically reconfigurable exterior. *Chem. Sci.* **2**, 638-641 (2011).
7. Takezawa, H., Akiba, S., Murase, T. & Fujita, M. Cavity-directed chromism of phthalein dyes. *J. Am. Chem. Soc.* **137**, 7043-7046 (2015).
8. Thordarson, P. Determining association constants from titration experiments in supramolecular chemistry. *Chem. Soc. Rev.* **40**, 1305-1323 (2011).
9. Biedermann, F., Uzunova, V. D., Scherman, O. A., Nau, W. M. & De Simone, A. Release of high-energy water as an essential driving force for the high-affinity binding of cucurbit[*n*]urils. *J. Am. Chem. Soc.* **134**, 15318-15323 (2012).
10. Biedermann, F., Nau, W. M. & Schneider, H.-J. The hydrophobic effect revisited—Studies with supramolecular complexes imply high-energy water as a noncovalent driving force. *Angew. Chem. Int. Ed.* **53**, 11158-11171 (2014).
11. Yoshizawa, M., Kusukawa, T., Kawano, M., Ohhara, T., Tanaka, I., Kurihara, K., Niimura, N., Fujita, M. Endohedral clusterization of ten water molecules into a "molecular ice" within the hydrophobic pocket of a self-assembled cage. *J. Am. Chem. Soc*. **127**, 2798-2799 (2005).
12. Samanta, D., Shanmugaraju, S., Joshi, S. A., Patil, Y. P., Nethaji, M. & Mukherjee, P. S. Pillar height dependent formation of unprecedented Pd_8 molecular swing and Pd_6 molecular boat via multicomponent self-assembly. *Chem. Commun.* **48**, 2298-2300 (2012).
13. Klajn, R. Spiropyran-based dynamic materials. *Chem. Soc. Rev.* **43**, 148-184 (2014).
14. Kundu, P. K., Olsen, G. L., Kiss, V. & Klajn, R. Nanoporous frameworks exhibiting multiple stimuli responsiveness. *Nat. Commun.* **5**, 3588 (2014).
15. Zhu, M. Q., Zhang, G. F., Hu, Z., Aldred, M. P., Li, C., Gong, W. L., Chen, T., Huang, Z. L. & Liu, S. Y. Reversible fluorescence switching of spiropyran-conjugated biodegradable nanoparticles for super-resolution fluorescence imaging. *Macromolecules* **47**, 1543-1552 (2014).
16. Sheldrick, G. M. SHELXT – Integrated space-group and crystal-structure determination. *Acta Crystallogr. A* **71**, 3-8 (2015).
17. Sheldrick, G. M. A short history of SHELX. *Acta Crystallogr. A* **64**, 112-122 (2008).
