# Supplementary material for: Reversible chromism of spiropyran in the cavity of a flexible coordination cage
Source: Nat Commun. 2018 Feb 13;9:641. doi: 10.1038/s41467-017-02715-6 (PMC5811438; doi:10.1038/s41467-017-02715-6)
Supplement: Supplementary file 2 — Description of Additional Supplementary Information [file 41467_2017_2715_MOESM2_ESM.pdf]

Description of Additional Supplementary Files

File Name: Supplementary Movie 1
Description: Atomistic molecular dynamics simulation of cage **2** at room temperature in explicit water. The movie is available for downloading at https://www.dropbox.com/s/ogmnrp27y71buh3/2.mov.

File Name: Supplementary Movie 1
Description: Atomistic molecular dynamics simulation of cage **4** at room temperature in explicit water. The movie is available for downloading at https://www.dropbox.com/s/sa41typlu0j6all/4.mov.
